# Arsenical Cancer: A Review

**DOI:** 10.1038/bjc.1947.22

**Published:** 1947-06

**Authors:** O. Neubauer


### ARSENICAL CANCER: A REVIEW.

O. NEUBAUER.

*Published for the Arsenic Committee of the Medical Research Council.*

Received for publication June 15, 1947.

### TABLE OF CONTENTS.

*A. Medicinal Arsenical Cancer.*

I. Historical.
II. Diseases for which Arsenical Drugs were taken.
III. Sex of the Patients.
IV. Arsenical Drugs Used.
V. Duration of Administration of Arsenic.
VI. Quantity of Arsenic Taken.
VII. Symptoms of Arsenicism Preceding and Accompanying Arsenical Cancer.
VIII. Clinical Pictures of Medicinal Arsenical Cancer.
  1. Arsenical Epithelioma of the Skin Developing in Keratotic Areas (Hutchinson Type or Keratoseogenic Cancer).
  2. Arsenical Epithelioma Arising in a Patch of Psoriasis.
  3. Arsenical Epitheliomas in Normal Skin.
  4. Multiple (Benign) Superficial Epitheliomata.
  5. Bowen's Disease.
  6. Extramammillary Paget's Disease.
  7. Arsenical Cancer in other Organs than the Skin.
  8. Combination of Polymorphous Types.
  9. Frequency of the Different Types of Medicinal Arsenical Cancer.
  10. Multiplicity of Medicinal Arsenical Cancer.
  11. Distribution of Medicinal Arsenical Cancer.
  12. Chronological Course of Medicinal Arsenical Cancer.
  13. Age at Onset of Medicinal Arsenical Cancer.
IX. Histology of Arsenical Cancer.
X. Chemistry and Histochemistry in Arsenical Cancer.
XI. Prognosis of Arsenical Cancer.
XII. Treatment of Medicinal Arsenical Cancer.

*B. Arsenical Cancer Caused by Drinking Water.*

Reichenstein Disease—Cordoba Disease—Other Occurrences.

*C. Occupational Arsenical Cancer.*

I. Arsenical Cancer in Miners and Smelters.
II. Arsenical Cancer in Factories.
  1. Sheep Dip Factories.
  2. Cancer Caused by Arsenical Insecticides.
  3. Arsenical Cancer Caused by Coal and its By-Products.
  4. Cancer of the Bladder in Aniline Workers.
III. Comment on Occupational Arsenical Cancer.

*D. Arsenical Cancer in Animals (except Experimental Cancer).*

Cornwall and Wales—Freiberg—Tharandt Forest—Limbach.

*E. Experimental Arsenical Cancer.*

Local Application on the Skin.
Oral Application of Arsenic.
Parenteral Application of Arsenicals.
Influence of Arsenic on Pre-existing Cancer.
Combination of Arsenic with other Agents (Carrel—Askanazy—Fischer-Wasels).

*F. Pathogenesis of Arsenical Cancer.*

Relations to Keratoses and Pigmentation—Local Effects of Arsenic—General Effects—Action on Capillaries—Action on Metabolism—Stimulation of the Cells—Predilection for the Skin—Prolonged Latent Period—Individual Reactivity—Heredity—Embryonic Germs—Co-operation of Several Factors.

References.

Tables.

### A. MEDICINAL ARSENICAL CANCER.

### *I. Historical.*

THE first observer to direct general attention to the role of arsenical drugs as an etiological factor in cancer of the skin was Sir Jonathan Hutchinson.* He described in 1887/88 five cases leading him to the idea that the internal administration of arsenic in large doses over long periods might produce a form of cancer which presents certain peculiarities. In all cases a condition of local keratosis had preceded the cancer ; in one case the cancerous ulcer had developed on rough skin on the side of the trunk, in one case on a corn on the scrotum, whilst in all the others it occurred in keratotic areas on the hands or feet.

In the following years the views of Hutchinson were discussed. An always rising number of similar cases was reported. Hartzell (1899) was able to collect 10 cases ; Wile (1912) 19 cases ; Pye-Smith (1913) 31 cases, 24 of them of apparently medicinal origin. Nearly all authors (Hartzell, 1899; Ullmann, 1900; Schamberg, 1907; Dubreuilh, 1910; Pye-Smith, 1913; Bland-Sutton, 1916; Wise, 1920) agreed with the teaching of Hutchinson. Wile (1912) made an " impartial statement " of the facts for and against the specificity of arsenical cancer. Only a few authors, as de Silva (1918), opposed the views of Hutchinson ; de Silva admitted that arsenic is responsible for the production of the keratotic condition, but regarded the development of cancer as being independent of the drug. Eventually the views of Hutchinson were accepted by all dermatologists.

Then the predominance of a history of psoriasis in cases of arsenical cancer was especially discussed (see p. 194).

A new period in the history of medicinal cancer started with the establishment and characterization of the clinical picture called multiple superficial epitheliomatosis, and the discussion of the etiological role of arsenic in this condition. These discussions mainly in the dermatological societies since about 1930 have not yet led to general agreement (see p. 201). The same is true

* Preceding the appearance of Hutchinson's report several authors had already described the occurrence of cancer of the skin in cases of psoriasis (Pozzi, 1874 ; Tillaux, 1877 ; Cartaz, 1878 ; White, 1885 ; and Hebra, 1887 ; *cit.* Pye-Smith, 1913*b*) ; but they had not associated the development of cancer in these cases with the previous administration of arsenicals for treatment.

concerning the condition called Bowen's disease and extramammillary Paget's disease and their relations to ingestion of arsenic. It was tried to clear these questions by the application of chemical and especially histochemical methods of investigation for arsenic in the growths and in the unaffected skin of the patients.

In Table I are collected 143 published cases of medicinal arsenical epithelioma in man. Not included are :

(*a*) About 13 cases in which neither the original paper nor a reliable abstract could be obtained : Toeroek, 1891 ; Crocker-Hartzell, *cit.* Hartzell, 1907 ; Foenss, 1921, 1923 (two cases ?) ; Schaumann, 1922, 1923 ; Papagaaw, 1926 ; Urbach, 1926 ; Ullmann, 1930, 1933 ; Mayer, 1933 ; Schaerrer, 1934 ; Simons, 1936/37 ; Zieler, 1938, *cit.* Voss, 1939 ; Koerbler, 1939 ; Rille, *cit.* Voss, 1939 ; Hauser and Simon, 1941.

(*b*) Cases published without detail : Spencer, 1913 ; Oliver and Finnerud, 1928, " case 2 " ; Montgomery, 1935 (about 11 cases) ; Rosen, discussion to Montgomery, 1935 (several cases) ; Cannon, discussion to Montgomery, 1935 ; Nomland, *cit.* Waugh and Scull, 1935 (two cases) ; Anderson, discussion to Montgomery and Waisman, 1941 (a number of cases) ; Gammel, discussion to Barney, 1944 ; Ellis, discussion to Anderson and Burpeau, 1945 (two cases) ; Roxburgh, discussion to Russell and Klaber, 1945.

(*c*) About 15 cases in which the administration of arsenicals is either doubtful or not mentioned at all, but may be assumed as probable : Pozzi, 1874 ; Tillaux, 1877 ; Cartaz, 1878 ; Whitfield, 1906 ; Sargent, 1906 ; Wende, 1908 ; Remenovsky, 1921 ; Ketron, 1927 (two cases) ; Dreyer, 1927, *cit.* Buergener, 1939 ; Stillians, 1930, *cit.* Anderson, 1932 ; Cheever, 1931, *cit.* Buergener, 1939 ; Franseen and Taylor, 1934 (5 cases, " cases 11–15 ") ; Guszman, 1934, *cit.* Buergener, 1939 ; Opfer, 1935, *cit.* Buergener, 1939 ; Montgomery and Waisman, 1941, " case 3."

(*d*) Four cases in which the diagnosis cancer (epithelioma) is not certain : Schamberg, 1907, " case 2 " ; Sawyer, 1929 ; Lane, 1939 ; Abramowitz, Mattice and Bottvinck, 1944, " case 8."

(*e*) Seven cases in which besides arsenic other potential carcinogenic agents (X-rays, radium) were used : Schwartz and Busman, 1924 (X-rays) ; Mayer, 1927, *cit.* Buergener, 1939 (X-rays) ; Stillians, 1931 ; Blumenthal, 1931, *cit.* Buergener, 1939 (X-rays) ; Milch, 1932 (radium) ; Glen, 1945 (radon) ; Anderson and Burpeau, 1945 (X-rays).

### *II. Diseases for which Arsenical Drugs were taken.*

Considering the vast number of conditions in which arsenic is used for long periods, the great percentage of cases in which arsenical cancer has developed in patients suffering from skin-diseases (in 89 = 71·2 per cent), and especially from psoriasis (in 67 = 53·6 per cent), of all cases with noted diagnosis, is very impressive. It seems to be more than a mere coincidence. Hartzell, 1899; Wile, 1912 ; Alexander, 1921 ; Remenovski, 1921 ; Templeton, discussion to Alderson (1935) ; and Buergener, 1939, have discussed this question (see p. 200). On the other hand the number of cases developed in arsenic-treated cases of different internal disorders is relatively small ; surely some of these diseases may have

Out of the 143 cases in Table I, the disease for which arsenic has been administered is mentioned in 125 cases :

| | | | |
|---|---|---|---|
| For Psoriasis | 67 | cases . | . 53·6 per cent |
| Pemphigus | 3 | 22 cases, (Pemphigus to Unknown disease of skin) | 17·6 ,, |
| Eczema, Dermatitis | 4 | | |
| Dermatitis herpetiformis | 4 | | |
| Acne | 6 | | |
| Lichen ruber | 1 | | |
| Pruritus | 1 | | |
| Unknown disease of skin | 3 | | |
| Sum of cutaneous diseases | 89 | cases. | 71·2 |
| Anaemia and haemorrhagic diathesis | 4 | cases. | |
| Asthma and chronic bronchitis | 6 | ,, | |
| " Stomach trouble " | 1 | ,, | |
| Epilepsy and " fits " | 11 | ,, | |
| Chorea | 3 | ,, | |
| Ataxia (?) | 1 | ,, | |
| Nervousness | 3 | ,, | |
| As " tonic " and for " complexion " | 4 | ,, | |
| Malaria | 1 | ,, | |
| " Syphilis " | 2 | ,, | |
| Sum of diseases without diseases of the skin | 36 | ,, | 28·8 ,, |
| Diseases not known in | 17 | ,, | |
| | | | 100 ,, |

ended with death before an arsenical cancer had time to develop as in cases of pernicious anaemia, leukaemia, and Hodgkin's disease; but this explanation would not be valid for the less dangerous forms of anaemia, for asthma and the great number of patients with asthenia in which prolonged and repeated medication with arsenic is or was in common use. And the appearance of arsenical cancer as a sequel to treatment of syphilis with arsenicals is really a rarity. Only 2 or perhaps 3 cases (50, 53, 108 ?) are contained in Table I. Besides, Cannon (discussion to Montgomery, 1935) mentioned several patients with multiple basal-celled epitheliomas who had received injections of arsphenamine for syphilis, but no details were given. Spencer (discussion to Pye Smith, 1913*a*) mentions another case.

So it seems that affections of the skin, and especially psoriasis, give a predisposition for arsenical cancer, although there is no real definite proof.

### *III. Sex of the Patients.*

In 131 cases of Table I the sex is noted ; 91 were males and 40 females ; that means a sex relation of 2·3 : 1. This predominance of males is not a characteristic of arsenical cutaneous cancer, as it is found in cancer of the skin in general.

### *IV. Arsenical Drugs Used.*

In nearly all cases *inorganic drugs* were used, containing arsenic in its *trivalent form* ; that means derivatives of arsenious acid, the most common drug being potassium arsenite $KAsO_2$ ; it was mostly given in aqueous solution (1 per cent calculated as $As_2O_3$ = Fowler's solution), or as arsenious anhydride = arsenic trioxide $As_2O_3$ in form of the so-called asiatic pills = black pills (each containing usually $\frac{1}{12}$ gr.), or as Donovan's solution (containing 1 per cent $AsI_3$ plus 1 per cent $HgI_2$. In two cases " Ascato " was taken, a patent medicine for the treatment of asthma ; in one case " Dr. Greene's Nervinum." Nearly always the drugs were ingested by mouth ; in two cases they were also injected.

*Pentavalent inorganic* compounds—derivatives of arsenic acid, $H_3AsO_4$, are not in use as medicaments ; so it is not surprising that no case is known in which arsenical medicinal cancer has been observed on this basis ; but, as it is known that pentavalent arsenicals are reduced in the body into trivalent ones (see p. 230), it can be understood that they play a role in occupational cancer (see p. 217).

Some confusion has arisen in the literature of arsenical cancer about the denominations " trivalent " and " pentavalent." It seems to be a mistake that Osborne (1925, 1928) quoted As in arsenious acid as " quintavalent " (pentavalent), and some authors (Milch, 1932 ; McNeer, 1934, and Montgomery, 1935) followed him. Generally arsenious acid is considered as trivalent, although some reasons may be produced for its being pentavalent in the vitreous arsenious anhydride (Erdmann, 1904).

The most important *trivalent organic* arsenical compounds, the arsphenamins so generally used in the treatment of syphilis, play only an insignificant role in the causation of cancer. It is true that arsenical keratoses after their administration have been observed repeatedly (MacLeod, 1913 ; Gauvin, 1927 ; and Timberlake, 1929). Spencer, discussion to Pye-Smith (1913*a*), was told of a case of arsenical epithelioma traceable to salvarsan, and Galloway (*ibid.*,) and Ullmann (1917) have sounded warnings that more incidences might be expected. In the case of Harbitz, 1927 (case 53), after injections of arsphenamine a malignant tumour developed at the site of the injection, a fibrosarcoma, different from the usual kind of arsenical cancer. Rosen (discussion to Montgomery, 1935) has seen several cases in which the treatment of syphilitic affections of the mouth with arsphenamine had led to the development of epitheliomas of the oral mucosa. Cannon (*ibid.*, 1935) has observed basal cell epitheliomas in several patients who had received many injections of arsphenamine a number of years before. Besides, arsphenamines were administered in the cases of Levin (1926) (case 50) and of Milch (1932), but in combination with trivalent inorganic arsenicals. For Ebert's (1929) results with intradermal application of arsphenamine see p. 226, Experimental Arsenical Cancer.

Some *pentavalent organic* arsenicals as cacodylates, atoxyl and arsacetine are—or were—frequently used as medicaments ; but it seems that they never have proved as carcinogens ; only in case 135 possibly cacodylates were used.

No case in known in which *external* application of arsenic as medicament (as a caustic, e.g. in lupus, or in the odontologic practice) has produced a cancer.

### *V. Duration of Administration of Arsenic.*

Duration of exposure to arsenic in cases of medicinal arsenical cancer is reported only in a certain number of the cases. Among 96 cases of Table I the distribution was as follows :

| | |
|---|---|
| 6 weeks | 3 cases. |
| 2 months | 1 case. |
| 6 ,, | 4 cases. |
| Up to 1 year | 3 ,, |
| 1–5 years | 22 ,, |
| 6–10 years | 13 ,, |
| 11–20 ,, | 16 ,, |
| 21–30 ,, | 8 ,, |
| More than 30 years | 4 ,, |
| " A short time " | 2 ,, |
| " A considerable time " | 1 case. |
| " From time to time " | 1 ,, |
| " Several years " | 4 cases. |
| " Many years " or " long time " | 16 ,, |
| | 98 ,, |

In the great majority cancer resulted only after the drug had been administered for a very long period, up to 36 years (case 38). But there are also cases in which a very short time of exposure was sufficient for the later development of malignancy (cases 37, 79, 91, 123 and 128).

### *VI. Quantity of Arsenic Taken.*

Similar great differences can be noted in comparing the doses of arsenic leading to the development of malignancy. Only in about 16 of the patients in Table I the doses and the time of administration are sufficiently noted ; in one case (case 83) a quantity of only 0·19 g. $As_2O_3$ was given, whereas case 23 received in all about 70 g., and case 124 about 121 g.. The average quantity was about 28 g.—about 430 grains.*

### *VII. Symptoms of Arsenicism Preceding and Accompanying Arsenical Cancer.*

The development of arsenical cancer is frequently—often for many years—preceded by non-malignant manifestations of arsenicism. Some of these seem to be without any direct relation to the origin of malignancy, whereas others, especially the keratoses, often give rise to the appearance of cancer, and have, therefore, to be regarded as really " precancerous " manifestations.

Only rarely signs of acute arsenical poisoning are mentioned in the history ; and even certain usual signs of chronic arsenicism, as neuritis, anaemia, nephritis and affection of the liver, have been observed in only a few of the patients with arsenical cancer.

* It is supposed that Fowler's solution, given in the great majority of cases, was always undiluted (1 per cent $As_2O_3$).

A sign of chronic arsenicism in those patients, often but not constantly reported, is *hyperpigmentation* (melanosis) of the skin. The patients exhibit a brownish-yellow tint usually most marked in places which normally are more pigmented (areolae of the nipples, about the axilla, in the groins, on the perineum, etc.), and in sites of pressure (waist of women, beneath the garter, etc.), and on previous lesions of the skin. The pigmentation occurs in patches, often forming reticular mottling, or showing a "rain-drop"-like appearance. The pigmentation is not unlike that in Addison's disease; but in this condition the affected skin is not so harsh and scaly, and often the mucous membranes are affected too (in arsenical melanosis only exceptionally).

In 25 of the cases in Table I hyperpigmentation is noted; but there may be many more in which it was present. Only in 11 cases the absence of pigmentation is definitely stressed.

Other changes of the skin reported are: telangiectases (cases 20, 85, 117 and 124), chronic arsenical dermatitis (case 48), erythrodermia (case 47), erythrodermia exfoliativa (cases 51, 69 and 70), multiple seborrhoeic warts and molluscum fibrosum (cases 140 and 141), seborrhoeic keratoses (cases 74 and 75), and affections of the nails (see Willcox, discussion to Hamilton, 1921).

But by far the most important manifestations preceding the appearance of arsenical cancer are *keratoses*. Henoch and Romberg in 1851 (Geyer, 1898) have recognized epidermic desquamation of palms and soles as a sequel to the internal use of arsenic, and in 1868 Erasmus Wilson has given a specified description of this condition. Hutchinson (1887) has emphasized their importance as precursors of arsenical cancer. (Pictures, see Hutchinson, 1888, plates xxi and xxii, 1895, plates xvii and xix.)

These keratoses occur especially on the palms and soles, but may be found anywhere on the cutaneous surface. The most characteristic form, the nodular form (punctate keratosis) of the palms and soles, is marked by numerous small horny corn-like elevations usually 2–5 mm. in diameter. They occur frequently as epidermal pegs which can be picked out of their keratotic beds. They are frequently situated on the thenar and lateral borders of the hand, on the roots or on the side surfaces of the fingers, sometimes also on the back of the phalangeal joints. On the feet the sites of predilection are the heels and the toes. There may be a confluence of a group of these corns forming wart-like excrescences, but differing from true warts by having no papillary structure. Sometimes the palms only or the soles only are affected. In other cases there is a more diffuse keratosis of the palms and soles, giving the skin a leathery appearance, often accompanied with or preceded by hyperidrosis. The horny thickenings may be situated on an erythematous base, or a horny patch may be surrounded by an erythematous halo. Frequently the diffuse form of keratosis is combined with the nodular form.

The keratoses of palms and soles are usually symmetrically distributed. Fissures are liable to occur on the keratoses.

Slight keratoses may also occur on other parts of the body, rendering the skin harsh, or flat reddish erythematous patches, or scaling keratotic plaques may be found. They occur mostly on the unexposed parts of the body, in contradistinction to the keratoses of senile and of weather-beaten persons.

These keratoses are present in the great majority of cases of arsenical cancer. They are noted in 116 out of the 143 cases of Table I. In only 10 cases their presence is denied; all these are apparently cases of superficial epitheliomas (see below), one perhaps a true psoriasis cancer (case 24, see below). In the remaining 18 cases nothing is said about keratoses, the histories often being short and deficient. So it can be stated that keratoses occur in from 81 to 93 per cent of all cases of arsenical cancer. Hands or feet or both were involved in at least 97 cases, mostly in the classic form of corns on palms and soles, frequently combined with keratoses on other parts of the body. In only two or three cases keratoses were present but spared hands and feet. In case 74 a keratosis appeared in the form of a cutaneous horn in the right groin; in case 47 a distinct hyperkeratosis subungualis is noted. Leukoplakia oris was present in cases 33 and 129.

The keratoses develop gradually, and may not appear for some time after the ingestion of arsenic. The interval between the beginning of exposure to the poison and the appearance of keratotic lesions is very different in different cases. (See p. 210, Chronological Course.)

Many of the keratotic areas become stationary, or may even undergo a gradual involution after the use of the drug has been stopped (Montgomery, 1935). But some of them develop progressive changes—painful fissures, infections, etc.—and eventually may give rise to malignant growth; in some cases the malignancies originate on other parts of the body (see below). Montgomery (1935) states that out of his 85 cases of arsenical keratosis 20 per cent developed epitheliomas.

The multiple horny keratoses, especially the punctate or warty form on the palms and soles, are generally recognized as being pathognomonic for chronic arsenical poisoning; they can be confounded only with certain hereditary forms of keratosis punctata. Nearly all dermatologists agree that their mere presence justifies the diagnosis of arsenicism even in cases lacking any history of ingestion of the poison (see Franseen and Taylor, 1934; Oliver, discussion to G. Lane, 1939; and Montgomery and Waisman, 1941, " case 3 "). Hence their great importance for the diagnosis—as real " leading fossils " for prophylactic therapy and for the consideration of the pathogenesis.

### *VIII. Clinical Pictures of Medicinal Arsenical Cancer.*

The development and appearance of arsenical growths are by no means the same in all cases. There are several types differing in localization, in the grade of malignancy, in the histological picture, etc.

### 1. *Arsenical epithelioma of the skin developing in keratotic areas (Hutchinson type or keratoseogenic cancer).*

These cases, described in such a classic manner by J. Hutchinson, form a most characteristic group. After the keratotic lesions, especially those of the hands and feet, have existed for a long time, sometimes for years, one of them—or some of them—begin to undergo certain changes: they become painful, bleeding fissures appear, and after a certain time, sometimes after years, an ulcer appears with hard pearly borders slowly extending without tendency for healing. Histological examination shows an epithelioma usually of the squamous cell type. Sites of predilection are the hands, especially the fingers, palmar and lateral surfaces, thumb, the borders of the palms, the heel and toes, but any arsenical keratosis may give rise to such a malignant degeneration. At the same time or later another malignant ulcer may appear on a keratosis in the vicinity or elsewhere, or even in a non-keratotic area; and then another and so on.* If not treated in time, earlier or later the regional lymph-nodes become infiltrated, and death occurs with the signs of general marasmus. Practically all such cancers arising on pre-existing arsenical keratoses are squamous-celled in type. The first microscopical changes in the malignant alteration of the keratoses consist of keratinization and vacuolization of individual cells in the epidermis (malignant dyskeratosis); further, the continuity of the basal layer becomes destroyed (see Montgomery, 1935, fig. 2*b* and 3). Arsenic can be found regularly by the Osborne method, and sometimes also by the chemical method (see p. 211, Chemistry and Histochemistry).

This type of arsenical cancer, the "Hutchinson type," is the earliest known, the most characteristic, and is very frequent. Nearly all cases reported until 1910 belong to it. Wile wrote still in 1912: "It is highly probable that all arsenical epitheliomata develop from keratotic nodules." About the frequency of this type see below, Frequency of Different Types.

2. *Arsenical epithelioma arising in a patch of psoriasis.*

In a few of the cases in which malignancy developed in cases of psoriasis treated with arsenic, the cancer originated from a psoriatic patch: cases 23, 32, 39, 43, 138 and possibly also 7 and 46.

The occurrence of cancer in cases of psoriasis attracted attention even before Hutchinson had discovered the carcinogenic property of arsenic (see above, p. 193). Hartzell (1899) collected a series of cases, and recognized that in nearly all cases there was evidence of prolonged arsenical treatment and presence of arsenical keratoses. So he arrived at the opinion that in these cases the cancer is a result of arsenical treatment and not of psoriasis. And as the malignancy nearly always developed on the keratoses, it was thought that all incidences of cancer in psoriasis were due to arsenic; not to the psoriasis. On the other hand Wile (1912) stressed the especially high incidence of psoriatics amongst the patients with arsenical cancer: "Considering the vast numbers of conditions . . . in which arsenic is employed, it seems more than a coincidence that of 19 cases, 15 have occurred in conditions (14 of them psoriasis and 1 pemphigus), in which an abnormality of epithelial growth ante-dated the use of the drug." In the same year Gray (1912) published a case of rodent ulcer originating in a patch of psoriasis (case 24). The patient had taken arsenic but without developing arsenical keratoses. Alexander, collecting all 17 cases of cancer in psoriatics, published until 1921, and adding one case of his own, tried to classify them in two groups:

1. Cases of true or genuine psoriasis-cancer ("echte Faelle von Psoriasis-krebs") in which a direct transition of a (mostly single) psoriatic patch in cancer is evident or very probable; here arsenic may not play an etiological role. There may be no history of ingestion of arsenic; keratoses may be absent. These cases are very rare.

2. Cases of arsenical cancer in psoriatics: The malignancy originates on an arsenical keratosis or on other (non-psoriatic) sites, often multiple

* Pictures, see Hutchinson, 1895 and 1903; Montgomery, 1935, etc.

from the beginning. History of arsenic ingestion; arsenical keratoses are present.

Remenovsky (1921), publishing in the same year a case of true psoriasis cancer (arsenic had been taken, but it seems after the beginning of malignancy), emphasized the extreme rarity of such observations.

Buergener, in 1939, in a critical review goes even so far as to say that a genuine psoriasis-cancer does not seem to exist. In nearly all the cases in which it may be supposed the influence of previous treatment with carcinogenic agents (arsenic, X-rays, radium) may have been of importance. In the remaining very few incidences there may be a chance of an accidental coincidence (two examples are given).

So even in these cases in which the malignancy has developed in a psoriatic lesion, arsenic as a causative agent may be assumed if arsenic has been given (cases 7, 24, 32, 39, 43, 46 and 138), or if its presence is proved by chemical or histochemical methods or if typical arsenical keratoses are found (cases 39, 43 and 46). On the other hand the coincidence of psoriasis and arsenical cancer —including the multiple benign superficial epitheliomas (see below)—is so frequent that some connection, e.g. a predisposition of psoriatics for cancer of the skin, is supposed by many observers (Wile, 1912).

Besides, it must be kept in mind that sometimes other lesions, especially erythematous plaques of superficial epitheliomatosis, are mistaken for psoriatic plaques (Pfahler, 1927; Montgomery, 1929; Wright and Friedman, 1933; Ellis, discussion to Anderson and Burpeau, 1945); even some forms of true cancer bear a striking clinical resemblance to lesions of psoriasis—psoriasiform basal cell carcinoma (see Sutton, 1939).

3. *Arsenical epitheliomas in normal skin.*

Wile wrote in 1912: " It seems highly probable that all arsenical epitheliomata had their starting-point in keratotic nodules; in no case is there evidence of ar epithelioma arising from the unbroken normal skin." This view cannot be maintained further.

So in Lane's (1894) case (case 7) the first growth appeared on the forearm in apparently healthy skin. It seems that not seldom in the same patient some malignancies arise on keratoses and others on normal skin; but unfortunately precise notes on this point are missing in the majority of cases; frequently nothing is said about the previous appearance of the skin. Further, there is no doubt that the lesions of typical multiple superficial epitheliomas regularly develop in previously normal skin of the trunk and the proximal parts of the extremities (see below).

4. *Multiple (benign) superficial epitheliomata.*

(Multiple carcinoids, Arning, 1922; erythematoid benign epithelioma, Graham Little, 1923; epithelioma erythematoides benignum, Montgomery, 1929 and 1935; carcinoma cutis multiforme; epitheliomatosis.*)

This form of epithelioma of the skin and its relation to arsenic have attracted special interest since about 1930.

* Janeway as far back as 1910 gave a clinical and histological description of this type of epithelioma (Gross, 1931).

It is characterized by the eruption of multiple (usually numerous) superficial epitheliomas in the form of slightly depressed red patches mostly on the trunk and the proximal parts of the extremities, but neck and head, including the scalp, may also be affected. The lesions vary from ill-defined seborrhoea-like crusts to lesions several centimetres or even more in diameter with thick, verrucous, hard, dirty crusts. The removal of the covering crusts reveals a wide rolled indurated pearly border, in typical cases a very faint white wax-like narrow raised margin, flatter than the rolled edge of the lesions in the more malignant forms of epithelioma. (For pictures, see Montgomery, 1929 and 1941.)

Histologically the condition usually shows the picture of basal-celled epithelioma, but sometimes squamous-cell epithelioma and mixed types may be found (see below).

The affection is benign in character ; absence of ulceration over long periods ; long duration—10, 20, 30 years and even more ; the lesions sometimes disappearing spontaneously ; no metastases. Hence this kind of epithelioma apparently cannot be called a real cancer. But occasionally in the centre of a plaque a fungating squamous-cell epithelioma may develop, resulting eventually in metastases and death ; and besides, not infrequently they associate with other malignant kinds of epidermal growth, e.g. the Hutchinson type of arsenical cancer.

Graham Little (1923) has mentioned the fact that this condition also frequently affects psoriatics, and many others have confirmed this observation : Ormsby and Mitchell (1925), Stillians (1931), Senear (discussion to Stillians, 1931), Wright and Friedman (1933), Parkhurst (discussion to Arday, 1938), Applestein (1941), and Ellis (discussion to Anderson and Burpeau, 1945).

There is considerable controversy regarding the etiological role that arsenic plays in this type of epithelioma.

In 1931 Stillians and Gross presented patients with typical multiple epitheliomas who were treated previously with arsenic (cases 66 and 68). Andrews (1932), reporting another case (73), stressed this fact ; he admits that this fact alone is no proof for the etiological role of arsenic. On the other hand, he succeeded in his case in demonstrating arsenic in the tissue.

Anderson in 1932 reported three cases with exposure to arsenic (in the second case an occupational one), with keratoses of palms and soles ; in two of his cases (74 and 75) arsenic was detected in the urine ; in one (74) a small quantity also in the tissue. Anderson summed up all points speaking in favour of an etiological role of arsenic in the great majority of cases of superficial epitheliomas :

1. The frequent history of previous exposure to arsenic ; of course that is true for all cases of Table I, as this contains only arsenic-treated cases.

2. The frequent simultaneous occurrence of hyperkeratosis of the palms and feet, similar to that in the Hutchinson type of cancer. Among the about 46 uncombined cases in Table I, in 29 the presence of keratoses is stated (but only in 23 location of the keratoses on the hands and feet) ; in 9 cases their absence ; in 8 cases nothing is noted about keratoses. So the typical keratoses of hands or feet were present in at least 50 per cent of the cases. In one case (64) they started before the appearance of the epitheliomas, in case 74 later.

3. The fact that a considerable number of these patients had psoriasis and were treated for it, or for a condition mistakenly diagnosed as psoriasis (see above, pp. 200–201) Among the 46 patients with uncombined superficial epitheliomas in Table I, at least 21 were treated with arsenic for psoriasis. The question whether this treatment is responsible for the epitheliomas or if there is something in psoriasis itself that tends towards the development of these lesions was discussed in 1931: Senear cited two cases in which the ingestion of arsenic could be ruled out.

Pigmentation of the skin of arsenical type is noted in 9 out of the 46 uncombined cases in Table I.

In subsequent papers Anderson (1937, 1943*a*, 1943*b* and 1945) stuck to his opinion. In the discussion to Montgomery's lecture in 1941 he reported some 40 cases of superficial epitheliomas with arsenical keratoses of palms and soles; in one case at least arsenic was found in the urine. A similar case of Anderson and Burpeau (1945) is not included in Table I because X-rays were also administered.

Ayres (1934 and 1943), confirmed the views of Anderson by reporting two cases apparently caused by arsenic (85 and 131). Ayres and Anderson (1934) reported that in examining 44 patients with basal-cell epithelioma—most of them may be supposed to belong to this type—they found punctate keratoses in 75 per cent. Among 19 cases 12 were demonstrated to have arsenic in the urine, and in six others there was a history of exposure to arsenic.

Wright and Friedman (1933), reporting five cases of superficial epitheliomatosis in psoriatics, three of which had used arsenic, denied the etiological role of that drug because none of these patients had keratosis or pigmentation, and arsenic could not be demonstrated by Osborne's method.

Halloran (discussion to Anderson, 1937) and Traub (1937) supported the view of Anderson. Arday (1938) seems to doubt whether arsenic is a causative factor in such cases, whereas Parkhurst (discussion to Arday, 1938) assumes a relation. Cannon's (discussion to Montgomery, 1935) patients with multiple basal-cell epitheliomas gave in the great majority of cases a history of ingestion of arsenic, and had large quantities of arsenic in blood, urine and skin.

Montgomery (1935 and 1941), with the enormous experience of the Mayo Clinic, denied the etiological role of arsenic in true benign epitheliomatosis. He emphasized that the cases of multiple superficial epitheliomas have to be differentiated in two distinct groups:

1. The multiple benign superficial epitheliomatosis proper, characterized especially by a fine threadlike slightly elevated border of the lesions and by benign character, most of the lesions being of the basal-cell type (picture, Montgomery and Waisman, 1941, fig. 5). Amongst 65 cases of this group (16 described in 1929 and 49 in 1935) only one patient remembered to have taken arsenic. So Montgomery does not believe in arsenic as a definite cause of this condition.

2. The secondary form of multiple superficial epitheliomas arising also from non-keratotic areas and occurring also with predilection for the trunk, but marked by simulating lesions of arsenical keratosis, especially by a wide rolled indurated (not a threadlike) border, and tendency to malignancy (picture, Montgomery, 1935, pp. 223, 373, and 1941, fig. 4).

Histopathologically they are mostly squamous epitheliomas, but sometimes also basal-celled ones are found (Montgomery and Waisman, 1941). Nearly all cases of multiple superficial epitheliomas with a previous history of arsenic or with keratoses of palms and soles are put by Montgomery together with this group, setting them apart from true benign epitheliomatosis. Evidence of pigmentation he noted only in a few of these cases. Attributing to arsenic a very important etiological role he described these cases as " arsenical type of epithelioma," a name which may cause confusion with the keratoseogen Hutchinson-type of arsenical epithelioma. Montgomery reported 7 cases in 1935 (e.g. cases 103, 104 and 105) and 4 in 1941 (e.g. cases 127 and 128). Fraser (discussion to Wise, 1929) supported Montgomery's distinction of two groups of epitheliomas of the trunk.

Montgomery tried to prove his view by chemical analysis. In the growths of the second group he found regularly a considerable amount of arsenic by the chemical as well as by the Osborne method (cases 104, 105, 127 and 128). He lays stress especially on the fact that the growths contained more arsenic than the adjacent normal skin of these patients; whereas in the lesions of the true benign group, in the great majority of cases none or little was found, in any case less than in the normal skin.

Montgomery does not deny any relation of benign epitheliomatosis to arsenic. He admitted that in some cases arsenic may be detected by Osborne's method (1935 and 1941). Further, he himself stated in 1935 that the two types of epithelioma may be associated in the same patient, as in 7 of the 16 cases of " arsenical type of epithelioma " (e.g. case 106). But he suggests that in such cases arsenic is not the cause of the benign epithelioma, but presumably only precipitates its development caused by an unknown predisposition.

There is no doubt that many of Anderson's and Ayres' patients belong to Montgomery's second group, the " arsenical type of superficial epithelioma "; but at least some of their cases seem to be true benign epitheliomas. Anderson (1937) doubts whether the distinction between the two groups is really practicable; he " cannot see how anyone can make such a hair-line distinction as regards the border."

Most authors agree now that at least a proportion of superficial basal-celled epitheliomas are caused by arsenic. Opinions differ mainly as to how great this proportion is; whether nearly all incidences of this condition are due to arsenic (Anderson and Ayres), or many of them (Barber, 1939; MacCormac, discussion to Barber, 1939), or a certain percentage (Bloom, 1936; Gray, discussion to Barber, 1939) or only a few (Mumford, *ibid.*, 1939). Andrews (1932) thinks that arsenic may be supposed to play an etiological role, but there is no real proof. Barker (discussion to R. M. Montgomery, 1942) reported lately a high content of arsenic in the blood of such patients.

In a very high proportion of cases multiple superficial epitheliomas were associated with other forms of arsenical cancer (see p .207, Frequency of the Different Types).

Another type of epithelioma is mentioned by Cannon (discussion to Montgomery, 1935): basal-cell epitheliomas on the face of comparatively young persons recurring at intervals of several years in different locations, with presence of large quantities of arsenic. But no details of the histories are given, and the author himself finds it difficult to evaluate the presence of arsenic.

5. *Bowen's disease.*

Much confusion has arisen in the last twenty years about " Bowen's Disease " and its relations to arsenic.

Bowen, in 1912, described an extremely chronic affection of the skin with atypical epithelial proliferation as a new type of " pre-cancerous dermatosis." This Bowen's disease is marked clinically by multiple slightly elevated papules of dull-red colour, covered with scaly crusts, and arranged in groups—often with arciform configuration—or as multiple lenticular plaques. The histological picture is described as a neoplastic process with abnormal keratinization, " malign dyskeratosis " (Darier, 1914–15 and 1920), and amitotic cell division producing giant cells. The changes are often similar to those in arsenical keratosis, but vacuolization of the prickle cells is usually not so pronounced. The neoplastic process may remain intradermal, but sometimes an infiltrating squamous-cell epithelioma with metastases develops (in about 20 per cent of the cases). Fraser (1928)* pointed out that the cellular changes are characteristic of malignancy from the beginning ; therefore the condition has to be classified, not as a " precancerous " dermatosis, but as a real carcinoma, but without infiltrating properties : an intradermal cancer of the squamous-cell type. Wise (discussion to Fraser, 1928), Highman (*ibid.*,) and Ebert and Otsuka (1943) agree with Fraser.

The question whether Bowen's disease is a strict entity was always a controversial subject, especially whether a sharp line can be drawn against multiple superficial epitheliomas. Bosellini (1928) denied this. Anderson held the view that both conditions bear relationship to each other, their simultaneous occurrence in some cases testifying this view. Wise (1920), Eliascheff (*cit.* Barber, 1928), Montgomery (1929, 1935 and 1941), and Allington (discussion to Alderson, 1935) maintain that Bowen's disease is an entity and can be differentiated from multiple superficial epitheliomas. But Montgomery admits that both conditions are found occasionally associated, and that either can be associated with the ordinary arsenical type of epithelioma.

Wise (1928) and Fraser think that a definite diagnosis of Bowen's disease cannot be made on clinical evidence but only by histological examination ; Montgomery and Waisman (1941), on the other hand, state that the same histological structure is found also in other precancerous dermatoses. Montgomery (discussion to Goeckerman and Wilhelm, 1940) emphasized especially the fact that malignant dyskeratosis simulating Bowen's disease is a characteristic feature of the arsenical type of epithelioma. It is a matter of fact that almost no case presented during the last years in a dermatological society has escaped doubts about correct diagnosis (see Schoff, discussion to Alderson, 1935 ; Montgomery about case 74 ; Ebert and Otsuka (1943, discussion) ; and the frequent use of ill-defined terms such as " Bowenoid " or " Bowen-like " indicates the lack of evidence in that matter.

Anderson (1932), having observed a case of Bowen's disease with a history of arsenic, arsenic present in the urine, and keratoses of palms and soles (case 74),

* See also Lane, discussion to Ormsby and Mitchell, 1925.

expressed the opinion that this condition as well as multiple superficial epitheliomas are the result of this poison. Fraser (1927 and 1928) reported a similar incidence.

But H. Montgomery (1935) did not agree; in his five cases of Bowen's disease neither had arsenic been administered nor were keratoses or pigmentation present. He explains Anderson's case as an epithelioma of the arsenical type simulating the clinical picture of Bowen's disease.

R. M. Montgomery (1942) reported a case of Bowen's disease verified by autopsy in which arsenic was taken, with keratoses of fingers and toes (case 129).

Among the cases in Table I in only five the strict diagnosis of Bowen's disease was made by one or another observer (cases 55, 74, 103, 129 and 133); in three of them keratoses were present; in two association with multiple superficial epitheliomas; in one of these also with keratoseogen cancer and two cancers of mucous membranes. Besides, in seven incidences (67, 85, 109, 110, 121, 125 and 134) a " Bowen-like " or " Bowenoid " appearance is noted.

6. *Extramammillary Paget's disease.*

This affection, mostly localized on the genitals, seems to be very similar to, or identical with Bowen's disease (Fraser, 1928; Highman, discussion to Fraser, 1928; and Montgomery, 1929). The relations to arsenic are not clear, but may be the same as in Bowen's disease.

7. *Arsenical cancer in other organs than the skin.*

In some instances arsenical cancer of the skin is associated with cancer in other organs.

Thus one of Hutchinson's (1888) original cases died of cancer of the stomach (case 5). In one of Ullmann's cases (case 33), of Hutchinson type, a cancer of the tongue and later a cancer of the oral mucosa developed. It may be the same case which was mentioned by Mayer (1933). Franseen and Taylor (1934) refer to a case of keratoseogen cancer of the skin with a suspicion of associated cancer oesophagi (case 88).

Associated with multiple superficial epitheliomas and with typical keratoses a cancer uteri occurred in the case of MacCormac (1933*a*, *b*, case 76); with multiple epitheliomas and a squamous-cell epithelioma of the groin and a carcinoma of the tongue in case 123 (Anderson, discussion to Goeckerman and Wilhelm, 1940).

In a case of Montgomery (1935), with association of keratotic epitheliomas and numerous superficial epitheliomas (case 103) an intra-urethral epithelioma developed and later a bronchial carcinoma of " arsenical type."

Goeckerman and Wilhelm (1940) observed a papilloma of ureter and bladder in a case with keratosis of palms and soles and Bowen-like keratotic lesions on the trunk (case 122). In the discussion to Montgomery and Waisman (1941), Anderson mentions one case of Fraser and Fox and another of Goldman and Tauber, both with papilloma of the bladder. Barney (1944) reported a cancer of the oesophagus in a patient with keratoses and multiple superficial epitheliomas beginning in a patch of psoriasis (case 138).

It may be assumed that these localizations on the mucous membranes were also due to arsenic; several authors have made this suggestion (Ullmann, 1922; Goeckerman and Wilhelm, 1940; Montgomery, *ibid.*, discussion; Anderson, *ibid.*), but it is not exactly proved ; a mere coincidence cannot be excluded. Arsenic never was identified in these growths until now.

In some cases after administration of arsenic, cancers in different organs occurred without cutaneous cancer but with arsenical keratoses ; thus a case of cancer of the breast published by Dubreuilh (case 22). Franseen and Taylor observed a cancer of the pancreas in a patient with typical keratoses (case 93).

In other cases after use of arsenic extracutaneous cancers occurred without keratoses or other signs of chronic arsenicism ; so in the cases of Milian (cancer of the breast, case 70), and Semon (1945) (case 142, cancer of the bronchi). The possibility of a mere coincidence of ingestion of arsenic and development of cancer is here of course much greater.

The case of Rosen (discussion to Montgomery, 1935, case 108) concerning epithelioma of the mouth after anti-syphilitic treatment with arsphenamines is already mentioned.

8. *Combination of types.*

Frequently all epitheliomatous lesions in a patient belong to the same type of arsenical cancer, e.g. several growths of the Hutchinson type. But in many other cases two or more different types are associated, as keratoseogen cancer with multiple superficial epitheliomas or with Bowen's disease. In some cases the keratotic cancer develops earlier (as in cases 103 and 128) ; sometimes the superficial epitheliomatosis (case 113). Lesions of the same type in a patient may develop in a different manner, e.g. one superficial lesion as a basal-cell epithelioma and the other as a squamous one. Further, there may arise additional cancers in other organs than the skin. So not seldom a very polymorphous picture is produced not seen in other kinds of growth ; as in the case of Guggenheim (1933, case 78), one cancroid, one basal-cell carcinoma and multiple atypical lesions with Bowen-like changes were combined with verruca seborrhoeica ; another example is the case of Montgomery (1935, case 103, Table I).

9. *Frequency of the different types of medicinal arsenical cancer.*

It is impossible to group every one of all the 143 cases of Table I in the different types mentioned above as the data are not always sufficient ; and besides, there are a number of cases presented in dermatological societies in which the experts did not agree in the special diagnosis. But in about 136 incidences a classification seems possible.

The total number of cases of the keratoseogen type may be assumed as 69.

Fifty-one cases belong apparently to the simple uncombined " Hutchinson type " ; in 36 of them the origin of the malignancy on a keratosis is definitely noted (cases 1, 2, 3, 4, 6, 8, 10, 12, 13, 14, 15, 16, 17, 18, 20, 21, 25, 26, 27, 29, 34, 36, 37, 42, 47, 57, 59, 60, 84, 87, 90, 92, 102, 112, 130 and 143) ; in the remaining 15 cases it can be assumed (cases 9, 19, 35, 40, 41, 46, 51, 56, 58, 62, 64, 71, 91, 94 and 126).

In 3 cases the Hutchinson type was associated with extra-cutaneous cancer (5, 34 and 88).

In 13 cases there was a combination with multiple superficial epitheliomas or with Bowen's disease (cases 11, 23, 49, 85, 104, 106, 107, 109, 111, 113, 116, 127 and 128) ; in 2 cases with multiple superficial epitheliomas and with extra-cutaneous cancer (103 and 123). 67 cases may be counted as multiple superficial epitheliomas including Bowen's disease: 50 of them were uncombined cases (28, 30, 44, 45, 50, 52, 55, 64, 65, 66, 67, 68, 72, 73, 74, 75, 77, 78, 79, 80, 81, 83, 86, 89, 95, 96, 97, 98, 99, 100, 101, 110, 115, 117, 118, 120, 121, 124, 125, 129, 131, 132, 133, 134, 135, 136, 137, 139, 140 and 141); in 13 cases they were associated with the Hutchinson type (see above); in 1 case with extracutaneous cancer (case 75); in 2 cases with the Hutchinson type and with extracutaneous cancer (see above); in another case one of the multiple lesions had arisen in a patch of psoriasis and was associated with cancer of the oesophagus (see below). At the utmost five of these 67 incidences can be classified as cases of Bowen's disease.

Origin of growth in a patch of psoriasis is noted in 7 cases (7, 23, 32, 39, 43, 46 and 138); in case 138 it was multiple superficial epitheliomatosis originating in a patch of psoriasis; later a cancer of the oesophagus developed.

Special cases of growths of the skin are: one incidence of fibrosarcoma (case 53) and one of a melanosarcoma (?) followed by an epithelioma of the cheek (case 69).

The number of cases of extracutaneous arsenical cancer is 13. In 6 of these the skin was not affected (cases 22, 70, 93, 108, 122 and 142); in 7 there were also lesions of the skin (see above).

It is hardly worth while to calculate the percentages of the various types, as they depend also upon the interest the different forms have found at different times. Thus before 1931, of 39 incidences of the then modern keratoseogen type, only 7 of the then nearly overlooked multiple superficial type, Bowen's disease and their combinations with the keratoseogen type were reported. Since 1931 the interest switched over to the latter forms and the ratio of the published cases changed to 15 : 57. Of all cases of epithelioma arising from a psoriatic lesion all but one were reported before 1925.

10. *Multiplicity of medicinal arsenical cancer.*

Hutchinson himself emphasized that in arsenical cancer frequently several (primary) growths are present. Pye-Smith (1913*b*) in his collection of 29 cases found multiplicity of the lesions in fully half of all incidences. In Kennaway's table (1925) there are 75 tumours in 38 patients, giving an average of nearly two tumours in each patient, and besides, two patients had numerous lesions.

Now that many cases of the multiple superficial type have been published, the multiplicity appears to be even more pronounced. The calculation of the cases in Table I shows:

| | Plain keratoseogen cases. | Multiple superf. and Bowen cases, plain and combined with other types. | All cases. |
|---|---|---|---|
| One lesion | 24 = 44·4% | 3 = 4·5% | 41 = 28·7% |
| Two lesions | 10 = 18·5% | 3 = 4·5% | 17 = 11·9% |
| Three lesions | 6 = 11·1% | 6 = 9·0% | 12 = 8·4% |
| Several (4–6) lesions | 13 = 24·1% | 23 = 34·3% | 38 = 26·6% |
| Numerous (10 or more) lesions | 1 = 1·9% | 32 = 47·8% | 35 = 24·6% |
| Sum | 54 = 100·0% | 67 = 100·0% | 143 = 100·2% |

It must be kept in mind that multiplicity of lesions is quite frequent also in non-arsenical cutaneous epithelioma. In a review of 3000 cases of malignant tumours Owen (1921) found 143 (4·7 per cent) of multiple primary growths and not less than 113 of them involved the skin only. And Schreiner and Wehr (1934) report that the most common primary multiple tumour is the basal cell epithelioma of the skin, the squamous-cell epithelioma of the skin being the next in frequency. Hueper (1942) estimates that multiplicity in non-arsenical cutaneous cancer is only 5 per cent.

It is evident that in the simple keratoseogen cases the average number of growths is slightly over two ; whereas in the multiple superficial cases with their various combinations more than one-third shows more than one lesion and more than one-half " numerous " or " several " lesions.

11. *Distribution of medicinal arsenical cancer.*

Hutchinson (1887 and 1888) laid stress on the frequency of location of the malignancies on the hands and feet. Wile (1912) stated a predilection for sites of frequent physiological or other trauma, in contrast to other cutaneous epitheliomas in which the face is the most common seat of disease. Following McCoy (1920) 37·7 per cent of all cancers of the skin occur on the face, hands and neck, all exposed to solar light. Kennaway in 1925 gave a review of the distribution in 38 cases published until this time, mostly of medicinal origin, including the cases collected in 1913 by Pye-Smith. He confirmed the " well-known tendency of arsenic to affect the fingers and toes " in contrast to cancers in tar, pitch and shale oil workers, showing an outspoken predilection for scrotum, head and neck. Among 75 arsenical growths in 38 patients :

46 per cent were located on fingers, leg, foot, toes and trunk.
23 per cent on arm and hand.
23 per cent on scrotum, penis, head, face, neck, eye and eyelid.
8 per cent on other parts.

Kennaway criticized the assertion of Bayet and Slosse (1919*a*, *b*) that the scrotum and its neighbourhood are sites of predilection for arsenical cancer ; this opinion was based on statistics, omitting the so frequent localization on the hands.

Haagensen (1931), comparing the distribution of therapeutic arsenical cancer with occupational cancer, reported 4 new cases of the former with multiple lesions of the hands, arms, legs and trunk, but sparing the face and the scrotum (cases 62, 63, 64 and 65).

Since then many cases of multiple superficial epitheliomas have been added—partly pure cases, partly combined with other forms ; these cases differ in their distribution essentially from those in the Hutchinson keratoseogen type. Therefore in Table II the cases of Table I are divided into several groups.

Table II confirms the preference of the keratoseogen Hutchinson type for the distal parts of the extremities, as nearly 85 per cent of the cases show one or more localizations on fingers, hands, toes and feet, and relatively few on other regions. In contrast more than 92 per cent of the multiple superficial cases presented lesions on the trunk (the cases with distribution " all over the body " included), and frequently also on the proximal parts of the extremities and in more than one-third of the incidences on head and neck. The combined cases show a combination of both sites of predilection. Localization in the genito-anal region is relatively uncommon in both types. In the rare cases of epitheliomas originating from patches of psoriasis nearly always the more proximal parts of the extremities and the genito-anal region were affected.

12. *Chronological course of medicinal arsenical cancer.**

The time elapsing from starting the arsenical drug until the beginning of epitheliomatous growth lasts regularly many years but varies within wide limits : 3 years in case 130 and 40 years in case 74, average being 18·1 years. Even after the cessation of ingestion of arsenic there may be an interval of many years, as 18 years in case 15. There is no marked difference in the duration of this " latent period " between the different forms : in the Hutchinson type the average duration is 18·8 years, in multiple superficial and Bowen's type 17·8 years, in the combined forms 19·7 years.

In the cases with keratoses, especially in the Hutchinson type and in the combined type cases, this latent period may be divided into two stages, the first being the interval between starting with arsenical drugs and the first appearance of the characteristic keratoses ; this prekeratotic stage, noted in 19 cases, lasted from one year† (in case 138) to 20 years (in case 104), with an average of 8·8 years (see above). The second stage is the time between appearance of the keratosis and the onset of epitheliomatous growth ; this keratotic or precancerous stage, noted in only 11 cases, varies from one year (cases 4 and 29) up to 23 years (case 35) with an average of 9·6 years.

13. *Age at onset of medicinal arsenical cancer.*

In spite of the usual long duration of the prodromal period the age when the epitheliomas appear is relatively low—lower than in the common non-arsenical cutaneous cancer. This starts only seldom before the fortieth year of life, the average age being about 60 years (according to Pack and LeFevre (1930), 58 years for the squamous type and 61 years for the basal cell type). The earlier development of arsenical cancer already recognized by Hutchinson (1887 and 1888) was confirmed by all authors. Pye-Smith (1913*a*, *b*) stated that in one-quarter of the cases the patients did not exceed 35 years.

In calculating the age at onset of neoplastic growth in the cases of Table I again the difficulty arises that in a great number of cases accurate dates are missing. Among 115 cases (80 males and 35 females) the age of which is noted, the start of neoplastic growth can be fixed approximately (see Table III). About one-third of all cases were not older than 40 years, and more than 60 per cent not older than 50 years. Remarkable is the considerable number of females between 20 and 30 years.

In one case (" case 2 " of Oliver and Finnerud (1928), not included in Table I on account of the absence of other details) the cancer appeared in a boy of 11 years.

* Duration of exposure to arsenic, see above.

† Appel (discussion to Lane, 1939) reports that in one of his cases arsenical keratoses developed after only 3 weeks' use of arsenic, and Montgomery (1935) has seen keratoses one month after starting the drug.

### *IX. Histology of Arsenical Cancer.*

Both main types of skin cancer are observed in arsenical malignancy :

1. The squamous-cell epithelioma = prickle cell ep. = spinocellular ep. = pearl forming ep. = epidermoid carcinoma, differentiating into all the Malpighian layers; it is marked by a tendency to cornification ("horny pearls" or "cell nests") and a higher degree of malignancy (metastases in lymph-nodes); it is the usual form in arsenical epitheliomas arising on keratoses.
2. The basal cell epithelioma of Krompecher = basilioma = rodent ulcer type, starting from the basal layer of the epidermis, with no tendency to cornify and comparatively benign course (no tendency to metastases), although it consists of younger and less differentiated cells than the squamous type. It is the most frequent form in multiple superficial epitheliomas.

There are also mixed forms and transitions from the basal-celled to the squamous-celled type. Besides, both types of epithelioma may occur in the same patient.

For the histology of Bowen's disease and extramammillary Paget's disease see above.

Among 81 cases in Table I with cutaneous tumours in which the result of histological examination is noted, at least 44 were of the squamous type and at least 24 were basal-celled; one was a mixed form. In 10 cases squamous-cell tumours as well as basal-cell tumours were present. Only in one case (129) the growths were microscopically exactly identified as Bowen's disease. In one case (case 53) a fibrosarcoma and in one case (case 69) a melanotic sarcoma is noted.

### *X. Chemistry and Histochemistry in Arsenical Cancer.*

Arsenic after ingestion for a long time is said to be deposited in the organs, especially in the skin and its appendages (sweat glands, sebaceous glands, hair follicles, hair and nails). Partly it is excreted—very slowly—by these glands (see Cornbleeth, discussion to Montgomery and Waisman, 1941) and in the urine. For chemical investigation in the living are available : urine, blood, spinal fluid, hair, nails and excised samples of the normal and pathological skin.

The methods of chemical examination are based on the classic method of Marsh. Now mostly used is the electrolytic technique of Gutzeit (1879) or of Osterberg (1928). Table IV gives the results of the authors in cases of arsenical epitheliomas in the urine.

The value of such investigations of the urine for the diagnosis and for the clearing up of the pathogenesis is by no means as high as should be expected. It must be kept in mind that arsenic in small amounts is apparently a normal constituent of the body, depending on environmental conditions, as water, food and occupation (Heffter and Keeser, 1927; Rost, 1931).

Thomsen (1905 *cit.* Schwarz, 1932) was the first to detect arsenic in the normal urine. Billeter and Marfurt (1923) found 0·00056 mg. per cent and 0·0104 mg. in the daily quantity; Althausen and Gunther (1929) 0·08 and 0·16 mg. per cent; Throne and Myers (1935) 0·00104 mg. per cent; they think an amount of over

0·03 mg. per cent of dried substance to be pathological. Wuehrer (1937) found 0·005–0·095 mg. per cent in normal urine. In the urine of (healthy) workers with arsenicals Schwarz (1932) detected amounts up to 0·82, and Wuehrer in cases of chronic arsenicism (without epitheliomas) 0·245–1·110 mg. per cent $As_2O_3$. So practically all quantities found in the urine of patients bearing arsenical epitheliomas (Table IV) are within the limits sometimes contained in the urine of apparently normal individuals.

It may be mentioned that after ingestion of arsenic the urine contains arsenic partly as an organic compound (Salkowski, 1908).

Some authors report determinations of arsenic in *blood* (Cannon, discussion to Montgomery 1935, and Montgomery, 1942) but no exact quantities are given in cases of arsenical cancer.

Still less successful than the examination of the urine proved the investigation of *hair* and *nails* in cases of arsenical growths. Caspar (1860) first described the presence of arsenic in the hair of a patient in whom arsenical poisoning was suspected. Since then many determinations in the hair were made (Schwarz, 1932; Tost, 1931; and Wuehrer, 1937). The values in the hair of normal persons lie between 0 and 0·18 mg. per cent (Myers and Cornwall, 1925; Billeter and Marfurt, 1923; Althausen and Gunther, 1929; Wuehrer, 1937), whereas after ingestion of arsenic and in (healthy) workers with arsenical insecticides amounts of 0·245, 1·110, 5·0 and even 160 mg. per cent (Schwarz and Deckert, 1943) were reported. It seems strange that investigation in three cases of arsenical cancer (Montgomery, 1935, cases 96, 97 and 98 of Table I) did not reveal any arsenic in the hair.

In the nails where normal amounts of 0·0172 mg. per cent were stated by Billeter and Marfurt (1923), Hamilton (1921, case 36) found only a trace.

In normal *skin* Billeter and Marfurt (1923) detected 0·0097 mg. per cent, and Oppenheim (1931) in the scales of normal skin 0·0026 mg. per cent. The examination of the keratoses, growths and normal skin in cases of arsenical cancer has given repeatedly negative results; as in the incidences 79, 101, 102 and 120 of Table I; in others amounts up to 8·6 mg. per cent (case 104) and even 430 mg. per cent (case 127) were recorded (Table V).

The results of chemical determination are disappointing on the whole. The main cause may be that—except for urine—usually only very small samples of tissue are available. It seems that the methods of determining arsenic in such small quantities are still unreliable. For this reason the histo-chemical method is preferred by the majority of authors. This "hydrogen sulphide method" has been worked out by Justus (1905), following a proposal of Carlson; the arsenic of the tissues is transformed to arsenic trisulphide $As_2S_3$, which can be recognized microscopically as yellow-brown to greenish-yellow crystals of high refractivity, nearly always of uniform size. The original technique has been modified by Bruenauer (1921) and Osborne (1925, 1928 and 1932). Osborne's technique is now mostly used. As a microscopical method of course it cannot give real quantitative values, but it is very sensitive, requires a minimal quantity of material and permits a location of the arsenical deposit. The method is supported by a series of authors: Montgomery (1935), Montgomery and Waisman (1941), and O'Leary (discussion to Waugh and Scull, 1935). But it has not escaped criticism. Muir and Tannenholz (1933) objected, stating that the crystals were in reality sulphides of albumin. Other authors are also critical concerning the proof of arsenic by the histo-chemical method: Oppenheim and Fantl (1934), Becker (discussion to Waugh and Scull, 1935), and Nomland (*ibid.*). In any case the method requires fully experienced qualified investigators.

Bruenauer and Osborne obtained always negative results in the skin of normal persons and positive in arsenical keratoses.

Anderson (1932, case 69) and Goeckerman and Wilhelm (1940, case 114) reported positive results with Osborne's method. But the most extensive use of examining normal and pathological skin of the patients by chemical and histochemical methods was made by Montgomery (1935, 1941) (Table V). Montgomery himself emphasizes that arsenic in pathological amounts can be found by Osterberg's method in cases of various types of dermatoses without there being any clinical evidence of arsenic, as keratoses or pigmentation. On the other hand, neither he nor Osborne was able to demonstrate arsenic in the skin of certain patients who had got arsenic for some time, even in some cases with arsenical keratoses or typical arsenical cancer. So the mere demonstration of arsenic by chemical or histochemical methods does not prove that the drug is an etiological factor in a given case; and its absence does not exclude arsenic as a causative factor. O'Leary (discussion to Waugh and Scull, 1935) has confirmed these observations.

Cannon (discussion to Montgomery, 1935) may be right in saying that it is necessary to learn more about the significance of arsenic in normal persons.

Guthmann (1938) reported that he had found in the venous blood of normal women 0·01–0·06 mg. per cent, with considerable variations corresponding to the menstrual cycle, and still higher amounts up to 0·24 mg. per cent in pregnancy. In cancers of different origins he found quantities of 6–7 mg. per cent, together with an increase in the venous blood up to 4½ times the normal value. He suggests a close general connection of arsenic with different proliferation of cells. But his values are so astonishing and in such apparent contradiction to the results of other authors that confirmation would be necessary (see p. 229, Pathogenesis).

### *XI. Prognosis of Arsenical Cancer.*

As with cutaneous cancers in general, arsenical epitheliomas of the skin are slowly growing neoplasms with a relatively low grade of malignancy, the microscopically basal-celled forms giving a better prognosis than the squamous ones. Besides, there are obvious differences between the different clinical types.

The keratoseogen cancers of the Hutchinson type—nearly always squamous forms—although often growing very tardily at first, give the most unfavourable ultimate prospect; especially the cases originating on the fingers. They lead eventually to metastases in the regional nodes and in the internal organs, to emaciation and death; the multiplicity of lesions is another aggravating factor. Such tumours localized on other sites seem to have a somewhat less unfavourable course.

Much better is the prognosis in superficial epitheliomas and in Bowen's disease; but there are also differences. The most favourable are the cases of true superficial "benign" epitheliomatosis showing the histological picture of basal-celled epitheliomas, and often without any sign of keratosis or pigmentation. They cannot be called real cancers, but occasionally they become malignant (e.g. case 139, discussion to Glen, 1945), or they are associated with other malignant forms of epithelioma. Less favourable are the cases of superficial epitheliomas with marked keratoses (Montgomery's " epithelioma of the arsenical type "), frequently showing the histological picture of squamous-cell epithelioma ; they are usually not so malignant as the keratoseogen forms, and Montgomery and Waisman (1941) suggest that the clinical course is frequently not so bad as the histological picture would suggest, but nevertheless they often lead to invasive growth, metastases and death.

In Bowen's disease (see p. 205) the lesions may be constant for many years, although the microscopical picture is like squamous cancer. Approximately 20 per cent eventually develop into a penetrating form of cancer.

Death is noted in 17 cases of Table I, but no doubt the number of fatal cases is in reality far greater.

### *XII. Treatment of Medicinal Arsenical Cancer.*

There is general agreement that the main task in therapy is prophylaxis. That means, in medicinal arsenical cancer, to confine the use of this powerful drug to the cases of necessity. Arsenic seems to be dispensable as a tonic and as a preventive for bromide eruption in epilepsy, etc. (its efficiency for that is even doubtful, see Wigley, discussion to Russell and Klaber, 1945). That applies especially to the treatment of psoriasis, as patients suffering from this disease are apparently more susceptible to the different kinds of arsenical epithelioma. The question was specially discussed in connection with Anderson's paper (1937). Some authors (Ayres, discussion to Anderson, 1937 ; Netherton, discussion to Barney, 1944) suggest that the benefit obtained by arsenic in psoriasis does not justify the risk of administering it, or only in exceptional cases. Caskey (discussion to Anderson, 1937) declared he never mentions arsenic to his students or physicians without condemning it. Others (e.g. Jacobsen, discussion to Anderson, 1937) do not go so far ; for arsenic is doubtless very effective in many cases of psoriasis—testified by the very histories of some patients of Table I (see cases 1, 23, 24, 38, 43, 120 and 130) ; and it must be considered that in comparison with the high frequency of psoriasis and the general use of arsenic for its treatment the development of arsenical cancer is quite a rare event. In the therapy of lichen planus and of dermatitis herpetiformis also arsenic cannot be spared easily. Halloran (discussion to Anderson, 1937) declared it would be a mistake to develop a fear complex as to the use of arsenic as a drug. But certainly there should be legislation to prevent or curtail the general use of arsenic in patent medicines, and it should not be permitted that a patient can get the drug continuously without a new prescription. Repeatedly it was claimed in the discussions that the prescription must have the remark, " Not to be refilled." It is equally necessary to instruct students about the danger of arsenical medication, and the indispensability of a regular control of patients treated with arsenic. The use of arsenical paste for the treatment of cancer should be abandoned, even although it has proved in some cases to be successful (see picture in Sutton, 1939, p. 729).

Ullmann and Galloway (discussion to Pye-Smith, 1913*a*) sounded a note of warning that the use of arsphenamine in large quantities might also be followed by malignant epithelial lesions. But experience has shown that there is not sufficient reason to restrict such a valuable drug in the treatment of syphilis, as the development of malignancy after its use is very exceptional (see p. 196), and such patients are as a rule under medical supervision. Only in cases of syphilitic affection of the mouth, especially leucoplakia, a certain reserve may be recommended (Rosen, discussion to Montgomery, 1935).

As soon as signs of chronic arsenicism (pigmentation, keratosis, etc.) begin to appear, the medication with arsenic has to be stopped at once. The use of sodium thiosulphate was recommended : 5–10 c.c. of freshly prepared 10 per cent solution intravenously every third day or orally 0·5 g. in enterically coated tablets. Ravaut (1920) and Dennie and McBride (1924) have introduced this treatment. It is supposed that this salt converts arsenic in the body into the insoluble harmless sulphide (see Throne and Myers, 1935). Some authors (Ayres and Anderson, 1938) believe that it increases the excretion of the poison. Many authors support this treatment : Althausen and Gunther, 1929 ; Throne and Myers, 1935 ; Greenwood (discussion to Lane, 1939). But new investigations have shaken its theoretical basis (Editorial, 1942 ; Abramowitz, Mattice and Bottvinck, 1944). Appel (discussion to Lane, 1939) recommends the external local use of sodium thiosulphate in the form of fairly concentrated soaks. Also, soothing ointments (hydrous wool fat and petrolatum) or keratolytic ointments (with salicylic acid) may be applied for removing the keratoses. Franseen and Taylor (1934) recommend prophylactic destruction of the keratoses already in the pre-cancerous period. But it must be kept in mind that any irritation, mechanical or chemical, must be avoided (see p. 229, Pathogenesis). Of course regular medical supervision is necessary.

If a case of keratosis is developing signs of malignancy, radical measures have to be taken at once ; they consist either of surgical removal (excision), or destruction by one or the other forms of cautery. In cancerous lesions of the fingers, and in extensive lesions of other parts of the extremities, amputation is advisable. It has been recommended in all cases of squamous cancer to remove the regional nodes too (Franseen and Taylor, 1934).

The more conservative methods of treatment such as radiotherapy (X-rays, radium) have proved insufficient, and even contra-indicated in keratoseogen arsenical lesions ; recurrences were observed very often (Montgomery and Waisman, 1941). Some authors recommend radiotherapy as an after-treatment after surgical removal ; but others (Galloway, discussion to Pye-Smith, 1913*a*) think that radiotherapy should be avoided entirely in the treatment of arsenical cancer.

For the treatment of multiple superficial epitheliomas and Bowen's disease it was thought that less radical procedures are justified, such as the use of X-rays, radium, ultraviolet irradiation (Ayres, 1934), or " Grenzstrahlen " (Kindler, discussion to Glen, 1945). But already Graham Little (1923) had noted the marked radio-resistance of these lesions, and other authors have confirmed this observation (Lane, discussion to Ormsby and Mitchell, 1925 ; Anderson, 1932 ; Glen, 1945 ; Dowling, discussion to Glen, 1945). Graham Little recommended freezing with carbon dioxide snow, already proposed by Galloway (discussion to Pye-Smith, 1913*a*) ; Schamberg (discussion to Pfahler, 1927) and Crawford (*ibid.*), electro-desiccation ; Rodgers (1941) used cauterization with silver nitrate ; Pusey (discussion to Ormsby and Mitchell, 1925), painting with nitrate of mercury (Weber, *ibid.*). But most of the experts now advise complete excision also for this kind of epithelioma, if necessary with skin grafting (Lane, discussion to Ormsby and Mitchell, 1925 ; Michelson, discussion to Fraser, 1928 ; Montgomery, 1929 ; Glen, 1945 ; Dowling, discussion to Glen, 1945). Of course there may be difficulties in cases with numerous lesions. Ayres (1934) recommended after-treatment with sodium thiosulphate against further development of epitheliomas.

Of course, periodical re-examination of the skin is essential.

### B. ARSENICAL CANCER CAUSED BY DRINKING-WATER.

As early as in 1809 Lambe (Eggers, 1932) expressed the belief that arsenic in potable water may be the cause of malignant disease.

One of the most impressive examples of an endemic occurrence of chronic arsenicism is the so-called *Disease of Reichenstein* (" Reichensteiner Krankheit "). Reichenstein is a small town in the county of Glatz, Silesia, where for many centuries gold was produced from gold-containing arsenical ores : arsenopyrite (= arsenkies, mispickel FeAsS) and lollingite (= arsenikalkies = arseneisen $FeAs_2$ respectively $Fe_2As_3$ and $Fe_2As_5$). With the old methods of smelting large quantities of arsenical fumes escaped, were precipitated with the rain and thus reached the underground water, the brooks and the drinking-water. In one of the brooks (" Giftbach " = poisonous brook) 1·22 mg. per cent arsenic was found ; in samples of the mud up to 500 mg. per cent. Later the gold production was dropped and only arsenic was produced. For centuries the high incidence of serious diseases in that town was known. But not until 1898 was the nature of this disease recognized as chronic arsenic poisoning, and described by Geyer. The main symptoms were : troubles of the gastro-intestinal tract, ulcers in the mouth, perforation of the nasal septum, paraesthesia, and especially melanosis and keratotic warts on fingers and hands of the same appearance as in arsenical poisoning. In some cases cancer developed. Geyer refers to the reports of two local practitioners giving details of three cases of this kind, all with keratoses of the hands and melanosis. It seems that such cases were very frequent in Reichenstein ; for Kathe (1937) was told by another doctor there that half of his patients with " Reichenstein disease " had died with cancer. Unfortunately written records are missing.

The " Reichenstein disease " has disappeared since the methods of smelting have been changed, and particularly since the town was provided with a new and adequate water supply in 1928, with an arsenic content of at the most 0·0015 mg. per cent (Geyer, 1940).

Another occurrence of endemic arsenical intoxication caused by drinking-water is reported from the Argentine, province of Cordoba. In a wide area in the south and east of this province, including the town of Bellville, such cases have been observed by Goyenchea, by Ayerza and Arrillaga and others (Fernandez, 1925 ; Bosco, 1925 ; Roffo and Correa, 1926 ; Alvarez and Ruiz, 1927 ; Alvarez, 1928 ; Garcia and Ruiz, 1928 ; Alvarado, 1930 ; Seminario and Alvarado, 1931 ; Roffo and Rosner, 1935 ; Trelles, 1937 ; Arguello, Cenget and Tello, 1938 ; Arguello, Ferraris and Tello, 1938 ; Arguello, Tello and Marcola, 1942 ; Zinny and Vivaldo, 1942 ; Arguello and Tello, 1943). The drinking-wells of Bellville have been found to contain an abnormally high amount of arsenic, up to 0·28 and 0·45 mg. per cent ; iodine and vanadium are also present in many of these waters. Many cancers of the skin have been observed in Bellville, mostly in peasants. Arguello and co-workers alone give reports of 65 cases. The clinical picture is in accordance with that of cancer caused by arsenical drugs : keratoses of the palms and soles seem to be present in nearly all cases, as in all 39 cases reported by Arguello, Cenget and Tello (1938) ; often melanosis—in 12 per cent of the cases in the mentioned paper. There is also a tendency to multiplicity and a predilection for the limbs, especially hands and feet, and a relative rarity of localization on the head. Among their 39 cases Arguello and his co-workers found multiplicity in 33·3 per cent ; localization on the limbs in 38 per cent ; on the trunk in 13 per cent ; on the head in only 15 per cent ; in no case on the genitals. The corresponding percentages in non-arsenical cutaneous cancer was found by the same authors to be : multiplicity in 5 per cent, site on the limbs in 4 per cent, on the trunk in 2·5 per cent, on the head in 81·5 per cent, and on the genitalia in 5 per cent.

The majority of the epitheliomas were of the squamous cell type (51 per cent) ; less frequently of the basal cell type (7·6 per cent). In a second paper Arguello, Ferraris and Tello (1938) report 26 new incidences, including three cases of Bowen's disease supporting Anderson's view that Bowen's disease is a form of arsenical intoxication (see p. 205).

There are several wells in Europe with a very high content of arsenic, frequently used for therapeutical purpose, as :

| | |
|---|---|
| Guberquelle (Bosnie) . . . | 0·6 mg. per cent arsenic. |
| Levico (Tyrol) . . . . . | 0·6 ,, ,, ,, |
| Duerkheim (Palatinate) . . . | 1·74 ,, ,, ,, |
| Roncegno (Tyrol) . . . . | 4·26 ,, ,, ,, |

for comparison :

| | |
|---|---|
| Bellville in the Argentine . . . | 0·28–0·45 mg. per cent arsenic. |
| "Giftbach," Reichenstein . . . | 1·22 mg. per cent arsenic. |

In the Report of the Royal Commission on Arsenical Poisoning (1903) the limit of arsenic for liquid food (and water) was set up as one-hundredth of a grain of $As_2O_2$ per gallon = 0·015 mg. per cent. Nothing is known of a more frequent occurrence of cancer in these localities either at the present time or in the past.

Summarizing, it can be stated that cancer caused by the arsenic content of drinking water is apparently identical with medicinal arsenical cancer. In Cordoba not only the keratoseogen type but also the multiple superficial type and Bowen's disease were observed ; that they are not mentioned in Reichenstein is not astonishing, as at that time these affections were not known. It seems that in Reichenstein melanosis was very frequent, and more pronounced than usual in the medicinal cases ; besides, perforation of the nasal septum is mentioned as a frequent sign, not hitherto observed after ingestion of arsenic as a drug. But these are minor differences.

### C. OCCUPATIONAL ARSENICAL CANCER.

#### *I. Arsenical Cancer in Miners and Smelters.*

The first hint that arsenic may be the cause of development of cancer in man is to be found in the *Pharmacologia* of Dr. J. A. Ayrton Paris. Having practised in Penzance (Cornwall) from 1813–1817, he asserts that the smelting works developing arsenical fumes in Cornwall and Wales occasionally cause in the workers cancerous disease of the scrotum similar to that which affects chimney sweeps. This remark of Paris, quoted over and over again, was criticized by Butlin and by Kennaway.

Butlin (1892), making careful inquiries of the doctors in Camborne and Hayle, failed to find the smallest evidence confirming the assertions of Paris; the doctors practising there had never seen a case of scrotal cancer. A later inquiry by Hulke (discussion to Lane, 1894) amongst the workers in the Cornwall mines as to the occurrence of cancers also had a negative result. These differences could perhaps be explained by the fact that the method of smelting since Paris' time had changed, producing now mainly tin and developing less arsenic fumes. But that would not meet the objection pointed out by Kennaway (1943) that the arsenical cancer prefers other locations, affecting the scrotum very seldom. Out of 75 cases of arsenical cancer reported in the literature only 4 were localized there.

The question has been discussed as to whether the so-called "*Schneeberger Lungenkrebs*" and the pulmonary cancers observed amongst the miners of *Joachimsthal* are due to arsenic. For centuries the high mortality rate amongst the miners in Schneeberg and in other districts of the Saxonian Erzgebirge (ore mountains), as Annaberg, etc., has been known. It was mentioned by Agricola (1556) and in chronicles as due to "Bergkrankheit" (mountain disease). But not until 1879 was the condition described by Haerting and Hesse as cancer of the lungs. Arnstein (1913*a*, *b*) stated that they are real carcinomas, not lymphosarcomas or endotheliomas, as was suggested by Wagner and Weigert. Haerting and Hesse stated that 75 per cent of all deaths of the miners was due to this affection—later statistics show 40–70 per cent (Arnstein, 1913*a*, *b*; Lange, 1935). The onset of the disease in this district occurred as a rule after 20 years of working in the mines.

As other persons in this district were affected very rarely, this kind of cancer must be in some way connected with the mining. Haerting and Hesse assumed the cause of the disease to be irritation caused by inhaled arsenic with other respiratory affections and a bad state of nutrition as predisposing factors. The main ores in Schneeberg—besides sulphidic ones—are arsenides, as "Speiskobalt" (tin white cobalt $CoAs_2$), "Weissnickelkies" (white nickel $NiAs_2$) and "Rotnickelkies" (arsenical nickel NiAs); these compounds are more soluble than the compounds of arsenic and sulphur present in other cobalt pits. The bore-dust of the mines contains up to 0·45 per cent As (Rostoski, Saupe and Schmorl, 1926), the dust in the pits 0·1 per cent. For the importance of arsenic in the etiology of the Schneeberg cancer Saupe (1930) laid stress on the experimental production of pulmonary malignancy by Fischer-Wasels (1929, see p. 228) and Schmorl's (*cit.* Saupe, 1930) finding of two cases of lung cancer in the necropsies of workers in the forges of Freiberg-Muldenhuetten, where now arsenic is produced from different ores. But Saupe himself (1930) was unable to detect more instances of lung cancer in Freiberg, although sometimes signs of chronic arsenicism were found. It may be remembered that—why, is not known—in the last ten years cancer of the lungs, formerly rare, has become a quite frequent disease.

Considering all facts arsenic can hardly be said to have an important etiological role in the Schneeberg cancer. No other signs of arsenicism are reported in these patients, especially not keratoses. The localization of cancer in the lungs is extremely rare in typical arsenical cancer: out of 143 medicinal arsenical epitheliomas of Table I the lungs and the bronchi were affected only in two cases (103 and 142). On the other hand, in the Schneeberg cases, although eczematous changes occurred frequently, never a cancer of the skin has been reported. Multiplicity of the tumours, so frequent in true arsenical cancer, is usually missing in the Schneeberg malignancies (Hueck, 1939/40). The tests for arsenic in urine, hair and nails gave negative or doubtful results (Rostoski, Saupe and Schmorl, 1926), and so did the examination of the lungs (Pirchan and Šickl, 1932, in Joachimsthal miners, and Ziel, 1935). Besides, pulmonary cancer is not very frequent in other parts of the world where arsenic is mined.

Other factors blamed for causing the Schneeberg cancer are: pneumokoniosis, chronic irritation by respiratory diseases, cobalt and hereditary susceptibility.

Now the general view is that the main etiological factor is the presence in the pits of radioactive substances, the carcinogenic action of which is well known (Ludewig and Lorenser, 1924). Also it was stated that in Joachimsthal, on the southern side of the Erzgebirge, famous for its content of pitchblende and radium, lung cancer is also found (Loewy, 1929; Šikl, 1930). In the necropsies of the miners there between 1928 and 1938 summarized by Peller (1939), in about 50 per cent lung cancer was found. In contrast to that, in the mines of Southern Sweden containing Speiskobalt, as in the Schneeberg mines, pulmonary cancer is unknown.

Recently Lorenz (1944) arrived at the opinion that radioactivity cannot be the sole cause of the pulmonary cancer of the miners, because similar doses of X- or $\gamma$-rays do not produce lung tumours in animals. He suggests some contributory factors, including arsenic.

Although there is no basis to suppose that arsenical cancer is widespread in miners and smelters, that does not deny that such cases occur occasionally. Some cases are reported in the literature: the case of Anderson (1932, case 1 of Table VI), concerning a man of 51 who as a boy had worked in a smelting works in Mexico, and developed warty keratoses of palms and soles and multiple benign superficial basal-celled epitheliomas; in the normal parts of the skin arsenic was detected, and still more in the lesions. The patient knew the lesions were due to arsenic. He stated that "everybody in the Mexican mines has the same thing."

Another case was reported by Migayi (1935): after this man had worked for ten years in a copper smeltery in Japan in which arsenic was produced, keratotic changes appeared on the soles and palms, pigmentation of the skin, and some years later a cancerous ulcer (squamous) developed on a keratosis.

A third case is that of Schaerrer (1934)—a worker mining or smelting silver ores.

A fourth case was mentioned by Andrews (discussion to Goeckerman and Wilhelm, 1940)—a miner in a copper mine, with typical keratoses and epitheliomas.

Also Bayet (1930) cites Rayer, who reported some cases of arsenical cancer among workmen employed in the handling of arsenical ores.

### *II. Arsenical Cancer in Factories.*

1. *Sheep dip factories.*

Legge (1902) described, from a sheep dip factory in England, incidences of irritation of the upper air passages combined with keratosis of the skin and pigmentation as indicative of arsenic poisoning. Sheep dip is a compound of arsenite of soda and free arsenious acid used for the treatment of mange in sheep. The product is very dusty. Eve stated in 1910 that all workers at the factory were deeply pigmented (O'Donovan, 1928).

Pye-Smith (1913*b*) in his table of 31 cases of arsenical cancer recorded two until then unpublished incidences of cutaneous cancer in persons employed for years in such a factory, observed by Eve and by Porter. These observations were verified by later experience. Legge (1923, 1924) reported four similar cases. O'Donovan, describing again Porter's and one of Legge's cases, gave a review of the facts. Bridge (1926, 1930) mentioned three further incidences. Legge (1934), repeating apparently three of the former observations, reported another doubtful case. Merewether (1943) detected the incidence of fatal pulmonary cancer in a sheep dip worker, and remarked that 3 similar cases occurring in sheep dip workers had been notified since 1939. Currie (1947) found columnar-celled adenocarcinoma in the lung and bronchial lymph glands in case 15, Table VI. It is not noted whether these patients have shown any signs of chronic arsenicism, especially keratoses. So this matter needs further examination. This amounts to 13 cases of arsenical cancer in sheep dip workers all observed in England (Table VI). Arsenicism in sheep dip workers has become rare since the plants were remodelled to render them as nearly dust-free as possible.

2. *Cancer caused by arsenical insecticides.*

Chronic arsenicism has been observed in workers employed in the manufacture of arsenical insecticides. There are a great number of different preparations, with trade-marks such as Emeraldgreen, Silesiagreen, Titaniagreen, Urbangreen, Aresin, Esturmit, Vinuran, Grelit, Meritol, Nosprason, etc. Most of them contain as the main compound aceto-acetate—arsenite of copper = Schweinfurt-green $Cu(C_2H_3O_2) + 3Cu(AsO_2)_2$; others, arsenite of copper; Scheele's green $CuHAsO_3$; others calcium arsenate or lead arsenate. Schwartz and Deckert (1943) found very large amounts of arsenic in the hair of these workers, 74 to 160 mg. per cent (see p. 212).

The arsenicism in these workers may lead to the development of cancer; two incidences were published, one by Legge (1925) and by Bridge (1928), another by Bridge (1939); the latter with a pulmonary cancer besides skin manifestations (see Table VI).

These arsenical insecticides are especially used by gardeners, fruit farmers and vintagers for combating peronospora. They are used partly in the form of a powder and partly as a spray. It is no wonder that chronic arsenicism has been found also in such workers (Doerle and Ziegler, 1930).* Hyperkeratosis of the

* Aschoff (1887–1899) was the first who brought attention to the high frequency of cutaneous cancer in gardeners; this observation was confirmed by Young and Russell (1926), who stated that in gardeners, nurserymen, seedsmen and domestic gardeners the percentage of death from cancer of the face is 150 per cent in excess of the expected amount, deaths from cancer of the hands even 300 per cent. Exposure to sunlight, contamination with fertilizers (soot !) were held responsible; but handling of arsenical insecticides surely plays also a role.

skin with multiple warts and melanosis are the most conspicuous symptoms (Pein, 1938*a*, *b*). In some of such cases cancer of the skin developed (see Table VI, cases 20–23). All four cases showed keratoses, two melanosis. But none of them presented the true Hutchinson type of skin cancer. The cases 21, 22 and 23 may be grouped as multiple superficial epitheliomas. Case 20 shows an uncommon localization all over the face ; it may belong to the type mentioned by Cannon (discussion to Montgomery, 1935) (see p. 204).

Pein and Baurhenn (1943) believe that the high incidence of arsenicism in wine-dressers in Germany is not really occupational, but is due to the consumption of wine at home (" home-drink "). This may contain 0·1–0·2–0·5 mg. per cent arsenic, i.e. up to 2–12 mg. daily.

3. *Arsenical cancer caused by coal and its by-products.*

Cancer of the scrotum in chimney sweeps is well known since its description by Pott (1775). Volkmann (1875) described occurrence of scrotal cancer in workers with tar, paraffin and soot. One year later Manouvrier in Lyons (Bayet and Slosse, 1919) reported that in the workers with coal and its by-products frequently a syndrome was observed which he supposed to be caused by a poison and which he named " l'intoxication houillière " ; but he was not able to find out the responsible poison.

Pye-Smith in 1913 brought attention to the fact that pit-coal contains always arsenicated iron-pyrites in various proportion, and that its derivatives, such as soot, tar and pitch, contain still more of this poison. He pointed out that the affections of the skin often seen in these workers (keratosis, melanosis, warts and epithelioma) might be explained in that way, including the cancer of chimney-sweeps, much more frequently found in England than on the Continent, where wood coal was more commonly used.

Bayet and Slosse resumed these thoughts in 1919 with much emphasis. They found arsenic not only in pit-coal and its by-products, but also in blood, hair and urine of the majority of the workers engaged in making briquettes from pitch. They suggested that the clinical picture of the " l'intoxication houillière " is identical with that of chronic arsenicism ; melanosis, hyperkeratoses, troubles of the digestive tract, bronchitis, ulceration of the nasal fossa and the occasional occurrence of cutaneous cancer with special features ; appearance at a relatively early age, development on a keratosis (" verrue du brai "), frequent multiplicity, frequent localization on the scrotum and its neighbourhood. Bayet asserted that 70 per cent of the cases of both arsenical cancer and coal cancer had this location. So Bayet and Slosse concluded : " Le cancer arsenical et le cancer du goudron sont identiques."

This view, although accepted by some authors, as La Rossa (1925), has been rejected by both German and English authorities :

1. Some kinds of coal and tar, although free or nearly free of arsenic, cause also cancer in mice (Moeller, 1923 ; Daels, 1923 ; and Ross, *cit.* Bayet, 1930).

2. Bayet and Slosse (1919) themselves mentioned that their theory is unable to explain the fact that the workers handling coal itself (les " houilleurs ") are mostly not affected in contrast to the relatively high incidence in workers employed with the by-products, such as tar, soot, pitch, etc. (Pye-Smith, 1913*b*).

3. The identity of the clinical pictures has been denied. The keratoses of the palms and soles so characteristic of chronic arsenicism are not typical of cancer caused by the by-products of coal. It is true that pigmentation is found in both conditions, but in pitchworkers it affects mostly the bare parts of the body. But the main objection is that the anatomical distribution is essentially different in pitch cancer and in real arsenical cancer, as Kennaway (1925) has pointed out in a special study. In contrast to the well-known predilection of arsenical cancer of fingers and toes, only two out of 209 cancers were situated on the fingers and none on the toes. Nearly one-half of the arsenical cancers but only 4 per cent of the pitch and tar cancers were situated on the fingers, hands, legs and trunk ; on the other hand 83 per cent of the pitch and tar cancers but only 23 per cent of the arsenic cancers occurred on the head, neck and scrotum ; so the special liability of the scrotum as the site of the pitch and tar cancer is not seen in arsenical cancer. The statistics of Bayet and Slosse were distorted by their procedure to exclude *a priori* all localizations on the palms and soles. Besides, the multiplicity of tumours is much more pronounced in the arsenical cases—75 cancers in 38 cases (the two cases of multiple cancers excluded), whereas in 123 persons with tar and pitch cancer only 144 tumours were recorded.

4. Finally, the theory of Bayet and Slosse has lost any basis by the definite identification of the real carcinogenic components of tar and pitch by the work of Kennaway and Cook..

So the theory of Bayet and Slosse has to be abandoned.

4. *Cancer of the bladder in aniline workers.*

Bayet and Slosse (1919), Bayet (1930) and Hamilton (1921) have suggested that the papillomatous and cancerous tumours of the bladder observed repeatedly in workers with aniline dyes may be caused by arsenic. They based this contention on the presence of arsenic as a contaminant in these dyes, the demonstration of arsenic in the urine (Wignall, 1920), and the possible similarity of cutaneous manifestations in these workers with arsenical dermatitis.

But this view was opposed by many authors (Posner, 1904, 1905 and 1924 ; Kennaway, 1925 ; Ullmann, Henry, 1930 ; Berenblum, 1932). Tumours of the urinary tract have been observed in arsenic poisoning but are very rare (see p. 206) ; the dye-workers suffering from cancer of the bladder present no signs of chronic arsenicism. Kennaway has pointed out that it is not clear why arsenic should cause cancer of the bladder in aniline workers, but cutaneous cancer in the general population. However, the aniline dyes are now free of arsenic, so that this poison can no longer be responsible for these bladder growths.

### *III. Comment on Occupational Arsenical Cancer.*

In reviewing the observations in the literature in a critical way, a series of suspected occupational arsenical cancers must be excluded. There is no evidence whatsoever that arsenic is responsible for the pulmonary cancers of Schneeberg and Joachimsthal, for the malignancies in coal and pitch workers, and for the bladder tumours in aniline workers. Only the occurrence in factories of sheep-dip and of arsenical insecticides, in gardeners, farmers and vintagers and a few incidences in miners and smelters (see above) really can be attributed to arsenic. The total number of such cases recorded until now is, as Hueper (1942) mentions, astonishingly small in comparison with the large number of persons exposed to arsenic in industry. It may amount to about 25 cases.*

The clinical picture of these occupational cancers is on the whole the same as in the more frequent and well-known medicinal cases. But there are some special features in the occupational cases (Fassrainer, 1936).

(1) All reported cases concerned males—a not surprising fact.

(2) The period of exposure to arsenicals is very long in most cases, ranging from 4 to 46 years, with an average of about 25 years (Table VI).

(3) Perforation of the nasal septum, a sign never mentioned in the therapeutic cases is very frequent as a concomitant in workers in sheep-dip factories (Legge, 1934), as well as in workers producing arsenical insecticides (Bridge, 1928, 1939). The reason why this symptom has not been observed in the medicinal cases may be found in the different route of exposure, the poison in the occupational cases being inhaled as dust and so irritating the upper air passages. But in the Reichenstein disease caused by drinking arsenic-contaminated water it is mentioned also as a frequent sign (see p. 216). It may be that such perforations occurred in some of the medicinal cases also, but escaped observation as they make no deformity and often no trouble, so that many of the affected are ignorant of this condition (Legge, 1934).

(4) Melanosis of the skin seems to be more frequent and more pronounced in the occupational cases. Whereas pigmentation is mentioned in only 16·4 per cent of the medicinal cases, it was present in at least 9 of 16 cases about which detailed notes are given, i.e. in 56 per cent (see Table VI). Legge (1934) seems to be inclined to ascribe this pigmentation of the skin to the working for years in contact with arsenical dust. But it may be remembered that in endemic arsenical cancer caused by drinking water (Reichenstein, Cordoba), pronounced melanosis is an almost constant sign.

(5) Keratosis, especially keratosis of the palms and soles, seems to be as frequent as in the therapeutic form. Among 16 cases described in detail keratoses are noted in 13 instances—81 per cent.

(6) The distribution of the lesions does not show marked predilection for the fingers, hands and feet as in the medicinal cases. This apparently is due to the fact that in the occupational cases the true Hutchinson type is rarer. In only three cases (2, 10 and 14) origin of the malignancy from a keratosis is stated. Most of the incidences belong to the multiple form frequently distributed all over the body. But only in two instances (cases 1 and 22) true multiple benign basal-celled growths seem to be present; in four cases (2, 5, 6 and 10) the biopsy showed squamous-cell cancers.

(7) So far as the predominance of the multiple type of epithelioma goes,

* To the 24 cases of Table VI may be added one case of Ayres and Anderson (1934); these authors mention one case of Bowen's disease and another case of epithelioma, but the latter may be identical with case 1 in Table VI. Besides, there are some doubtful cases reported, such as four from Henry (*cit.* Hueper, 1942) in workers exposed to an arsenic-containing powder. Hueper (1942) assumes a somewhat higher number of incidences (34), but it seems he has counted some cases twice over.

multiplicity seems more pronounced than in the therapeutic instances. But there are at least four cases with single growths (cases 1, 2, 5 and 6).

(8) Among the relatively small number of occupational arsenical epitheliomas four or five instances of pulmonary cancer are reported (cases 15, 16, 17, 18 and 20, the last perhaps identical with one of the former), whereas among the so numerous medicinal cases only two occurred. It is uncertain whether this is a mere coincidence, or if the irritation of the respiratory tract by arsenical dust is responsible. Unfortunately these cases are not reported fully; it is not mentioned whether melanosis and keratoses were present. A special committee was constituted to examine the problem (Merewether, 1943).*

The *treatment* of occupational arsenical cancer is the same as that in non-occupational cases. Of course the prophylaxis is here still more important. Therefore in many countries social-hygienic measures have been taken for the protection of the workers (Balthazard, 1930):

Prohibition of employment of women and young persons in factories and workshops in which arsenic is handled.

Notification of all cases of arsenical poisoning in factories and workshops in Great Britain by the Factories Act, 1937. Special notification of arsenical cancer seems not to be required (some other forms of cutaneous cancer, such as tar cancer, are notifiable—Statutory Rules and Orders, 1919, Vol. I, p. 710).

Preventive technical measures in the factories, such as avoidance of dust, use of dust-proof clothes and respirators, prohibition of meals taken in the work-rooms, provision of adequate facilities for washing, etc. (in Britain, Factory and Workshop Act, August 17, 1901, Part IV, 75; Factories Act, 1937, Part IV, 47 and 48).

Regular medical inspection of the factories and workshops. Workers with beginning arsenical keratosis or other signs of arsenicism are forbidden to continue their work, but have to receive compensation for loss of income.

The most simple and at the same time the most efficient way to deal with arsenical occupational cancer would be to abolish the general use of arsenic-containing material generally. That has been done partly by the prohibition of the use of arsenical colours in house painting, in wall papers, etc. As now nearly all occupational cases are caused by arsenical anti-parasitics and insecticides, the replacement of these by modern arsenic-free compounds such as DDT would settle the whole question.

### D. ARSENICAL CANCER IN ANIMALS (EXCEPT EXPERIMENTAL CANCER).

The first mention of the occurrence of chronic arsenicism in animals is again by A. Paris in 1820. He reports that in the neighbourhood of the copper-smelting works of Cornwall and Wales horses and cows commonly lose their hoofs, and not infrequently suffer from cancerous affection of their rumps. This assertion, quoted over and over again, occasionally even with unfounded additions, has been strongly criticized by Kennaway (1942, 1943; see also Hueper, 1942*b*, 1943). He objects that no attempt was made to show that arsenic was the active substance, and that the carcinomatous nature of the alleged tumours never has been established. Besides, the story of Paris has not been confirmed

* The members of the Arsenic Committee of the Medical Research Council are Professor M. J. Stewart (Chairman), Professor A. Bradford Hill, Dr. Donald Hunter, Dr. S. A. Henry, Sir Ernest Kennaway, Dr. A. N. Currie and Dr. Joan Faulkner (Secretary).

by later reports from that area. So Paris's report should never again be quoted as an example of arsenical cancer in animals.

In the district of Freiberg (Saxony) smelting works for many decades affections of horned cattle are known as "Huettenrauchkrankheit" (smelting fume disease). The clinical symptoms consist of chronic diarrhoea, cough, sclerodermia, chronic squamous eczema, paralytic weakness and emaciation. These symptoms led to the thought that they may be caused by chronic arsenic poisoning. This hypothesis was confirmed by the demonstration of arsenic in the organs of the diseased cattle (Haubner, 1878, *cit.* Prell 1936/37; Wobst, 1925; Hofmann, 1937, *cit.* Kennaway, 1942, 1943). But no case of cancer is mentioned in these observations.

Since 1930 extensive arsenical injuries have been observed among wild animals (red deer, roe deer, hares and foxes) in the Tharandt forest, also in the region of Freiberg. The disease, most marked in deer, is characterized by loss of hair, blackening of the underlying skin, thickening of the stratum corneum and extreme emaciation. In the preceding years many bee-swarms were lost, and the beekeepers attributed that to an intoxication by the smelting fumes of Halsbruecke and Muldenhuetten, 16 and 18 km. away. Prell (1936/37) was able to confirm this view by finding in the pollen up to 8·8 mg. per cent arsenic, in the dead bees up to 0·0011 mg. per bee (= 1·3–2·6 mg. per cent), the lethal dose for a bee being 0·0001–0·0002 mg. So he concluded that the beekeepers were right, and supposed that the disease of deer was due to the same cause. He emphasized the similarity of the symptoms with those of arsenical poisoning in man, drawing attention to the horny warts sometimes scattered over the skin. Besides, he often found a considerable amount of arsenic in the stomach (e.g. 47 mg. per cent in a hare), in the hair (up to 1·2 mg. per cent), less in the liver; sometimes no arsenic could be detected. Prell says nothing about occurrence of real epitheliomas. But Hofmann (1937, *cit.* Kennaway, 1942) discovered in the course of his investigations one case of cancer in a roe. Kennaway (1942), in his criticism of Hueper's view, pointed out that this single case among 143 deer and 27 hares exposed to arsenic gives, of course, no basis for the occurrence of arsenical cancer in animals.

A high mortality of bees and fish was also reported from Reichenstein (Kathe, 1937).

Nieberle (1939) described the occurrence of endemic carcinoma of the nasal mucosa in a highly inbred flock of Hampshire sheep in the Limbach estate in the remoter neighbourhood of the smelting works again of Freiberg in Saxony. Although in the tumours, in the brain and in the liver only extremely minute amounts of arsenic (0·01 mg. per cent) were found, Nieberle thinks that "a certain connection of this disease with the locality and with an injury by arsenic can scarcely be excluded." This cautious conclusion does not support Hueper's assertion that an environmental type of arsenical carcinoma has been observed in animals (Hueper, 1942; Kennaway, 1942).

### E. EXPERIMENTAL ARSENICAL CANCER.

It has been tried to prove the carcinogenic property of arsenic by experiments in animals—especially after the successful experiments with tar and its constituents. Different animals were used and different ways of application.

*Local Application on the Skin.*

Leitch and Kennaway (1922) painted a solution of potassium arsenite in alcohol (1·8 per cent, later 0·12 per cent) three times daily on the skin of 100 white mice through a long period. Most of the mice died within three months. In one of the survivers after 86 days a tiny wart appeared on the spot of application, after 98 days a second. The wart developed into a metastasizing squamous-cell epithelioma. In a second series (Leitch, 1923) not a single positive result was obtained. It is difficult to explain why only a single experiment was successful. Spontaneous epitheliomas of the skin are very rare in mice, and the localization on the very spot of application seems to prove that the cancer in this case was caused by the administration of arsenic. The fact that no more positive results were obtained may partly be explained by the difficulty in keeping the animals alive for a sufficient time. Probably there must be—besides the administration of arsenic—some other unknown local or general factors influencing the success of such experiments. Lipschuetz (1924), repeating these experiments of painting with an alcoholic solution of arsenious trioxide, recorded only hyperkeratoses and pigmentations but no malignancy. Raposo (1928) painted the inner surface of the ears of rabbits with a mixture of 10 per cent arsenic trioxide and vaseline. Besides hyperkeratoses and papillomas in several cases he obtained a cancroid in one case. It is not reported whether controls with vaseline alone were performed.

*Oral Application of Arsenic.*

Leitch and Kennaway (1922) obtained negative results in rats and mice, Maisin (1934) in mice. Cholewa (1934), feeding 10 mice with arsenic for one year (0·05–0·1 mg. daily), observed in two of the animals blastomas of the lungs ; in one rabbit a sarcoma of the ear after 6 months. In the experiments of Finner and Calvery (1939) with dogs and rats fed with arsenious trioxide and with arsenates, pathological changes developed but no growth is reported. Hueper (1942*a*) experimented with congenitally hairless rats ; in a series of 10 animals he obtained in one rat surviving for 21 months a cancer of tricho-epitheliomatous structure. Nothing is said about the frequence of spontaneous cancers in these animals ; it is only stated that they develop normally papillary warts in their hyperkeratotic skin (that was the reason why Hueper used them for his experiments).

*Parenteral Application of Arsenicals.*

Bierich (1922), as well as Moeller (1923) and Lipschuetz (1924), injecting white mice hypodermically with Fowler's solution, had negative results. Askanazy and Girod (1926) injected rats with arsenious acid ; in one of these animals after some months an osteoid sarcoma developed from the follicle of a cysticercus. White (1927) reported five negative experiments in chickens. Schinz (1942) made deposits of (? metallic) arsenic by injecting it in the thighs of rabbits. In one animal after six years a pulmonary giant-cell sarcoma developed. Spontaneous malignancies are said to be quite rare in rabbits. Ebert (1929) observed, after intradermal application of a small dose of arsphenamine given to a man of 63, microscopical changes which he describes as " precancerous," similar to Bowen's disease.

As the experiments with arsenic alone did not give definite results, other ways were tried.

Puccinelli (1930*a*) found very little influence of arsenic on the appearance of spontaneous primary lung tumours in mice.

Many authors studied the effects of arsenicals on pre-existent tumours in animals. The results were widely divergent. Some investigators, as Sticker (1911), Funk (1915), Bierich (1922), Moeller (1923), Rosen (*cit.* Schiller, 1926), and Schiller (1926) found no effect. Montemartini (1928) reported an inhibitory effect of intravenous injections on transplanted cancers in mice.

Others observed a stimulating effect—Antonoff (1928) on Flexner-Jobling rat carcinoma, Califano (1931) on Teutschlaenders strain in chickens ; Hueper and Itami (1933) think that intravenous injections of neoarsphenamine were leading toward increased malignancy of spontaneous tumours in mice. Dustin and Gregoire (1933) experimented with mice bearing sarcomas type Crocker ; as soon as 9 hours after injection of sodium cacodylate they observed a " veritable explosion caryocinétique " in the transplanted tumour (see p. 231).

Other investigators studied the influence of arsenic on the development of experimental tar cancer. Schiller (1926) reported that arsenic prevented the precancerous changes in the skin of tarred mice from developing in carcinoma ; but it did not influence a fully established tar cancer. Puccinelli (1930*a*) found a slight delaying action of arsenic trioxide on the appearance and growth of tar cancer in white mice. On the other hand, Ciechanowski, Morozowa and Wilhelmi (1925) observed a distinctly accelerating effect of oral ingestion of potassium arsenite on the development of tar carcinoma in rabbits.

Following the theory that arsenic may be favourable to the development of growth from embryonic cells, other investigators combined the ingestion of arsenicals with inoculation or implantation of embryonic cell material.

Carrel (1925) started from the observation that the filtrable agent of the Rous sarcoma is able to transform *in vitro* macrophages into sarcoma cells. Supposing that this filtrable agent is produced by the cells through the influence of a non-specific chemical substance, he injected 16 chickens with a mixture of embryonic chicken pulp and arsenious acid. In four of these fowls after a few days fatal fusicellular sarcomas developed. The tumours contained a filtrable agent resembling a virus. Carrel suggested that the arsenious acid had caused the formation by the tissues of this agent.

These sensational experiments were repeated by many investigators ; but only some of them had similar results.

Fischer (1926) reported such a similar result in a chicken which was inoculated with embryonic spleen tissue cultivated in a medium containing a very small quantity of arsenic trioxide. White (1927) confirmed Carrel's results ; the sarcomas arose from the embryonic tissue, not from the tissues of the host. Haagen (1928) also repeated Carrel's experiments, with a positive result ; in addition he injected normal monocytes from chickens together with very dilute solutions of arsenious acid in 24 fowls ; after three weeks rapidly growing metastasizing and transplantable sarcomas of the Rous type developed in 16 of the animals. Petroff and Krotkina (1928) injected 97 rats with embryonic pulp together with a suspension of arsenious acid ; in three of the rats six months later sarcomas appeared. It seemed improbable that these were spontaneous tumours as they arose on the spots of injection. On the other hand the authors reported the development of sarcomas after injection of embryonic pulp alone ; so the aetiological role of arsenic in these experiments is not clear (spontaneous tumours were very rare—only one in 300 rats). Krotkina (1934) observed metastasizing transplantable growths after injection of embryonic pulp in a rat previously treated with arsenic.

But all the other authors repeating Carrel's experiments were not able to confirm his results :

Pentimilli (1926), Deelmann (1928), Maisin and Dupuis (1929), Kauffmann (1929), Felloni (1930), Laclau and Pillado-Matheu (1930) and Lapidaria. Only small embryomas (teratomata) occurred sometimes but no cancers.

Negative results in rats and mice were obtained by Collier and Hartnack (1929), Puccinelli (1930*a*, *b*), McJunkin and Cirkit (1929). McJunkin and Cirkit succeeded only in one out of 50 rats in getting a cystadenoma suggesting malignancy.

Some authors, such as Deelman (1928), Maisin and Dupuis (1929) and Begg and Cramer (1929), suggest that Carrel's results were possibly due to minute contamination with traces of Rous' sarcoma virus, not to the presence of arsenic.

Askanazy (1926) examined the influence of arsenic on the growth of embryonic tissues (stomach, intestine) transplanted into the stomach of rats receiving a small quantity of Fowler's solution with the drinking water. After 11 months a fusicellular metastasizing sarcoma of the stomach developed in one out of nine rats ; in two others an adenocystoma appeared after 13 and 14 months respectively, also on the spot of implantation ; whereas in 18 controls without arsenic the transplants disappeared except in one case in which a small sarcoma of the liver arose. Askanazy pointed out that for the genesis of blastomas the presence of several factors may be necessary : besides a general constitutional predisposition (" allgemeine Koerperbereitschaft ") and a local predisposition (represented in these experiments by the embryonic tissue), a third factor, an irritant of oligo-dynamic efficiency, e.g. arsenic.

Fischer-Wasels (1928, 1929) and his co-worker Buengeler (1930) combined two supposed carcinogenic factors in a different way. They produced chronic arsenicism by feeding or injecting rabbits or mice with potassium arsenite for many months ; then they injected scarlet red oil into the skin or (in female mice) into the breast, or they made local burns to induce local regenerative processes. Among 18 rabbits, in one a cancer of the skin developed ; and in 5 mice (= 25 per cent) adenocarcinomas of the breast, all controls remaining negative. Spontaneous cancers of the breast were rare in these mice (only 4 cases among 2000 animals). The authors' interpretation was that chronic arsenic poisoning had produced a general pre-disposition for cancer after a sensitizing period of some months, the local procedure a local anlage. Further they found out that the general disposition for growth in these experiments with arsenic was characterized by certain changes of the metabolism of the tissues, proved by Warburg's methods : decrease of oxygen consumption (checking of the respiration), increase of anaerobic formation of lactic acid, and by aerobic glucolysis.

Summarizing the experiments on animals it can be stated that arsenic alone has given only doubtful results. Difficulties were caused by the occurrence of spontaneous tumours in the animals, by the high toxicity of arsenic leading to an early death of the animals, and by the fact—well known from clinical experience—that arsenic has a very long latent period for the production of malignancy, usually surpassing that of the average life of the animals. The combined experiments—arsenic plus embryonic cells or arsenic plus local influences on the tissues—have proved more successful. No critical objections to these experiments seem to have been put forward so far.

### F. PATHOGENESIS OF ARSENICAL CANCER.

Even now a really satisfactory theory of the pathogenesis of arsenical cancer does not exist, although many clinical observations and much experimental work are available.

It is a fact that arsenical malignancy is very often preceded by other manifestations of arsenical poisoning, especially keratoses and pigmentation. Therefore, arsenical cancer might be conceived as a " second disease " in the sense Roessle used this term.

Stelwagon, Gilchrist (*cit.* Hueper, 1942*b*), Dubreuilh (1910) and de Silva (1918) held the opinion that arsenic is not directly responsible for the cancer, but only for the development of the keratoses. " C'est l'arsenic qui fait la kératose, mais la kératose évolue ensuite spontanément vers le cancer " (Dubreuilh). This view was supported by the fact that keratoses of different origin sometimes undergo malignant transformation as well, e.g. keratoses due to X-rays, radium, irradiation, senile keratoses and leukoplakia oris.

This view was opposed by Schamberg (1907*a*) : " The mere fact that there is an intervening hyperkeratosis does not relieve arsenic from the responsibility of the production of the malign disease." He stressed the fact that the vast majority of keratoses (congenital keratoses, tylosis, clavi, etc.) are not in the least inclined to become malignant. Besides, this keratosis hypothesis is insufficient, because it is now well established that many arsenical growths arise in previously normal skin.

Stewart (1934, *cit.* Hueper, 1942*b*) suggests that the pigmentary disturbances set up by arsenic may be related to the development of malignancy. It has been referred to that melanin contains cyclic hydrocarbons which might be carcinogenic. But in the majority of cases of arsenical cancer abnormal pigmentation is missing.

An explanation of the carcinogenic property of arsenic has to start from the pharmacological qualities of that poison ; unfortunately they are still far from being clear.

It is known that arsenic has local as well as—after its resorption—general effects.

*Local effects of arsenic.*

Arsenic working as a *local irritant* in high concentrations causes necrosis. That local irritation can play a role in the causation of malignancy is generally accepted. Further, it is certain that in some cases of chronic arsenicism, especially in occupational cases, signs of external irritation occur : eczema of hands, conjunctivitis, perforation of the nasal septum, etc. But the usual arsenical cancer of the skin cannot be caused by such a direct irritation ; for it does not appear at the sites of application. If that be the case, the cancer—

at least in the medicinal cases—would be expected in the mouth, oesophagus, stomach and in the intestines ; frequent cancer of the jaws would be expected after the common use of arsenic by dentists, but not localization on the skin. Besides, the nearly always negative result of external experimental application of arsenic speaks also against local irritation as the main etiological factor.

So arsenical cancer differs essentially from other kinds of malignancy, such as tar cancer, X-ray cancer, radium cancer, in which the main effect is seen at the site of application (Semon, 1922 ; Guggenheim, 1933,). But it must be remembered that Murphy and Sturm, in mice subjected to painting with tar, observed an increased incidence of pulmonary cancer, and that similar results were reported by Schabad (1935) and Cook, Haslewood, Hewett, Hieger, Kennaway and Mayneord (1937). That seems to show that tar, besides its well-known local action, has also a carcinogenic property after its resorption.

Only a few observations were advanced by some authors as an indication that local external irritation may occasionally play a part in arsenical malignancy. It was suggested that in some occupational incidences malignancy arose on the place of application (Ullmann, 1930 ; Fassrainer, 1936 ; Aitken, 1944). Eggers (1932) quotes especially the cases of sheep-dip cancer as caused by constant irritation of the skin by arsenical dust ; but the distribution of the lesions in these cases was mainly the same as in medicinal multiple superficial epitheliomas (see p. 223). The occurrence of pulmonary cancer in workers exposed to inhalation of arsenical dust was also attributed to local irritation, but without real proof. Besides, the occasional occurrence of local "precancerous" changes after injection of arsphenamine (Ebert, 1929) may be mentioned, and the very rare positive experiments with external application in animals (see p. 226).

*General Pharmacological Effects of Arsenic.*

Hermann (1879), Schmiedeberg (1906) and Heubner (1907) suggested a paralysis of the capillaries as the fundamental general pharmacological effect of arsenic after its resorption. They attributed to it the frequent inflammations of the mucous membranes and of the skin. But it is not clear in which way such poisoning of the capillaries could lead to malignancy. Besides, the view of these authors was opposed by others (Heffter and Keeser, 1927).

Ringer and Murrel (1878/79) declared arsenic to be a poison to all nitrogenous tissues. And since then many investigators held the view that the basic effect of arsenic is its *action on the metabolism* of the cells.

Binz and Schulz (1879) suggested that the action of arsenic is connected with the mutual transformation which arsenious acid ($As_2O_3$) and arsenic acid ($As_2O_5$) undergo in the presence of living protoplasm ; they pointed out that the effects of arsenic result from the development of active oxygen originating in these processes, stimulating the cells eventually to exhaustion and death. Brooke and Roberts (1901) supported this *oxidation hypothesis* as the best explanation of the facts. But it seems that the supposed increase of cellular oxidative processes has not sufficient experimental basis. Even the famous basal experiments of Binz and Schulz have been criticized ; the reduction of $As_2O_5$ has been proved, but not the oxidation of $As_2O_3$ (see Heffter and Keeser, 1927).

In contrast to the oxidation theory, it is known for a long time that arsenic in very small quantities checks the processes of oxidation and reduction in the body (Onaka, 1911). That may result in an increase of body weight and body fat, often aspired to and achieved in the therapeutic use of the drug ; increase of body protein and bone tissue was observed too (Weiske, 1875).

Barry, Bunbury and Kennaway (1928) demonstrated *in vitro* such a retarding action of arsenic upon three different oxidation-reduction processes, namely : hypoxanthine-xanthine oxidase of rat or mouse-skin, oxidation of acetaldehyde with colloidal platinum, and the system propylaldehyde (or acetaldehyde), glycerine and phosphate. The authors themselves emphasize that one cannot with any certainty compare reactions in the test-tube with changes in the human skin, which require months and years. But they are in accord not only with the just-mentioned results of Onaka and similar results of Dresel (1926 and 1928), Banga, Schneider and Szent Gyeorgyi (1931), and of Victorisz (1931) and others, but also with the above-mentioned work of Fischer-Wasels (1928 and 1929) and Buengeler (1930), who found in animals predisposed to cancer by ingestion of arsenic a decrease of oxygen consumption and increase of anaerobic formation of lactic acid.

Barry, Bunbury and Kennaway express the idea that arsenic may induce cancer not by any direct action, but by causing accumulation in the tissues of some (carcinogenic) organic compounds which would otherwise be oxidized or reduced to other forms.

Bunsen had qualified arsenic as a " protoplasmic irritant." Many authors stick to the view that it acts as an " auxetic," by *direct chemical stimulation* of the epidermal cells leading to their proliferation and " cancerization " (Bland-Sutton, 1916 ; Osborne, 1925 ; Ullmann, 1930). Dustin and Piton (1925, 1929) and Piton (1929) observed after injection of arsenicals (especially cacodylate of soda) in mice numerous mitoses in the thymus, in the lymph-nodes and in the Lieberkuehn glands—" action caryocinétique." Rocmans (1930) attributed this effect to an alkalosis, but this view was criticized by Dustin (1930). On the other hand Throne and Myers (1935) have found in some patients with arsenic retention and (non-malignant) lesions of the skin a rise in the $CO_2$ content of the blood suggesting an alkalosis.

Ullmann (*cit.* Mayer, 1933, and Throne and Myers, 1935) reported at the International Congress of Dermatology, 1930, that a solution of one part of arsenic in 40 millions of water, i.e. 0·0025 mg. per cent, added to embryonic cells acts on them in a tumour-forming manner ; he asserts that this action on the cells of the hair sheaths and walls of sweat glands explains the development of arsenical cancer.

To explain the so well marked *predilection* of arsenical cancer *for the skin* one has to remember that arsenic has a special affinity for structures of ectodermal origin. After ingestion it accumulates in the epidermis, sweat and sebaceous glands and their ducts, hair follicles and hairs. Both Bruenauer (1921) and Osborne (1925) were able to identify it there with the histochemical method. Some authors ascribe this affinity to a special relation of arsenic to the keratin containing large amounts of sulphur (cysteine, etc.).* So it may be understood

* See Labes, 1929, and Rosenthal and Voegtlin, 1930.

that arsenic acts in these places as an irritant, causing keratinization and possibly malignancy.

Certain authors asserted that the keratoses originate from the orifices of the sweat glands (Ullman, 1930; Bruenauer, 1921), and suggest that this fact is connected with the excretion of arsenic in the perspiration. But this view is not generally confirmed (Brooke and Roberts, 1901).

One of the difficulties in understanding the pathogenesis is the often very *prolonged interval* between cessation of the poison and starting of the malignancy (see p. 210). Some authors doubt whether after such a long time arsenic can still be present and act in the skin. Ullmann (*cit.* Hueper, 1942*b*, p. 54) suggests that the cells are " cancerized " during the exposure to arsenic, but remain quiescent for a long time. But the example of argyrosis shows, as Franseen and Taylor (1934) remark, that a drug can remain in the skin for a lifetime; and the presence of arsenic in the skin after a very long time was demonstrated by chemical and histochemical methods (Montgomery, 1935).

Another fact to be explained by the theory is that arsenic produces epitheliomas *only in very few* of the many persons exposed to that poison. Many thousands of patients are receiving this drug, but in comparatively few arsenical keratoses develop, and in still fewer epitheliomas (Wile, 1912; Sulzberger, discussion to Bloom, 1936; Satenstein, *ibid.*). And in the positive cases the dose of arsenic as well as the duration of the latent period and the type of epithelioma show very great differences. Ebert (1929) observed precancerous changes after injection of arsphenamine only in certain persons. And in analogy the attempts to produce experimental cancer with arsenic in animals were successful only exceptionally.

All these facts suggest that in " arsenical cancer " *arsenic is not the only aetiological factor*; others must be present too. On this point all authors seem to agree (Schamberg, 1907; Wile, 1912; de Silva, 1918; Askanazy, 1926; Buengeler, 1930; Milch, 1932; Frost, discussion to Ayres, 1934).

Some observations suggest that the *individual reactivity* to arsenic shows a marked variability (Schondorf, *cit.* Hueper, 1942, Throne and Myers, 1935). Throne and Myers have suggested that these differences may be related to variations in the excretion of the poison. Cornbleet (discussion to Montgomery, 1941) found that patients showing arsenical keratoses take a longer time to excrete a given amount of arsenic than control patients; he concluded that in such patients the drug is retained in the epidermis for protracted periods so as to irritate and to produce keratoses and cancer.

Other authors suggest a predisposing local abnormality of the skin, perhaps a *local sensitivity* (idiosyncrasy) to arsenic analogous to contact dermatitis. Investigations with patch tests (Sulzberger, discussion to Bloom, 1936) had no conclusive results.

Another fact suggesting a special sensitivity of certain individuals is the high frequency of psoriatics among the patients with medicinal arsenical cancer (see p. 194).

The role of *heredity* as a predisposing factor has been mentioned by several authors. It is well known that hereditary predisposition is very important in certain kinds of clinical as well as experimental malignancy. It is noteworthy that in psoriasis too a hereditary factor is well established (Case 130 in Table I belongs to a psoriatic family). It is true that in only a few cases of arsenical

### Table I.

| No. | Author. | Sex. Age. | Drug. Disease. | Keratoses. Pigmentation. | Localization of epitheliomas. | Biopsy. Metastases. Death. |
|---|---|---|---|---|---|---|
| 1 | White (1885), "case 1"; Hutchinson (1887, 1888) case Dr. W. (1895, 1898, 1903) (picture) | M. 44 | S. Fow., S. Don.; large quant. beg. 20 y. ago f. 6 y. | Ker. p. and fingers (corns) beg. 10 y. ago f. 6 y. | One: r. p. on ker. beg. 1–2 y. ago.<br>One: betw. l. fore- and middle finger on ker. | Metas. Death |
| 2 | White (1885), "case 2" | M. 52 | As. apparently many y. | Ker. p. | One: p. suff. r. wrist on ker. 10 y.<br>One: betw. fingers on ker.<br>Three: nr. anus, upon penis, groin | Carc. Death. |
| 3 | v. Hebra (1887), *cit.* Pye-Smith (1913) | M. ? 50 | As. Psor. (probably) | Over 100 "warts" on the body | One: r. arm.<br>One: r. thigh, and other parts on warts | Carc. Death. |
| 4 | Hutchinson (and Tay) (1887, 1888) | M. 34 | As. long time. Psor. | Ker. p. and s, feet s. 1½ y. | One: scrot., some months preceded by a wart | . . |
| 5 | Hutchinson (and Chiene) (1887, 1888) | M. 55 | As. many y. Psor. | Ker. p. and s. (corns) | One: l. s.<br>One: toe on ker.<br>Later: carc. of stomach | Death. |
| 6 | Hutchinson (and Allbutt) (1887, 1888) | F. 25 | As. repeatedly. Pemphigus | Roughness of p. | One: nr. crista ilei on spot of rough skin | Metas. |
| 7 | Lane, W. A. (1894); see also *A. D. & S., Chic.*, **131**, 46 | M. 60 | S. Fow. f. 30 y. Psor. | Not stated | 11 growths:<br>4 ulc. l. forearm in healthy skin<br>7 ulc. scrotum and perineum | Ep. |
| 8 | Hutchinson, J. (1894) | M. 35 | As. 20 bottles, 10 y. prev., f. some months. Acne | Ker. p. | One: hand, on ker., 10 y. after stopping As.; 6 y. later two scrotum | Carc. |
| 9 | Hutchinson, J. (and Bullock) (1898, 1903) (picture) | M. 46 | As. liberally f. many y. Psor. | Ker. s., legs and elsewhere | One: above pubes.<br>One: back (s. 3 y.) | Metas. Death. |
| 10 | Hutchinson, J. (1898) | F. 45 | As. f. 20 months. Epilepsy | Ker. p. and s. some y. after taking as. | One: r. hand on ker.<br>One: below l. breast | Metas. Death. |
| 11 | Hartzell and Stellwagon (1898, 1899) (picture); Stellwagon (1910 ?) | F. 35 | As. f. long period. Psor. | Ker. p. and s. 9 y. aft. beg. of psor. | Num. small superf. ulc. on ker. of hands and fingers, consid. time after beg. of ker.<br>Two: l. heel<br>One: over l. breast<br>One: l. groin | Carc. |
| 12 | White, J. C. (1899) | M. | As. Psor. | Ker. p. and s. | One: gr. toe, on ker. | . . |
| 13 | Hyde (1899) | M. | As. Psor. | Ker. p. and s. | One: middle finger, app. on ker. | Metas. |
| 14 | Ullmann (1898, 1900) (picture) (1917) | F. 37 | S. Fow. f. 6–7 y. Acne | Ker. p. and s. Pig. | One: forehead, in ker.<br>One: l. heel<br>One: r. ankle | Bas. c. ep.<br>Sq. c. ep. |
| 15 | Crocker and Pernet (1901) | M. 60 | As. 40 y. ago, f. 2 y. Psor. | Warty specks, hands | One: r. hand, uln. border on a speck, 2 y. ago<br>Two others in neighbourhood | Sq. c. ep. Excision. Rec. |
| 16 | Hutchinson, J. (1902, 1903) (plate) | M. 70 | As. f. long periods. Psor. (?) | Ker. p. and s. | Soon after ker.<br>One: r. forefinger on ker.<br>One: back of r. shoulder | Carc. |
| 17 | Darier (1902), *cit.* Pye-Smith (1913*b*) "case 22" | M. 47 | As. large quant. Chr. bronchit. | Ker. p. and s. (corns). Pig. | Three: hands on ker.<br>One: neck<br>One: r. upp. eyelid | . . |

TABLE I (*continued*).

| No. | Author. | Sex. Age. | Drug. Disease. | Keratoses. Pigmentation. | Localization of epitheliomas. | Biopsy. Metastases. Death. |
|---|---|---|---|---|---|---|
| 18 | Brocq (1902), *cit.* Schamberg (1907) | M. 35 | As. large quant. 15 y. (ab. 10 g. $As_2O_3$. Chr. bronchit. | Ker. p. and s., warty pig. | Two: r. p. on ker. Two: l. p. One: neck | Carc. |
| 19 | Hutchinson, Browne and Blackstone (1903) "case 4" | M. 62 | As. f. many y. | Ker. p. and s. | One: buttock One: face | Carc. |
| 20 | Schamberg (1907), "case 1" | M. 62 | S. Fow. *ca.* 30 drops daily f. 3 months 25 y. Psor. | Ker. p. and s., warty pig. | One: dors. r. thumb on ker. | Carc. |
| 21 | Hartzell* | M. 55 | As. Psor. | Ker. p. and s. | One: r. p. on ker. | Amputat. Rec. Death. |
| 22 | Dubreuilh (1910), "case 3" | F. old | As. large dos. Pemphigus | All signs of chron. arsenicism | Carc. mammae | .. |
| 23 | Dubreuilh (1910), "case 4" | M. 74 | S. Fow. large quant. 15–20 drops 20 y. Psor. | Ker. p. and s. s. ab. 15 y. | One: l. thumb on ker. One: r. middle finger 2 y. Sev. les. trunk | Sq. c. ep. |
| 24 | Gray (1912) | F. 56 | As. 32 y. | No ker. | One: nr. glut. cleft, continuous with a patch of psoriasis 10 y. | Bas. c. ep. |
| 25 | Loewenberg (1912) "case 1," see also disc. to Ullmann (1922) | M. 50 | As. Psor. | Ker. p. and s., backs of hands and feet, warty | One: back l. hand on a ker. papillomatous, a. others on. One: flex-side of forearm, in normal skin | Sq. c. carc. Amputat. Rec. Sq. c. ep. |
| 26 | Loewenberg (1912) "case 2" | M. 50 | As. Psor. | Ker. p., warty | One: r. p. 1½ y. from a ker. | Death on interc. dis. |
| 27 | Wile (1912) (plate) | M. 29 | As. large quant. 10 y. ago f. 2 y. f. complexion | Ker. hands and feet diffuse and punct. 4 y. after starting As. No pig. | One: l. ring-finger on ker. | Carc. Metas. |
| 28 | Schamberg, J. F. (Philad. Derm. Soc., *cit.* Pye-Smith, 1913*b*), "case 28" | M. 43 | As. large quant. | Ker. trunk. Telangiect. | One: r. nipple. Num. epith. all over the trunk | .. |
| 29 | Nutt, Beattie, and Pye-Smith (1913 *a* and *b*) | F. 29 | As. f. 7 y., begin. 22 y. ago. Psor. | Ker. p. and s. (corns) 1½ y. under wedding ring Ker. on the whole body | One: l. ring finger on ker. 10 months. One: l. middle finger One: pubes on ker. Three: vulva, strawberry-like, s. 1½ y. | Sq. c. carc. Amputat. Rec. Death. |
| 30 | Fordyce-McKee (1914) | M. 37 | S. Fow. f. long per. Psor. | Ker. p. Pig. | 14 les. back, arms, leg | Bas. c. carc. |
| 31 | Trimble (disc. to Fordyce-McKee, 1914) | M. 65 | As. f. long per. Psor. | Ker. | One carc. | .. |
| 32 | Bland-Sutton (1916) | F. 60 | S. Fow. more or less contin. f. 30 y. Psor. | Nothing noted | One: below l. knee in patch of Psor. 3 y. | .. |
| 33 | Ullmann, K. (1917, 1932), "case 2" | F. 33 | S. Fow. 15–17 y. ago f. 1–2 y. Chlorosis and complexion | Ker. p. and s., warty, mult. ker. Leukoplakia oris. No pig. | One: heel, 1 y. ago One: index, ½ y. ago Later: one tongue Carc. of mucosa oris on leukoplakia | Sq. c. carc., pagetoid. Death. |

* Discussion to Schamberg (1907). See also Pye-Smith (1913*b*), "case 25."

TABLE I (*continued*).

| No. | Author. | Sex. Age. | Drug. Disease. | Keratoses. Pigmentation. | Localization of epitheliomas. | Biopsy. Metastases. Death. |
|---|---|---|---|---|---|---|
| 34 | Ullmann, K. (1917), " case 3 " | M. | S. Fow. f. 2 y. M. mac. Werlhof | Acute ars. pois. 1 y. later; ker. p. General melan. | One: heel, in ker. | Death (tub.) |
| 35 | Stillians, A. W. (1919) | M. 57 | As., large dos. f. 1 y., 30 y. ago, f. prevent. malaria | Ker. s. 25 y. | One: l. p. 20 months | .. |
| 36 | Pusey (disc. to Stillians, 1919) | .. | As. Psor. | Ker. hands | One: hand, in ker. | .. |
| 37 | Wise, F. (1920) | M. 42 | S. Fow. 20 y. ago f. 6 weeks. Pemphigus | Ker. p., sacr. and lumb. reg. | One: sacr. reg. in ker. | Sq. c. carc. |
| 38 | Hamilton, G. R. (1921) | F. 46 | S. Fow. s. 36 y. 9 months in a year. Psor. | Ker. p. and s., back of hds. (corns) s. 20 y. | One: r. arm not in a patch of psor. | Sq. c. carc. (mal. papillom.) |
| 39 | Alexander, A. (1921) | M. 35 | S. Fow. 15 y. w. interrupt. Psor. | Ker. p. (not s.) s. 137. Pig. s. 13 y. | One: r. thigh on a patch of psor. | Bas. c. carc. |
| 40 | Ullmann, K. (1922) | .. | S. Fow. sev. y. | Ker. (p. and s. ?) | One: toe<br>One: leg | Death. |
| 41 | Ullmann, K. (1922) | .. | S. Fow. sev. y. | Ker. (p. and s. ?) | One: toe<br>One: finger | Death. |
| 42 | Semon, H. C. (1922) (picture) | M. 40 | As. f. 7 y. Psor. | Ker. p. and s. (warty) after 7 y. Pig. | One: r. sole in ker. 14 y. after stopping As. | Sq. c. carc. Amputat. |
| 43 | Nander (1923) | M. 65 | Pil. asiat. 2 g. in 1 y. Psor. | Ker. hands and feet. Pig. | Mult. ulc. both shins in psor. spots<br>Forehead: non - malig. adenomata | Sq. c. carc. |
| 44 | Oliver (1923) | M. 42 | As. over a long period. Acne | Ker. p. and backs of the hands | 4 or 5 ep. on the body | .. |
| 45 | Haxthausen, H. (1923) | M. 65 | As. pills (prob.) 3 *t.d.* during 9 y. beg. ab. 22 y. ago " for the nerves " | Num. ker. trunk and extr.; no ker. p. and s.; begin. 4–6 y. after stopp. the pills | Mult. (20) carc. spread over the trunk, one in l. ax. ulc. | .. |
| 46 | Aliferis (1924, " case 1 " | M. 47 | S. Fow. and As. pill. Psor. | Ker. | One: r. knee<br>One: nr. crena ani (not certain whether on patch of psor. or on ker.) | Sq. c. carc. |
| 47 | Aliferis (1924), " case 2 " | M. 67 | S. Fow. and As. pills sometimes in 33 y. Lichen rub. planus | Ker. Hyperker. sub ungualis. Erythrodermia | Five, arising from ker., breast, back | Carc. death. |
| 48 | Barber, H. W. (1925) | M. 70 | S. Fow. 6 drops 3 *t.d.* Psor. | Ker. p. and s. Pig. Chr. arsenical dermatitis | Three (?) fungating tumours: scalp, face, ears<br>Two flat growths upper arms | .. |
| 49 | Schwartz (1926) | M. 35 | Medicine cont. As. s. 8 y. (?). Dermatitis herpetif. | Ker. p. and s., fingers. Hyperhidros. General keratos. Pig. | One: r. thumb, palmar surf. der. from ker. les. besides groups of papules and vesicles on trunk; some resemble Bowen d. | Sq. c. carc |
| 50 | Levin, O. L. (1926), " case 1 " (see also Anderson, 1932) | M. 45 | Arsphenamine As. p. os and inject. sev. y. Syphilis. Psor. | Ker. p. and s., trunk | One: l. chest<br>One: l. arm<br>One: neck | Bas. c. carc.<br><br>Bas. c. carc. |
| 51 | Levin, O. L. (1926), " case 2 " (see also Fraser, 1929) | M. 35 | As. inject. and asiatic pills. Dermatitis herpetif. ? | Ker. p. and s., buccal muc. | One: l. thumb | Sq. c. carc. Metas. |
| 52 | Herxheimer (1939) | M. | As. f. 10 y. Psor. | Nothing noted | Sev., trunk | .. |

TABLE I (*continued*).

| No. | Author. | Sex. Age. | Drug. Disease. | Keratoses. Pigmentation. | Localization of epitheliomas. | Biopsy. Metastases. Death. |
|---|---|---|---|---|---|---|
| 53 | Harbitz, H. F. (1927) | F. 46 | Arsphenamine inj. 15 y. ago. Syphilis | . . | Fibrosarcoma on spot of injection (left intersc. reg.) 12 y. after inj. | . . |
| 54 | Oliver and Finnerud (1928) | . . | S. Fow. beg. 22 y. ago f. 11 y. Acne | Ker. p. (warts) 5 y. after starting As. | One : lumbo-sacr. reg. One : l. thigh | . . |
| 55 | Fraser (1928), "case 1" | M. 59 | As. beg. 52 y. ago f. 12 y. Chorea | . . | Ab. 200 lesions of Bowen's d. beg. 8 y. after start. As. 8 y.; later more les. trunk and limbs | . . |
| 56 | Hofmann (1928) | M. | As. f. a long per. Psor. | Ker. extremit. esp. feet, back | Several feet, back, thigh | . . |
| 57 | Ramel (1929) | M. 68 | As. pills more than 30 y. ago | Ker. hands and feet, warty | One : hand, on ker. (after contusion) | . . |
| 58 | Cole and Driver (1929) | M. | As. (probably) 20 y. f. an eruption | Ker. hands and finger | One : l. p. 20 months | . . |
| 59 | Konrad (1929) | M. 36 | As.-containing mixt. s. 20 y. | Ker. p. and s. s. 10 y. | One : r. s. | Sq. c. carc. |
| 60 | Fuhs (1930) | F. 65 | S. Fow. f. more than 20 y. Psor. | Ker. hands and feet s. 18 y. | One : r. thenar Sev. ulc. hands and feet on ker. | Sq. c. carc. Metas. |
| 61 | Rasch (1930) | . . | As. 30 y. Psor. | Ker. soles. Pig. | Two : l. calf | Sq. c. carc. |
| 62 | Haagensen (1931) ("case 1") (photo) | M. 55 | S. Fow. f. the last 15 y. w. interv. 4–10 dr. *t.i.d.* Psor. | Ker. p. and s. 4 y. after starting As. | Six : fingers of both hands | Sq. c. carc. |
| 63 | Haagensen (1931), "case 2" | M. 35 | S. Fow. 8 y. Pruritus | Ker. and fiss. p. and s. | One : l. thumb | Sq. c. carc. Metas. |
| 64 | Haagensen (1931), "case 3" | M. 46 | S. Fow. beg. 16 y. ago periodically. Psor. | Nothing noted | One : l. upp. arm One : l. neck, both s. 4 y. | Bas. c. carc. |
| 65 | Haagensen (1931), "case 4" | M. 31 | S. Fow. 14 y. ago, 30 dr. day f. 6 months. Nervousness | Ker. p. and fingers warty, s. 14 y. | One : nr. anus, s. ab. 2 y. Numerous superf. les. trunk, legs, 12 y. aft.; s. ab. 2 y. | Sq. c. carc. and dermatitis. |
| 66 | Stillians (1931) | M. 58 | S. Fow. 30 y. prev. f. 2 y. Psor. | *Nil* noted | Sev. : trunk One : forehead One : r. arm.; s. many y. | . . |
| 67 | Doty, C. A. (1931) | M. 69 | S. Fow. large doses 40 y. ago, and 30 pints "psoriasis-medic." | No ker. General pig. | A number of small les. trunk, some ulcerated (prob. superf. ep., see Anderson, 1932) (Bowen's dis. ?) | . . |
| 68 | Gross, P. (1931) | F. 48 | 3 y. psor. S. Fow. f. a long time. Skin dis. in childhood | No ker. No pig. | Mult. superf. epith. s. 20–35 (?) y., 3 in face, mult. on trunk | Bas. c. carc. |
| 69 | Milian, G. (1931), "case 1" (see also Milian, 1929) | F. 47 | As. | Ker. Pigment. Erythrodermia exfoliativa | Proliferat. of a naevus (melanot. sarcoma ?) 7 y. later : epithelioma perlé (cheek) | Sq. c. carc. |
| 70 | Milian, G. (1931), "case 2" | F. | As. | Ker. not noted. Erythrodermia exfoliativa | Carc. of r. breast | . . |
| 71 | Fischer, W. (1932) | M. 50 | As. long time. Psor. | Ker. p. and s. | Several : r. p. and l. calf | Sq. c. carc. |
| 72 | Andrews, N. P. (1932) | F. 75 | S. Fow. 60 y. over unknown per. | Nothing noted | Mult. superf. ep. back, chest, thighs, 40 y. | Bas. c. carc. |
| 73 | Andrews, N. P. (1932) (disc.) | F. | As. Psor. | Nothing noted | Mult. superf. ep. (including hands) | . . |

TABLE I (*continued*).

| No. | Author. | Sex. Age. | Drug. Disease. | Keratoses. Pigmentation. | Localization of epitheliomas. | Biopsy. Metastases. Death. |
|---|---|---|---|---|---|---|
| 74 | Anderson, N. P. (1932), "case 1" (picture) (see Montgomery, 1935, p. 229) | M. 75 | S. Fow. 60 y. ago over unknown period | Ker. p. and s., back of hands, chest. Cutaneous horn, groin. Seborrhoic keratoses | Numerous les. lower part of r. leg and foot, begin. ab. 20 y. ago (?). Mult. superf. ben. ep. back, buttock (Bowen's dis. or arsenic type of ep. ?) | .. |
| 75 | Anderson, N. P. (1932), "case 3" | M. 70 | As. 17 or 18 y. ago as tonic (nerv. breakdown) | Ker. p. and s.; s. 2 y. Seborrhoic keratoses | One lesion of ben. superf. ep. forehead s. 5 or 6 y. | Bas. c. carc. |
| 76 | MacCormac, H. (1933*a* and *b*) | F. 51 | As. (?) 25 y. ago f. 2 y. Asthma | Ker. p. and s. (punctate). No pig. | Mult. superf. ep. trunk and premalignant warty nodules arms, waist, abdomen. Ca. cervicis uteri | Bas. c. carc. |
| 77 | Barber (1933) (discussion to MacCormac, 1933*b*) | M. | As. large doses 20 y. prev. long time. Dermatitis herpetif. | Ker. p. and s. punctate. Melanosis | Mult. superf. ep. | Bas. c. carc. |
| 78 | Guggenheim, L. (1933) | F. 55 | S. Fow. f. 27 y., 5 drops 2 *t.d.* (25 g. in 27 y.). Epilepsy | Ker. p. and s. Melanosis | Mult. (13) les., polymorph, beg. 15 y. aft. start As.; not on p. and s. Bowen-like | 1 Cancroid, 1 bas. c. carc., atyp. |
| 79 | Wright and Friedman (1933), "case 2" (picture) | M. 45 | Drops, app. As. 15 y. prev. f. 6 weeks. Psor. | No ker. No pig. | Twelve superficial ep. Seven back, first s. 9 y. Three: l. arm One: chest One: l. temple | Mixed bas. and sq. c. carc. |
| 80 | Wright and Friedman (1933), "case 4" | F. 40 | As. over 20 y. ago f. 2 y. Psor. | No ker. No pig. | Mult. superf. ep. w. pearly border One: forehead Two: back | .. |
| 81 | Wright and Friedman (1933), "case 5" | F. 60 | As. 20 y. prev. f. 1 y. Psor. | No ker. No melanos. | Three superf. ep.: One: leg, s. 13 y. One: neck One: chest | .. |
| 82 | McNeer (1934), "case 1" | M. 31 | S. Fow. 22 y. ago, 30 drops d. f. 6 m. | Ker. | Many les. beg. 5 y. ago nr. anus and other parts | Sq. c. carc. |
| 83 | McNeer (1934), "case 2" | M. 50 | S. Fow. 20 y. ago, 5 drops 3 *t.d.* f. 3 weeks. Psor. | Ker. hands and extr. trunk | Numerous lesions: hands, upp. extr., trunk, beg. 9 y. ago | Sq. c. carc. |
| 84 | McNeer (1934), "case 3" | M. 37 | As., beg. 17 y. ago | Ker. p. and s., dors. of hands and feet | One: r. thumb, on wart 8 y. after start. As. | Metas. |
| 85 | Ayres, S., Jr. (1934) | M. over 60 | S. Fow. Eczema (as student) | Ker. p. and s. (warty) and on trunk beg. 19 y. ago. Telangiect. | One: r. foot on ker. Four: l. heel Mult. ben. superf. ep. w. pearly border, trunk, arms, legs, plus Bowen's dis. (?) | .. |
| 86 | Wilhelm (disc. Ayres, 1934) | M. | S. Fow. f. sev. y. Epilepsy | Ker. p. | Superf. ep. trunk | .. |
| 87 | Franseen and Taylor (1934), "case 2" | M. 55 | As. f. 3–4 y. and arsphenamine. Dermatitis of scalp | Ker. p., hands, feet, trunk | Mult. epidermoid carc., app. on ker. l. index, l. thumb, r. foot, back, chest, shoulder, heel | Sq. c. carc. Death. |
| 88 | Franseen and Taylor (1934), "case 4" | F. 67 | S. Fow. 40 y. prev. for constitution | Ker. p., arm, trunk | One: ov. cr. ilei, on ker. s. 7 y. One: neck, papillary. One: r. thigh Susp. carc. oesophagi | Sq. c. carc. |

TABLE I (*continued*).

| No. | Author. | Sex. Age. | Drug. Disease. | Keratoses. Pigmentation. | Localization of epitheliomas. | Biopsy. Metastases. Death. |
|---|---|---|---|---|---|---|
| 89 | Franseen and Taylor (1934), "case 5" | M. 63 | S. Fow. 10–15 y. prev., 5 drops *t.i.d.* for 2 y. Psor. | Ker. p. | 27 lesions, back, trunk, thigh<br>One : l. shoulder | Sq. c. carc. |
| 90 | Franseen and Taylor (1934), "case 6" | F. 50 | "Dr. Greene's Nervine" 29 y. ago. Nervousness, epilepsy | Ker. whole body (incl. mons pubis) | One : l. middle finger and l. index (papillary)<br>One : neck on ker.<br>One : l. foot (suspic.) | Sq. c. carc. Metas. Death. |
| 91 | Franseen and Taylor (1934), "case 7" | M. 51 | As. 2 m. | Mult. ker. whole body, hands and feet | One : l. groin | Sq. c. carc. |
| 92 | Franseen and Taylor (1934), "case 8" | M. 53 | As. 15 y., 6 drops 3 *t.d.* Skin dis. | Ker. p. and s., hands, warty face | One : l. thumb on ker.<br>One : l. ring finger | Mixed bas. sq. carc. |
| 93 | Franseen and Taylor (1934), "case 9" | M. 47 | S. Fow. quite a period. Epilepsy | Ker. p. and s. shortly after stopp. As. | Carc. pancreat. | .. |
| 94 | Franseen and Taylor (1934), "case 10" (picture) | M. 37 | S. Fow. beg. 14 y. ago, up to 15 drops 3 *t.d.* f. 10 y. w. interv. Psor. (hered.) | Ker. p. and s. beg. 8 y. after starting As. | One : back of hand, beg. 13 y. after starting As. | Sq. c. carc. |
| 95 | Franseen and Taylor (1934), "case 16" | M. 34 | S. Fow. Asthma | Two ker. temp. reg. | One over the first thoracic vert. | Bas. c. carc. |
| 96 | Franseen and Taylor (1934), "case 17" | F. 53 | As. 32 y. prev. f. 3 y. off and on. 10 y. prev. f. 2–3 months. Psor. | Ker. trunk, no ker. p. and s. | Beg. age of 51<br>One : nose<br>One : forehead<br>One : l. temp. reg<br>One : r. upp. arm<br>One : l. upp. arm<br>Two : hip | Ep. aden.-cystic.<br>Bas. c. carc. Metas. Death. |
| 97 | Franseen and Taylor (1934), "case 19" | M. 53 | S. Fow. in boyhood f. a short t. Acne ? | No ker. p. and s. | One : back, with pearly border<br>Sev. : back<br>One : ulc. above nipple | .. |
| 98 | Waugh, J., and Scull (1935) | F. 58 | S. Fow. 5–7 drops 3 *t.d.* f. more than 3 y. Ataxia ? | Ker. p. and s. punctate. Pig. | Mult. (ab. 12) superfic. ep. on the body, esp. reg. iliaca | Bas. c. carc. |
| 99 | Parkhurst, H. J. (disc. to Waugh and Scull, 1935), "case 1" | .. | As. from time to time. Psor. | Ker. hands | Superf. epitheliomatosis widespread | .. |
| 100 | Parkhurst, H. J. (*ib.*), "case 2" | .. | As. Psor. | Ker. hands | Superf. ep. | .. |
| 101 | Robinson, S. (1935) | .. | S. Fow. s. 12 y. Psor. | Ker. p. and s., punctate | One : nose | Bas. c. carc. |
| 102 | Montgomery, H. (1935), "case 1" | M. 34 | S. Fow. beg. 10 y. ago f. 5 y., 1–26 drops 3 *t.d.*, total 1200–1450 c.c. Psor. | Ker. p. and s. s. 7 y. Pig. | One : sole papillom.<br>One : l. foot on ker.<br>Sev. : l. palm<br>One : nr. anus<br>One : scrotum | Sq. c. carc. Metas.<br>Sq. c. carc.<br>Sq. c. carc.<br><br>Sq. c. carc. |
| 103 | Montgomery, H. (*ib.*), "case 2" | M. 42 | S. Fow. in early childhood f. 6 y. Eczema | Ker. | One : l. s. fr. ker. 8 y. ago<br>7 y. later : num. superf. ep. on trunk,<br>and one superf. ep.<br>Later : intraur. ep.<br>Later : bronchial carc. | Sq. c. carc.<br>Sq. c. Bowen<br>Bas. c. carc.<br><br>Sq. c. carc.<br>Death. |

TABLE I (*continued*).

| No. | Author. | Sex. Age. | Drug. Disease. | Keratoses. Pigmentation. | Localization of epitheliomas. | Biopsy. Metastases. Death. |
|---|---|---|---|---|---|---|
| 104 | Montgomery (1935), " case 4 " | M. 63 | " Ascato " 30 y. prev. Asthma | Ker. p., fingers, 20 y. after start. As. No pigment. Verruc. senil. | Several p. and fingers, some on ker. s. more than 6 y. One : scalp More than 50 superf. ep. trunk " arsenical type " 6–7 y. | Sq. c. carc. One : bas. c. ep. changing in sq. |
| 105 | Montgomery (1935), " case 5 " | F. 44 | S. Fow. 30 y. ago, 3–16 drops 3 *t.d.* f. more than 1 y. Chorea | *Nil* noted | One : r. axilla 15 y. " arsenical type simulating Bowen " | . . |
| 106 | Montgomery (1935), " case 6 " | M. 50 | S. Fow. as a child. Chorea | Ker. p. and s. Slight pig. | Num. superf. ep. One : a typical verruc. lesion | . . |
| 107 | Trow (disc. to Montgomery, 1935) | M. 50 | S. Fow. f. 2–3 y. Fits | Ker. p. | One : trunk and back Two : back of fingers app. on ker. | . . |
| 108 | Rosen (disc. to Montgomery, 1935) | M. 40 | Arsphenamine f. ulc. in the mouth | . . | Oral mucosa | . . |
| 109 | Alderson (1935) | M. 50 | As. pills, 15 ! daily, each cont. 0·1–0·4 mg., beg. 27 y. ago, f. 7 y. Psor. | Ker. p. and s. (corns). Ker. trunk, nose, ear, eyelid. Pig. | Mult. polymorph. plaques over the body One : abdom., superf., later malign. One : fourth finger later malign. One : neck | . . |
| 110 | Ayres, S., Jr. (1935) | F. 59 | S. Fow. 49 y. ago over a long per. Psor. (?) | Ker. p. and s. (punct.) | Superf. ep. s. 12 y. Two (or more ?) : face, forehead, neck, scalp; sim. Bowen | . . |
| 111 | Bloom (1936) | F. | As. Psor. | Ker. p. and s. No pig. | Superf. ep. back Ep. p. and s. on ker. | . . Sq. c. carc. |
| 112 | Fassrainer (1936) (see also Straaten, 1935) | M. 35 | S. Fow. 1½ y. Eczema, hands | Ker. hands (warts) | One : hand, from a wart | Bas. c. carc. Metas. |
| 113 | Anderson (1937) | M. | " Ascato " 2–3 y. Asthma | Ker. p. and s. | One : l. index 5 y. later : r. index beg. as a wart Mult. ben. superf. ep. scattered over trunk and upp. port. of arms | Sq. c. carc. |
| 114 | Ayres, S., Jr. (disc. to Anderson, 1937) | . . | As. | Arsenical ker. | One : palm One : axill. | Bas. c. carc. Bas. c. carc. Metas. (sq. c. ep.) |
| 115 | Traub (1937) | F. 68 | As. 40 y. ago f. 4 y. Acne, psor. | Ker. (?) leg | Mult. polymorph. les. trunk, leg, face beg. 4 y. after medic. of As. One : back | Bas. c. carc. Sq. c. carc. |
| 116 | Robinson (1937) | M. | S. Fow. f. many y. Psor. | Ker. p. and s. and body s. 1½ y. Pig. | One : l. sole (ulcus). Pigmented macular scaling areas over the body | . . |
| 117 | Rauschkolb (Arday) (1938) | M. 49 | S. Fow. f. 25 y. off and on. Psor. | Ker. p. and s. Telangiect. | About 50 superf. ep. trunk, upp. extr. beg. 8 y. ago | Bas. c. carc. |
| 118 | Barber (1939) | M. 37 | As. f. 10 y. Epilepsy | Ker. p. and s. | Numer. superf. ep. trunk | Bas. c. carc. |
| 119 | Haldin-Davis (1939), disc. to Barber (1939) | F. | As. f. 20 y. Epilepsy | Ker. p. and s., etc. Pig. | At least one : abdomen | . . |
| 120 | Voss (1939) | F. 62 | S. Fow. As. pills 20 y. ago. Psor. | Ker. trunk but not p. and s. Pig. | 18 polymorph. les. trunk, reg. genit. rima ani, thigh, ab. 7 y. | Bas. c. carc. |

TABLE I (*continued*).

| No. | Author. | Sex. Age. | Drug. Disease. | Keratoses. Pigmentation. | Localization of epitheliomas. | Biopsy. Metastases. Death. |
|---|---|---|---|---|---|---|
| 121 | Ryan (1939) | M. 52 | As. over 12 y. Stomach trouble | Ker. p. and s. and scatt. of tr. and extr. s. 16 y. | Mult. superf. ep. (?)<br>One : r. ear<br>One : chest, trunk (Bowen-like) | . . |
| 122 | Goeckerman and Wilhelm (1940) | M. 70 | S. Fow. f. 10 y. Epilepsy | Ker. p. and s. 14 y. after starting As. 2 y. later trunk (Bowen-like) | Papilloma ureteris et vesicae | . . |
| 123 | Anderson (disc. to Goeckerman and Wilhelm, 1940) | M. 21 | As. 7 y. prev. f. 6 m. | Ker. p. and s. | Mult. superf. ep. trunk | Sq. c. carc. |
| | | | | | One large fungating in groin<br>Carc. of tongue | |
| 124 | Applestein (1941) | F. 60 | S. Fow. beg. 35 y. ago; f. 25 y. 5–10 drops 3 *t.d.* Psor. | Nothing noted ab. ker. Pig. Telangiect. | Mult. : epitheliomatosis<br>One : l. shoulder s. 8 y. | |
| | | | | | One : r. temple | Bas. c. carc. |
| | | | | | One : chest, papill., prob. not malign.<br>Forehead, thighs, r. elbow, scalp | |
| 125 | Rodgers (1941) | M. 55 | S. Fow. many y. ago | Nothing noted ab. ker. | Mult. ben. superf. ep. s. 4 y. | |
| | | | | | L. shoulder | Sq. c. carc. |
| | | | | | Little finger | Bowenoid ? |
| | | | | | Many : back of hands, scalp, r. knee and legs | Bas. c. carc. |
| | | | | | Back (cauliflower-like) | Mixed c. carc. |
| 126 | Robinson (disc. to Rodgers, 1941) | . . | As. (?) | Arsenical ker. finger, etc. | One : finger | Sq. c. carc. |
| 127 | Montgomery and Waisman (1941), " case 1 " | M. 49 | S. Fow. 26 y. at least once yearly until 9 y. ago. Psor. | Ker. p. and s. s. 14 y. | Sev. : l. p. on ker. (thumb), 15 months ago | Sq. c. carc. |
| | | | | | Numerous trunk and upp. extr. | Sq. c. carc., ars. type; some Bas. c. carc. |
| | | | | | One : r. cheek | Bas. c. carc. |
| | | | | | One : r. fossa, nasal | Bas. c. carc. |
| | | | | | One : r. temple, papillomat. w. pearly border | |
| 128 | Montgomery and Waisman (1941), " case 2 " | F. 69 | S. Fow. 20 y. ago f. 5 weeks and 1 y. ago for 1 week | Num. ker. trunk and extr. (nothing noted of p. and s.). No pig. | Num. lesions trunk and extr. s. 2 y. | Sq. c. carc. (Bowen-like). |
| | | | | | One verruc. les. on little finger s. 5 y. | Sq. c. carc. |
| 129 | Montgomery, R. M. (1942) | F. 42 | S. Fow. 30 y. ago. Acne | Ker. fingers, soles (punctat.). Leucopl. oris | Multiple : dors. of r. foot, l. knee, l. thigh, dors. of l. hand, wrists s. 10 y. | Bowen dis. (biopsy). |
| 130 | Peck (1942) | M. 45 | As. drops 3½ y. Psor. hered. | Ker. p. | Two : l. p. on ker. | Sq. c. carc. |
| 131 | Ayres, S., Jr. (1943) | M. 56 | Asiat. pills 33 y. ago f. 5 y. Psor. | Ker. p. and s. (punctate) | Ab. 30 ben. superf. ep. polym orph., trunk, r. rm. f. 8 y. | Some : sq. c. carc. |
| 132 | Anderson (1943) | F. 47 | As. inject. 20 y. prev. | Ker. s., no p. | Dry scaly lesions trunk, s 3–4 y. | |
| | | | | | L. shoulder | Bas. c. carc. |
| | | | | | L. hip | |
| 133 | Ebert and Otsuka (1943) | F. 42 | As. as child Anaemia | . . | One : r. hip s. 14 y. | Bowen d. or intraepid. ep. |
| | | | | | One : l. chest | |

TABLE I (*continued*).

| No. | Author. | Sex. Age. | Drug. Disease. | Keratoses. Pigmentation. | Localization of epitheliomas. | Biopsy. Metastases. Death. |
|---|---|---|---|---|---|---|
| 134 | Anderson (1943) | M. 50 | As. | Ker. p. and s. (punctate) | One: superf. ep. r. shoulder<br>Later: two, scrotum | Bowenoid, Pagetoid. |
| 135 | Wilhelm and Goeckerman (1943) | M. 72 | As. drops and inject. 10 y. ago | No ker. Pig. | Mult. ben. superf. ep. trunk | Probably bas. c. carc. |
| 136 | Laymon (1943) | F. 61 | As. drops 17–22 (?) y. prev. f. 5 y. Anaem. pernic. | Ker. p. and s. | At least 30 superf. ep. trunk | . . |
| 137 | Hall (1944) | F. 61 | As. drops (?) small quant. 45 y. ago f. short time (only 7·5c.c.) f. complexion. Later As. for syph. orally and i.v. | No ker. p. and s. | Several superf. ep. s. 11 y.<br>One: scap. reg. | . . |
| 138 | Barney (1944) | M. 46 | Black pills 20 y. ago intermitt. f. 6 months. Psor. | Ker. p. and s. one y. later. Ker. trunk | One: beg. in patch of psor. 10 y. ago<br>Num. superf. polymorph. ep. trunk, extrem.<br>One: below axill.<br>One: scrotum<br>Later: carc. oesophag. | <br><br>Bas. c. carc.<br><br>Sq. c. carc. |
| 139 | Dowling (disc. to Glen, 1945) | Old | As. years before. Psor. | Nothing noted ab. ker. | Numerous flat ep. | Sq. c. carc. ? Death, general carc. |
| 140 | Russell and Klaber (1945), "case 1" | F. 53 | S. Fow. 3 drops 3 *t.d.* beg. 13 y. ago; total 37,000 drops. Epilepsy | No ker. p. and s. Pigment. (no "raindrop"). Mult. seborrh. warts, mollus. | Polymorphic lesions<br>One indurated ulcer chest 12 y. after starting As.<br>Two plaques with pearly edge over sternum | <br>Sq. c. carc. |
| 141 | Russell and Klaber (1945), "case 2" | F. 44 | S. Fow. in Br-mixt. f. 30 y.; total 79,000 drops—700 gr. $A_2sO_3$. Epilepsy | Ker. p. No raindrop-pigment. Mult. seborrh. warts and mollus. fibros. | Mult. ep. w. pearly rolled edges trunk, shoulder, thighs<br>Indurated plaque r. f. iliaca<br>One: r. nipple | <br><br><br>Bas. c. carc. (cyst. deg.). |
| 142 | Semon, H. C. (disc. to Russell and Klaber, 1945) | M. | S. Fow. large dos. up to 1 dr. 3 *t.d.* Dermatitis herpetiformis | . . | Carc. bronch. (skin not affected) | . . |
| 143 | Semon, H. C. (*ib.*), "case 2" | . . | As. for 14 y. Psor. | Ker. feet | One: l. foot on ker. | Sq. c. carc. Death. |

### TABLE II.

| | Keratoseogen except comb. w. mult. superf. (53 cases). | Mult. superf. and Bowen except comb. w. kerat. (48 cases). | Combination of keratoseogen and mult. superf. (13 cases). | Epith. on patches of psor. (7 cases). | All cases (136 cases). |
|---|---|---|---|---|---|
| Fingers, hand, wrist, toes, foot, ankle | 45 = 84·9% | 4 = 8·3% | 13 = 100% | .. | 63 = 46·3% |
| Arm, thigh, leg | 6 = 11·1% | 21 = 43·7% | 4 = 30·8% | 6 = 85·7% | 41 = 30·1% |
| Scrotum | 3 = 5·7% | 1 = 2·1% | .. | 2 = 28·6% | 6 = 4·4% |
| Penis | 1 = 1·9% | .. | .. | .. | 1 = 0·7% |
| Vulva | 1 = 1·9% | 1 = 2·1% | .. | .. | 2 = 1·5% |
| Pubes, groin, perineum, anal reg. | 3 = 5·7% | 3 = 6·25% | 1 = 7·7% | 3 = 42·9% | 11 = 8·9% |
| Neck | 5 = 7·5% | 4 = 8·3% | .. | .. | 8 = 5·9% |
| Head | 3 = 5·7% | 13 = 27·1% | 1 = 7·7% | .. | 19 = 14·0% |
| Trunk, icl. shoulder, axill., breast, hip, buttock, sacr. reg. | 8 = 15·1% | 40 = 83·3% | 10 = 76·9% | 1 = 14·43% | 63 = 46·3% |
| "All over the body" | 1 = 1·9% | 3 = 6·25% | 2 = 15·4% | .. | 7 = 5·1% |
| Extracutan. | 3 = 5·7% | 2 = 4·2% | 1 = 7·7% | .. | 12 = 9·6% |

In this Table different localizations in the same region are counted only as one even if, e.g., both hands and both feet are affected.

### TABLE III.

| | All cases. | Males. | Females. |
|---|---|---|---|
| 20–30 years | 10 cases = 8·7% | 5 cases = 6·25% | 5 cases = 14·3% |
| 31–40 „ | 28 „ = 24·3% | 19 „ = 23·75% | 9 „ = 25·5% |
| 41–50 „ | 35 „ = 30·4% | 25 „ = 31·2% | 10 „ = 28·6% |
| 51–60 „ | 24 „ = 20·9% | 16 „ = 20·0% | 8 „ = 22·9% |
| 61–70 „ | 17 „ = 14·8% | 14 „ = 17·5% | 3 „ = 8·6% |
| 71–80 „ | 1 case = 0·9% | 1 case = 1·25% | .. |
| | 115 cases | 80 cases | 35 cases |

### TABLE IV.

| | |
|---|---|
| Hamilton (1921, case 38) | Traces |
| Schwartz (1926, case 49) | 0 |
| Anderson (1932, case 24) | 0·00025 mg.% |
| „ ( „ „ 25) | 0·007 mg.% |
| Ayres, Jr. (1934, case 85) | 0·0027–0·0048 mg.% (in dried substance) |
| Montgomery (1935, case 97) | 0·035 mg.% |
| „ ( „ „ 99) | 0 |
| Anderson (1937, case 113) | 0·037 mg.% |
| Montgomery and Waisman (1941, case 122) | 0 |
| „ „ ( „ „ 128) | 0 |

### TABLE V.

| | $As_2O_3$ in growth. | | $As_2O_3$ in normal skin. | |
|---|---|---|---|---|
| | Chem. | Osborne. | Chem. | Osborne. |
| Case 102, keratoseogen ep. (sq.) | 0 | + | .. | .. |
| „ 103, „ „ (sq.) | 0 | + | 0 | 0 |
| „ 104, mult. ep. as. type | | | | |
| (*a*) hand (sq. ep.) | .. | ++ | .. | + |
| (*b*) trunk (sq. ep.) | .. | ++ | .. | .. |
| (*c*) back (sq. ep.) | 8, 6 mg. % | + | .. | .. |
| „ 105, mult. ep. as. type (sq.) | 0·18 mg. % | ++ | .. | + |
| Case of superf. ep. (1935, p. 232) (bas. c.) | 0 | + | 0 | + |
| Ditto (bas. sq.) | 0 | + | 0 | + |
| Ditto (Bowen ) | 0 | 0 | 0 | 0 |
| Case 106, ben. superf. ep. | | | | |
| (*a*) bas. c. ep. | 0·5 mg. % | .. | 0·77 mg. % | .. |
| (*b*) Verrucous les. (sq. c.) | .. | ++ | .. | + |
| Case 127, superf. ep. as. type | | | | |
| (*a*) several (sq. and bas. c.) | 0.024–430 mg. % | .. | 0·008 mg. % | .. |
| (*b*) thenar (sq. ep.) | .. | ++ | .. | + |
| Case 128, num. les. as. type trunk, arms (sq. ep.) | 2·2–27·5 mg. % | + | 100 mg. % (?) | .. |

TABLE VI.—*Occupational Arsenical.*

| Case. | Author. | Sex. Age. | Employment. | Keratoses. Pigmentation. | Localization of epithelioma. | Biopsy. Metastases. Death. |
|---|---|---|---|---|---|---|
| MINERS, SMELTERS : | | | | | | |
| 1 | Anderson (1932), "case 2," fig. 3. (See also Ayres and Anderson, 1934) | M. 51 | 34 y. previous in smelters in Mexico for 8 y. | Ker. p. and s. | Mult. ben. superf. ep. chest and back s. 7 years | Bas. c. carc. and dyskeratoses. |
| 2 | Schaerrer (1934) | .. | In silver mining or smelting | .. | .. | Sq. c. carc. Metas. |
| 3 | Migavi (1935) | .. | 16 y. in smeltery in Japan | Ker. p. and s. 10 y. after starting work | Sq. c. ep. Metas. | .. |
| 4 | Andrews (disc. to Goeckerman and Wilhelm, 1940) | M. | In a copper mine | Many typical ker. p. and s. | Epitheliomas | .. |
| SHEEP-DIP WORKERS : | | | | | | |
| 5 | Eve (1913). (See also O'Donovan, 1924, "case 2") | M. 40 | 20 y. in sheep-dip fact. Pig. of entire skin | Ker. p. (not s.) | One over l. clav. 2 y. | Sq. c. carc. |
| 6 | Porter (1913). (See also O'Donovan, 1924, "case 3") | M. 35 | 20 y. in sheep-dip fact. | Pig. all over the body | One : l. shoulder beg. as a wart 8 months prev. | Sq. c. carc. Death. |
| 7 | Legge (1923), "case 1" | M. 53 | 38 y. in sheep-dip fact. | Warty ker. | One abdomen | Carc. not sure. |
| 8 | Legge (1923), "case 2" | M. 53 | 33 y. in sheep-dip fact. | Ker. p. | Mult. growths armpit, groin, thigh, scrotum, sacrum, arms, feet | .. |
| 9 | Legge (1923), "case 3" | M. 39 | 22 y. in sheep-dip fact. | Ker. p. | Warty conditions back of hands, r. groin, l. buttock | Carc. not sure. |
| 10 | Legge (1924, 1934), "case 4" O'Donovan (1924*a*, 1928), "case 1" (picture) | M. 44 | 26 y. in sheep-dip fact. | Ker. warts | One : l. temple on a wart. Four growths prev., l. clavicle, abdomen, l. flank, thigh | Sq. c. carc. |
| 11 | Bridge (1926) | .. | .. | .. | .. | Metas. death. |
| 12 | Bridge (1926) | .. | .. | .. | .. | Metas. death. |
| 13 | Bridge (1930) | M. 61 | 46 y. in sheep-dip fact. | .. | Subsequent primary growths :— One : r. shoulder (36 y. after beg. employm.) One : neck (5 y. later) One : l. upper arm (5 y. later) One : penis | .. |
| 14 | Legge (1934), "case 3" | M. 49 | 22 y. in sheep-dip fact. | Ker. p. Pig. | Warts back of hands, r. groin, l. buttock | .. |
| 15 | Merewether (1943) | M. 57 | 43 y. in sheep-dip fact. | .. | .. | .. |
| 16–18 | Merewether (1943) | .. | In sheep-dip fact. | .. | .. | .. |
| INSECTICIDES : | | | | | | |
| 19 | Legge (1925) Bridge (1928) | M. 48 | 19 y. in emerald green fact. and 10 y. further exposure to As. | Pig. | One : l. axilla. 2 y. later, one below l. knee | Metas. death. |

TABLE VI.—*Occupational Arsenical* (*continued*).

| Case. | Author. | Sex. Age. | Employment. | Keratoses. Pigmentation. | Localization of epithelioma. | Biopsy. Metastases. Death. |
|---|---|---|---|---|---|---|
| 20 | Hopkins and Van Studdiford (1934) (picture) | M. 5 Negro | Living in cotton wool farm; fields sprayed with insecticides | Ker. p. Pig. hands. | Mult. ep. face s. 4 y., exulcerated | .. |
| 21 | Franseen and Taylor (1934) "case 1" | M. 59 | 30 or more y. employed w. As-prep. f. fruit trees | Ker. p. a. s. | Mult. scaly les. ov. the body s. 40 y. 4 bas. c. ep. ear, upp. lip, arm, leg | Resid. |
| 22 | Franseen and Taylor (1934) "case 3" | M. 51 | 27 y. ago empl. w. As-prep. for fruit trees | Ker. p. warty | One carc. neck. Mult. carc. trunk | .. |
| 23 | v. Pein (1938*a*, *b*) | M. 40 | Vintager, 4 y. use of " Nospran " | Ker. p. a. s. | Mult. ulc. carc. skin | .. |
| 24 | Bridge (1939) | | Empl. in factory of As-containing insecticides | Melanosis, Perfor. of nasal sept. | Warty growths ov. the body. Primary carc. of r. lung | .. |

ABBREVIATIONS.

As., arsenic ; bas., basal ; beg., beginning ; betw., between ; carc., carcinoma ; c., cell(ed) ; ep., epithelioma ; interv., interval ; ker., keratosis ; l., left ; les., lesion(s) ; m., month ; metas., metastases ; mult., multiple ; nr., near ; num., numerous ; p., palms ; per., period ; pig., pigment(ed) ; psor., psoriasis ; r., right ; rec., recurrence ; s., soles ; S. Don., Sol. Donovan ; S. Fow., Sol. Fowleri ; sev., several ; sq., squamous ; superf., superficial ; ulc., ulcer(ated) ; y., years.

cancer in Table I is the occurrence of malignancy in other members of the family mentioned (cases 4 and 104, in neither of them affecting the skin). Such occasional coincidence of course cannot prove anything. On the other hand a systematic investigation of the hereditary conditions in arsenical cancer has never been carried out.

The possible role of *embryonic germs* in the sense of the Cohnheim theory of cancer was the starting-point of the above-mentioned experiments of Carrel (1925) and Askanazy (1926).

Askanazy, stressing the necessity to assume the *co-operation of several factors* for the genesis of arsenical cancer, ascribed to arsenic the role of an irritant of oligodynamic efficiency, acting alongside a general constitutional predisposition (" allgemeine Koerperbereitschaft ") and a local predisposition (e.g. presence of embryonic tissue). Fischer-Wasels (1928, 1929) and Buengeler (1930), on the other hand, suggested that arsenical poisoning after a latent sensitizing period causes a general disposition for cancer (" allgemeine Geschwulstdisposition "), marked by certain metabolic changes (see p. 228), preparing the ground for the development of malignancy out of a local anlage (" locale Geschwulstanlage ").

Given such a general disposition, or provided even pre-cancerous lesions (keratosis) have developed, then the subsequent development of an epithelioma at that site may be facilitated by the action of *non-specific external irritation*, such as by rubbing, scratching, or other slight traumatism (Wile, 1912; de Silva, 1918).

Eggers (1931), summarizing the facts, concludes that arsenic is an agent that seems to cause a decided increase in the predisposition to cancer, so that an added element of irritation, which in ordinary conditions would be inadequate, comes into operation.

This work was carried out with the aid of a grant from the Medical Research Council.

### REFERENCES.

Abramowitz, E. W., Mattice, M. R., and Bottvinck.—(1944) *Arch. Derm. Syph., Chicago*, **47**, 175.

Aitken, R.—(1944) *Edinb. med. J.*, **51**, 339.

Alderson, H. E.—(1935) *Arch. Derm. Syph., Chicago*, **32**, 313.

Alexander, A.—(1921) *Arch. Derm. Syph.*, Wien, **129**, 5.

Aliferis.—(1924) *Ibid.*, **147**, 349.

Althausen and Gunther.—(1929) *J. Amer. med. Ass.*, **92**, 2002.

Alvarez, C.—(1928) *Rev. méd. Rosario*, **18**, 171.

*Idem* and Ruiz, E. R.—(1927) *Ibid.*, **17**, 115.

Anderson, N. P.—(1932) *Arch. Derm. Syph., Chicago*, **26**, 1052.—(1937) *Ibid.*, **35**, 323.—(1940) *Ibid.*, **42**, 641.—(1943*a*) *Ibid.*, **47**, 136.—(1943*b*) *Ibid.*, **48**, 319.

*Idem* and Burpeau, C.—(1945) *Ibid.*, **52**, 46.

Andrews, N. P.—(1932) *Ibid.*, **25**, 572.

Antonoff, A. M.—(1928) 'Problems of Oncology,' Kharkoff, VI, 292. Ref. in *Cancer Rev.* (1930), 108.

Applestein, R.—(1941) *Arch. Derm. Syph., Chicago*, **43**, 599.

Arday, E. J.—(1938) *Ibid.*, **37**, 331.

Arguello, R. A., Cenget, D., and Tello, E. E.—(1938) *Rev. Derm. Argent.*, **22**, 461; ref. in *Brit. J. Derm.* (1939), **51**, 548.

*Idem*, Ferraris, L. V., and Tello, E. E.—(1938) *Rev. Derm. Argent.*, **26**, 313.

*Idem* and Tello, E. E.—(1943) *Ibid.*, **27**, 152.

*Iidem* and Marcola, B.—(1942) *Ibid.*, **26**, 1155.

Arning.—(1922) *Arch. Derm. Syph., Wien*, **138**, 458.

Arnstein, A.—(1913*a*) *Verh. deutsch. path. Ges.*, **16**, 332.—(1913*b*) *Oesterr Sanitaetswesen*, 38, *Wien Arb. soz. Med.*, Beihefte **5**, 64.

Aschoff, L.—(1887–1889) in 'Klin. Jahrbuch,' 1902.

Askanazy, M.—(1926) *Verh. dtsch. path. Ges.*, **21**, 182.

Ayres, S., Jr.—(1934) *Arch. Derm. Syph., Chicago*, **29**, 613.—(1935) *Ibid.*, **32**, 322.—(1937) *Ibid.*, **35**, 323 (discussion).—(1943) *Ibid.*, **47**, 135, 136.

*Idem* and Anderson, N. P.—(1934) *Ibid.*, **30**, 33.—(1938) *J. Amer. med. Assoc.*, **110**, 886.

Balthazard.—Arsenic in 'Occupation and Health Encyclop.,' Geneva, 1930, vol. i, p. 159.

Banga, I., Schneider, L., and Szent-Gyeorgyi, A.—(1931) *Biochem. Z.*, **240**, 462.

Barber, H. W.—(1925) *Brit. J. Derm.*, **37**, 37.—(1928) *Ibid.*, **38**, 294.—(1933) *Ibid.*, **45**, 475 (discussion).—(1939) *Ibid.*, **58**, 85.

Barney.—(1944) *Arch. Derm. Syph., Chicago*, **50**, 339.

Barry, G., Bunbury, E., and Kennaway, E. L.—(1928) *Biochem. J.*, **22**, 1109.

Bayet.—(1923*a*) *Le Cancer*, **1**, 5.—(1923*b*) *Congr. du Cancer, Strasbourg*, **2**, 15.—(1930) 'Occupation and Health Encyclop.', vol. ii, p. 1140.

*Idem* and Slosse.—(1919*a*) *Bull. Acad. Méd. Belg.*, **29**, 607.—(1919*b*) *C.R. Acad. Sci.*, **168**, 704.—(1919*c*) *C.R. Soc. Biol.*, **82**, 1144.

Begg, A. M., and Cramer, W.—(1929) *Lancet*, ii, 217, 697.
Berenblum.—(1932) *Cancer Rev.*, **7**, 338.
Bierich, R.—(1922) *Z. Krebsforsch.*, **18**, 226, *cit.* Lipschuetz, 1924.
Billeter, O., and Marfurt, E.—(1923) *Helv. chim. Acta*, **6**, 780.
Binz, C., and Schulz, H.—(1879) *Arch. exp. Path. Pharmak.*, **11**, 200.
Bland-Sutton, J.—(1916) *Brit. med. J.*, ii, 788.
Bloom, D.—(1936) *Arch. Derm. Syph., Chicago*, **33**, 937.
Bosco, J. J.—(1925) *Semana méd.*, **32**, ii, 951.
Bosellini.—(1928) *Brit. J. Derm.*, **38**, 48.
Bowen, J. T.—(1912) *J. cutan. Dis.*, **30**, 241.
Bridge, J. C.—(1926) *Ann. Rep. Chief Ins. Fact. and Worksh.*—(1928) *Ibid.*—(1930) *Ibid.*—(1939) *Ibid.*
Brooke, H. G., and Roberts, L.—(1901) *Brit. J. Derm.*, **13**, 121.
Bruenauer.—(1921) *Arch. Derm. Syph., Wien*, **129**, 186.
Buengeler, W.—(1930) *Frankf. Z. Path.*, **39**, 314.
Buergener, J.—(1939) *Dermatolog.*, **80**, 86.
Butlin, H.—*Brit. med. J.*, i, 1341 ; ii, 1, 66.
Califano, L.—(1931) *Riv. Patol. sper.*, **6**, 113.
Carlson, C. E.—(1910) *Z. physiol. Chem.*, **68**, 243.
Carrel, A.—(1925) *C.R. Soc. Biol.*, **93**, 1083.
Cartaz—(1878) *Bull. Soc. anat., Paris*, **9**, 587.
Cholewa, J.—(1934/35) *Z. Krebsforsch.*, **41**, 497.
Ciechanowski, Morozowa, F., and Wilhelmi, M.—(1925) *Polska Gaz. lekarska*, 3, **25**, 305 ; ref. *Zbl. allg. Path. path. Anat.*, **36**, 294.
Cole and Driver.—(1929) *Arch. Derm. Syph., Chicago*, **20**, 143.
Collier, W. A., and Hartnack.—(1929) *Z. Krebsforsch.*, Orig., **30**, 131.
Cook, J. W., Haslewood, G. A. D., Hewett, C. L., Hieger, I., Kennaway, E. L., and Mayneord, W. V.—(1937) *Amer. J. Cancer*, **42**, 295.
Crocker, H. R., and Pernet.—(1901) *Brit. med. J.*, ii, 864.
Currie, A. N.—(1947) *Brit. med. Bull.*, **4**, 402.
Daels, F.—(1923) ' Congr. du Cancer, Strasbourg.'
Darier, J.—(1902) *Ann. Derm. Syph.*, 4th ser., t. iii, 1121, *cit.* Pye-Smith, 1913*b*.—(1914/15) *Ibid.*, **5**, 449.—(1920) *Ibid.*, **1**, 49, *cit.* Anderson, 1932.
Deelman, H. T.—(1928) *Ann. Méd.*, **24**, 360.
Dennie, C. C., and McBride, W. C.—(1924) *J. Amer. med. Ass.*, **83**, 2082.
de Silva.—(1918) *Med. Presse*, n.s. **105**, 243.
Doerle, M., and Ziegler, K.—(1930) *Z. klin. Med.*, **112**, 237.
Doty, C. A.—(1931) *Arch. Derm. Syph., Chicago*, **23**, 585.
Dowling, G. B.—(1945) *Proc. Roy. Soc. Med.*, **38**, No. 3, Sect. Derm., p. 127.
Dresel, K.—(1926) *Biochem. Z.*, **178**, 70.—(1928) *Ibid.*, **192**, 351.
Dubreuilh, W.—(1910) *Ann. Derm. Syph.*, 5th ser., t. 1.
Dustin, A. P.—(1930) *C.R. Soc. Biol.*, **103**, 953.
*Idem* and Gregoire, C.—(1933) *Ibid.*, **114**, 195.
*Idem* and Piton.—(1925) *Ibid.*, **93**, 1535.—(1929) *Bull. Acad. Méd. Belg.*, 5e sér., **9**, 26.
Ebert, M. H.—(1929) *Arch. Derm. Syph., Wien*, **158**, 365.
*Idem* and Otsuka.—(1943) *Arch. Derm. Syph., Chicago*, **48**, 413.
Editorial—(1942) *J. Amer. med. Ass.*, **120**, 124.
Eggers, H. E.—(1931) *Arch. Path.*, **12**, 983—(1932) *Ibid.*, **13**, 134.
Erdmann, H.—(1904) *Z. anorg. Chem.*, **32**, 453.
Eve, F. R.—*Cit.* Pye-Smith, 1913*b*.
Fassrainer, S.—(1936) *Zbl. Chir.*, **631**, 23.
Felloni, G.—(1930) *Pathologica*, **22**, 256.
Fernandez, A. A.—(1925) *Semana méd.*, **32**, ii, 485.
Finner, L. L., and Calvery, H. C.—(1939) *Arch. Path.*, **27**, 433.

FISCHER, A.—(1926) *C.R. Soc. Biol.*, **94**, 1217.
FISCHER, W.—(1932) *cit. Dermatol.* (1939), **80**, 86.
FISCHER-WASELS, B.—(1928) *Münch. med. Wschr.*, p. 73.—(1929) *Virch. Arch. path. Anat. Physiol.*, **275**, 723.
FORDYCE, J. A., AND MCKEE.—(1914) *J. cutan. Dis.*, **32**, 446.
FRANSEEN, C. C., AND TAYLOR.—(1934) *Amer. J. Cancer*, **22**, 287.
FRASER, J. F.—(1927) *Arch. Derm. Syph., Chicago*, **16**, 518.—(1928) *Ibid.*, **18**, 809.—(1929*a*) Discussion to Wise, F., *ibid.*, **19**, 1.—(1929*b*) *Ibid.*, **19**, 306.
FROHN, W.—(1938) *Münch. med. Wschr.*, **85**, ii, 1630.
FUHS.—(1930) *Derm. Wschr.*, **90**, 457, *cit. Cancer Rev.*, 1932, **7**, 471.—(1932) *Ibid.*, **94**, 693.
FUNK, C.—(1915) *J. exp. Med.*, **21**, 571, 574.
GARCIA, P. P., AND RUIZ, F. R.—(1928) *Semana Méd.*, **35**, 515, *cit. Cancer Rev.*, 1930, **5**, 7.
GAUVIN.—(1927) *Arch. Derm. Syph., Chicago*, **15**, 350, *cit.* Franseen and Taylor, 1934.
GEYER, L.—(1898) *Arch. Derm. Syph. Wien*, **43**, 221.—(1940) *Klin. Wschr.*, **19**, 750.
GIES, TH.—(1877) *Arch. exp. Path. Pharm.*, **8**, 175.
GLEN, E. M.—(1945) *Proc. Roy. Soc. Med.*, **38**, No. 3, Sect. Derm., p. 127.
GOECKERMAN, W. H., AND WILHELM.—(1940) *Arch. Derm. Syph., Chicago*, **42**, 641.
GRAY, A. M. E.—(1912) *Brit. J. Derm.*, **24**, 325.
GROSS, P.—(1931) *Arch. Derm. Syph., Chicago*, **23**, 1132.
GUGGENHEIM, L.—(1933) *Arch. Derm. Syph., Wien*, **168**, 26.
GUTHMANN, H.—(1938) *Archiv. Gynaek.*, **166**, 526.
GUTZEIT.—(1879) *Pharm. Z.*, **24**, 263.
HAAGEN, E.—(1928) *Dtsch. med. Wschr.*, **54**, i, 92.
HAAGENSEN, C. D.—(1931) *Amer. J. Cancer*, **15**, i, 656–659.
HAERTING, F. H., AND HESSE, W.—(1879) *Vjschr. Med. gerichK.*, **30**, 296 ; **31**, 102, 313.
HALL, A. F.—(1944) *Arch. Derm. Syph., Chicago*, **50**, 148.
HAMILTON, A.—(1925) ' Industrial Poisons in the United States,' New York, p. 206.
HAMILTON, G. R.—(1921) *Brit. J. Derm.*, **33**, 15, 31.
HARBITZ, H. F.—(1927) *Lancet*, **212**, 70.
HARTZELL, M. B.—(1899) *The New Sydenham Soc.*, **170**, 259.
HARTZELL AND STELWAGON.—(1899) *Trans. Amer. derm. Ass.*, p. 11.—*Amer. J. med. Sci.*, **118**, 265.
HAXTHAUSEN, H.—(1923) *Hospitalstidende*, 44.
HEFFTER, A., AND KEESER, E.—(1927) ' Handb. exp. Pharm.', vol. iii, 1 Haelfte, p. 463.—(1937) *Erg-Bd.*, 1937, **3**, 162.
HERMANN, L.—(1879) ' Lehrbuch. d. exp. Toxikologie,' Berlin.
HEUBNER, W.—(1907) *Arch. exp. Path. Pharmak.*, **56**, 370.
HERXHEIMER—(1939) *cit. Dermatol.*, (1939), **80**, 86.
HOFMANN, E.—(1928) *cit. Dermatol.* (1939), **80**, 86.
HOPKINS, R. AND VAN STUDDIFORD, M. T., *cit. Dermatol.* (1939), **80**, 86.
HUECK, W.—(1939/40) *Z. Krebsforsch.*, **49**, 312.
HUEPER, W. C.—(1942*a*) *Cancer Res.*, **2**, 551.—(1942*b*) ' Occupational Tumours,' Baltimore (Thomas).—(1943) *Lancet*, i, 538.
*Idem* AND ITAMI, S.—(1933) *Amer. J. Cancer*, **17**, 106.
HUTCHINSON, J.—(1887) *Brit. med. J.*, ii, 1280.—(1888) *Trans. path. Soc. London*, **39**, 352.—(1894) *Arch. Surg. Lond.*, 9, **63**, 223.—(1895) ' A Smaller Atlas of Illustr. of Clin. Surg.,' London.—(1898) *Arch. Surg. Lond.*, **9**, 63, 223.—(1902) *Policlinic*, **6**, 336.—(1903) ' Atlas Clin. Med. Surg. and Path.', VI, p. 71.
HYDE, J. N.—(1899) *Trans. Amer. derm. Ass.*, p. 18.
JUSTUS, J.—(1905) *Derm. Z.*, **12**, 277.
KATHE, J.—(1937) ' Oberschl. Ges. vaterl. Kult. Breslau, Mednaturw. R.,' No. 3.
KAUFFMANN, FR.—(1929) *Z. Krebsforsch.*, **30**, 136; ref. in *Cancer Rev.*, **5**, 180.

KENNAWAY, E. L.—(1925) *J. industr. Hyg.*, **7**, 69.—(1942) *Lancet*, ii, 769.—(1943) *Ibid.*, i, 599.
KETRON.—(1927) *Arch. Derm. Syph., Chicago*, **16**, 642, 643.
KOERBLER, J.—(1939) *Liječn Vijesn.*, **61**, 590.
KONRAD.—(1929) *Derm. Wschr.*, **88**, 690.
KROTKINA, N.—(1934) *Vop. Onkol.*, **6**, 119; ref. in *Z. Krebsforsch.*, 1935, **41**, 251.
LABES, R.—(1929) *Arch. exp. Path. Pharmak.*, **141**, 148.
LACLAU, N. C., AND PILLADO-MATHEU, C.—(1930) *C.R. Soc. Biol.*, **103**, 1285.
LANE, C.—(1939) *Arch. Derm. Syph., Chicago*, **40**, 105.
LANE, W. A.—(1894*a*) *Trans. Clin. Soc.*, **27**, 102.—(1894*b*) *Brit. med. J.*, i, 354.
LANGE, K.—(1935) *Z. Krebsforsch.*, **42**, 306.
LA ROSSA.—(1925) *Rass. int. Clin. Terap.*, **6**, 28.
LAYMON, C. W.—(1943) *Arch. Derm. Syph., Chicago*, **48**, 681.
LEGGE, T. M.—(1902) 'Ann. Rep. Chief Insp. Fact. and Worksh.'—(1923) *Ibid.*, (1924) *Ibid.*—(1925) *Ibid.*—(1934) 'Industrial Maladies,' Oxford, p. 84.
LEITCH, A.—(1923) *Brit. med. J.*, ii, 1.
*Idem*, AND KENNAWAY, E. L.—(1922) *Ibid.*, ii, 1107.
LEVIN, O. L.—(1926) *Arch. Derm. Syph., Chicago*, **13**, 569.
LIPSCHUETZ.—(1924) *Arch. Derm. Syph., Wien*, **147**, 520.
LITTLE, GRAHAM.—(1923) *Brit. J. Derm.*, **35**, 435.
LOEWENBERG, M.—(1912) *Verh. Ges. dtsch. Naturf. Ärzt.*, **84**, p. 72, Hlfte, p. 312 see also Ullmann, 1922.
LOEWY, J.—(1929) *Med. Klinik*, **25**, 141.
LORENZ, E.—(1944) *J. Nat. Cancer Inst.*, **5**, 1.
LUDEWIG, P., AND LORENSER, E.—(1924*a*) *Z. Phys.*, **22**, 178.—(1924*b*) *Strahlentherapie*, **17**, 428.
MACCORMAC.—(1933*a*) *Proc. Roy. Soc. Med.*, **26**, 1553.—(1933*b*) *Brit. J. Derm.*, **45**, 475.
MCCOY, J. N.—(1920) *Arch. Derm. Syph., Chicago*, **6**, 175.
MCJUNKIN, F. A., AND CIRKIT, M. F.—(1929) *Proc. Soc. exp. Biol.*, **27**, 179.
MACLEOD, J. M. H.—(1913) *Proc. Roy. Soc. Med.*, vi, 1, Derm. Sect., p. 109.
MCNEER, G.—(1934) *Ann. Surg.*, **99**, 348.
MAISIN, J.—(1934) *Brux. méd.*, **14**, 544, *cit.* Hueper, 1942.
*Idem* AND DUPUIS.—(1929) *Rev. belge Sci. méd.*, i, 409.
MANOUVRIER.—(1876) *cit.* Bayet and Slosse (1919*a*).
MATRAS, A.—(1940) *Med. Klin.*, **36**, 1274, *cit. Bull. Hyg., Lond.*, **17**, 383.
MAYER.—(1933) *Handb. d. Haut- und Geschl-krankh.*, **4**, pt. 2, 72, *cit.* Goeckerman and Wilhelm, 1940.
MEREWETHER, E. R.—(1943) 'Ann. Rep. Chief Insp. Fact. and Worksh.,' p. 45.
MIGAYI, S.—(1935) *Zbl. Chir.*, **62**, ii, 2063.
MILCH, H.—(1932) *Amer. J. Cancer*, **16**, 89.
MILIAN, G.—(1929) *Rev. franç. Derm. Vénéréol.*, p. 507.—(1931) *Ibid.*, **7**, 580.
MOELLER, E.—(1923) *Z. Krebsforsch.*, **19**, 393.
MONTEMARTINI, G.—(1928) *Tumori*, **14**, 1; ref. in *Cancer Rev.*, 1929, **4**, 269.
MONTGOMERY, H.—(1929) *Arch. Derm. Syph., Chicago*, **20**, 339.—(1935) *Ibid.*, **32**, 229.
*Idem* AND WAISMAN.—(1941) *J. Invest. Derm.*, **4**, 365.
MONTGOMERY, R. M.—(1942) *Arch. Derm. Syph., Chicago*, **45**, 407.
MYERS, C. N., AND CORNWALL, L. H.—(1925) *Amer. J. Syph.*, **9**, 647.
NANDER.—(1923) *Hospitalstidende*, **66**, 7.
NIEBERLE, K. (1939)—*Z. Krebsforsch.*, Orig., **49**, 137.
NUTT, W. H., BEATTIE, J. M., AND PYE-SMITH, R. J., *see* Pye-Smith (1913).
O'DONOVAN, W. J.—(1924) *Brit. J. Derm.*, **36**, 477.—(1928) *J. State Med.*, **36**, 700.
O'LEARY, P. A.—(1935) *Arch. Derm. Syph., Chicago*, **31**, 413.

OLIVER.—(1923) *Ibid.*, **8**, 267.
*Idem* AND FINNERUD.—(1928) *Ibid.*, **17**, 892, *cit. Cancer Rev.*, 1929, **4**, 21.
ONAKA, O.—(1911) *Z. physiol. Chem.*, **70**, 433.
OPPENHEIM, M.—(1931) *Dermatol. Wschr.*, **92**, 741.
OPPENHEIM AND FANTL.—(1934) *Arch. Derm. Syph., Wien*, **170**, 488, *cit.* Montgomery and Waisman, 1941, discussion.
ORMSBY AND MITCHELL.—(1925) *Arch. Derm. Syph., Chicago*, **12**, 144, 426.
OSBORNE.—(1925) *Ibid.*, **12**, 773.—(1928) *Ibid.*, **18**, 37.—(1932) *Ibid.*, **25**, 419.
OSTERBERG, A. E.—(1928) *J. biol. Chem.*, **76**, 19.
*Idem* AND GREEN, W.—(1944) *Ibid.*, **155**, 513.
OWEN.—(1921) *J. Amer. med. Ass.*, **76**, 1329.
PACK, G. T., AND LEFEVRE, R. G.—(1930) *J. Cancer Res.*, **14**, 167.
PARIS, J. AYRTON.—'Pharmacologia,' 3rd ed., 1820, London, *cit.* Kennaway, 1942.
PECK, S. M.—(1942) *Arch. Derm. Syph., Chicago*, **45**, 1016.
v. PEIN, H.—(1938*a*) *Dtsch. med. Wschr.*, **64**, 565.—(1938*b*) *Münch. med. Wschr.*, 1017.
*Idem* AND BAURHENN.—(1943) *Klin. Wschr.*, **22**, 388.
PELLER, S.—(1939) *Hum. Biol.*, **11**, 130.
PENTIMILLI, F.—(1926) *Lo Esperimentale*, **80**, 624 ; ref. in *Cancer Rev.*, 1928, **5**, 163.
PETROFF, N., AND KROTKINA, N.—(1928) *Bull. Ass. franç. Cancer*, **41**, 251.
PFAHLER.—(1927) *Arch. Derm. Syph., Chicago*, **15**, 213.
PIRCHAN AND ŠIKL, H.—(1932) *Amer. J. Cancer*, **16**, 681.
PITON, R.—(1929) *Arch. int. Méd. exp.*, **5**, 355.
PORTER, CH. R.—*Cit.* Pye-Smith, 1913*b*.
POSNER.—(1904) *Z. Krebsforsch.*, **1**, 4.—(1905) *Dtsch. Klin.*, **10**, 455.—(1924) *Z. Urol.*, **18**, 418.
POZZI.—(1874) *Bull. Soc. anat. Paris*, *cit.* Hartzell, 1899.
PRELL, H.—(1936/37) *Arch. Gewerbepath. Gewerbehyg.*, **7**, 656.
PUCCINELLI, E.—(1930*a*) *Pathologica*, **22**, 63.—(1930*b*) *Ibid.*, **22**, 349.
PUTMAN, F. L.—(1945) *Arch. Derm. Syph., Chicago*, **51**, 225.
PYE-SMITH, R. J.—(1913*a*) *Proc. Roy. Soc. Med., Clin. Sect.*, **6**, I, 229.—(1913*b*) *Lancet*, ii, 210, 282.
RAMEL.—(1929) *Schweiz. med. Wschr.*, **10**, 1230, *cit. Cancer Rev.*, **5**, 404.
RAPOSO, S.—(1928) *C.R. Soc. Biol.*, **98**, 86.—(1929) *A. ch. Port. Sci. Biol.*, **2**, 146, 186.
RASCH.—(1930) *Internat. Congr. Derm. Syph., Copenhague*, cit. *Dermatologica*, 1930, **80**, 86.
RAUSCHKOLB (ARDAY, E. J.).—(1938) *Arch. Derm. Syph., Chicago*, **37**, 331.
RAVAUT, P.—(1920) *Pr. méd.*, **28**, 73, *cit.* Franseen and Taylor, 1934.
REMENOVSKI, F.—(1921) *Arch. Derm. Syph., Wien*, **131**, 465.
RINGER, S., AND MURREL, W.—(1878/79) *J. Physiol.*, **1**, 213.
ROBINSON, S.—(1935) *Arch. Derm. Syph., Chicago*, **31**, 260.—(1937) *Ibid.*, **36**, 870, discussion to Traub.—(1941) *Ibid.*, **44**, 78, discussion to Rodgers.
ROCMANS, M.—(1930) *C.R. Soc. Biol.*, **103**, 42.
RODGERS, J. D.—(1941) *Arch. Derm. Syph., Chicago*, **44**, 78.
ROFFO, A. H., AND CORREA, L. M.—(1926) *Bol. Inst. Med. Esp.*, **3**, 367.
*Idem* AND ROSNER, S.—(1935) *Rev. Ass. méd. Argent.*, **49**, 1931.—(1936) *Semana méd.*, **1**, 861.
ROMBERG.—'Klin. Wahrnehmungen u. Beob.,' 1851.
ROSEN.—(1935) *Arch. Derm. Syph., Chicago*, **32**, 218 ; discussion to Montgomery.
ROSENTHAL, S. M., AND VOEGTLIN.—(1930) *J. Pharmacol.*, **39**, 246.
ROST, E.—(1931) 'Handb. d. Lebensm-chemie,' Berlin, **1**, 1073, *cit.* Wuehrer, 1937.
ROSTOSKI, O., SAUPE AND SCHMORL.—(1926) *Z. Krebsforsch.*, **23**, 360.—(1927) *Ibid.*, **25**, 249.
*Idem* AND SAUPE, E.—(1931) *Arch. Gewerbepath. Gewerbehyg.*, **1**, 731.

RUSSELL, B. F., AND KLABER, R.—(1945) *Proc. Roy. Soc. Med.*, **38**, No. 3, Sect. Derm., p. 128.
RYAN, M. L.—(1939) *Arch. Derm. Syph., Chicago*, **40**, 104.
SALKOWSKI, E.—(1908) *Z. physiol. Chem.*, **56**, 95.
SAUPE, E.—(1930) *Arch. Gewerbepath. Gewerbehyg.*, **1**, 582.
SCHABAD, L. M.—(1935) *Z. Krebsforsch.*, **42**, 295.
SCHAERRER, W. C.—(1934) *University Western Ontario Med. J.*, **5**, 27.
SCHAMBERG, J. F.—(1907*a*) *J. cutan. Dis.*, **25**, 26.—(1907*b*) *Ibid.*, **27**, 130, *cit.* Pye-Smith, 1913*b*.
SCHILLER, W.—(1926) *Z. Krebsforsch.*, **23**, 9.
SCHINZ, H. R.—(1942) *Schweiz. med. Wschr.*, **72**, 1070.
SCHMIEDEBERG, O.—(1906) ' Grundviss der Pharmakologie,' Leipzig (Vogel) p. 476.
SCHREINER, B. T., AND WEHR, W. H.—(1934) *Amer. J. Cancer*, **20**, 418.
SCHWARTZ.—(1926) *Arch. Derm. Syph., Chicago*, **13**, 705.
SCHWARZ, L.—(1932) *Derm. Wschr.*, **94**, 577.
*Idem* AND DECKERT, W.—(1943) *Arch. Hyg. Berl.*, **129**, 276 ; ref. *Bull. Hyg. Lond.*, (1943), **18**, 927.
SEMINARIO, C., AND ALVARADO, G. E. R.—(1931) *Semana méd.*, **36**, i, 1665 ; ref. in *Cancer Rev.* (1931) 475.
SEMON, H. C.—(1922) *Brit. med. J.*, 975.—(1945) *Proc. Roy. Soc. Med.*, **38**, No. 3, Sect. Derm., p. 128, discussion.
ŠIKL, H.—(1930) *Z. Krebsforsch.*, **32**, 609.
SIMONS, R.—(1937) ' Arsenicodermie,' Leiden, 1937.
SPENCER.—Discussion to Pye-Smith (1913*a*).
STELWAGON, H. W.—(1910) ' Treatise on Diseases of the Skin,' 6th ed., London.
STEWART.—(1934) *J. Urol.*, **30**, 170 ; *cit.* Hueper, 1942.
STICKER, A.—(1911) *Zbl. Bakt.*, **59**, 464.
STILLIANS, A. W.—(1919) *J. cutan. Dis.*, **37**, 269.—(1930) *Arch. Derm. Syph., Chicago*, **22**, 1085.—(1931) *Ibid.*, **23**, 377 ; *cit.* Anderson, 1932.
STOKES.—*Cit.* Peck (1942).
STRAATEN.—(1935) *Zbl. Chir.*, No. 37.
SUTTON.—(1939) ' Diseases of the Skin,' London.
TANNENHOLZ, N., AND MUIR, K. B.—(1933) *Arch. Path.*, **15**, 789.
THRONE, B., AND MYERS, C. N.—(1935) *Arch. Derm. Syph., Chicago*, **32**, 181.
TILLAUX.—(1877) *Bull. mém. soc. Chir. Paris*, **3**, 351.
TIMBERLAKE, H. P.—(1929) *U.S. Veterans Bur. Med. Bull.*, **5**, 678 ; *cit.* Franseen and Taylor, 1934.
TOEROEK.—(1891) *Ann. Derm. Syph., Paris*, 425 ; *cit.* Dubreuilh, 1910.
TRAUB, E. F.—(1937) *Arch. Derm. Syph., Chicago*, **36**, 198.
TRELLES, R. A.—(1937) *Bol. Obras. San. Nacion*, **1**, 488.
ULLMANN, K.—(1898) *Wien. klin. Wschr.*, **228**.—(1900) ' Congr. Internat. Derm. Syph. Paris C.R.', t. ix, p. 221.—(1906) *Allg. wien. med. Ztg.*, **51**, 59.—(1913) *Verh. Ges. dtsch. Naturf. Arzt.*, 1913, ii, 2, p. 305.—(1914) *Wien. klin. Wschr.*, No. 12.—(1914–15) *Wien. med. Wschr.* (*cit.* Ullmann, 1917).—(1917) *Wien. klin. Wschr.*, **30**, 829.—(1922*a*) *Arch. Derm. Syph., Wien.*, **138**, 337.—(1922*b*) *Wien. klin. Wschr.*, **35**, 455, 479, 502.—(1922*c*) *Wien. med. Wschr.*, 72, 492.—(1930) ' 8 Congr. Internat. Derm. and S., Copenhague,' p. 693.—(1932) *Wien. klin. Wschr.*, **45**, 840.—(1933) in Jadassohn, *Handb. d Haut- u. Geschl-krankh.*, Berlin, Springer, **12**, pt. 3, 628.
VICTORISZ, K.—(1931) *Biochem. Z.*, **240**, 488 ; ref. *Cancer Rev.*, 1932, 148.
v. VOLKMANN.—(1875) ' Beitr. z. Chir.,' p. 370.
VOSS, FR.—(1939) *Strahlentherapie*, **66**, 156.
WARREN, S., AND EHRENREICH.—(1944) *Cancer Res.*, **4**, 554.
WAUGH, J. F., AND SCULL.—(1935) *Arch. Derm. Syph., Chicago*, **31**, 143.

WEISKE.—(1875) *J. Landw.*
WENDE, G. W.—(1908) *J. cutan. Dis.*, **26**, 531.
WHITE, A. W. M.—(1927) *J. Cancer Res.*, **11**, 111.
WHITE, J. C.—(1885) *Amer. J. med. Sci.*, **89**, 163.—(1899) *Trans. Amer. derm. Ass.*, 1899, p. 18.
WIGLEY.—(1945) *Proc. Roy. Soc. Med.*, **38**, No. 3, Sect. Derm., 128 (discussion).
WIGNALL, T.—(1920) *Brit. med. J.*, i, 826.
WILE, U.—(1912) *J. cutan. Dis.*, **10**, 192.
WILHELM.—(1934) *Arch. Derm. Syph., Chicago*, **48**, 29, 613 (discussion).
WILHELM, L. F. X., AND GOECKERMAN, W. H.—(1943) *Ibid.*, **48**, 324.
WILSON, E.—(1871) ' Lectures on Dermatology.'
WISE, F.—(1920) *Arch. Derm. Syph., Chicago*, **2**, 197.—(1928) *Ibid.*, **18**, 809 ; *cit.* Anderson, 1932.—(1929) *Ibid*, **19**, 306, 697.
WOBST, A.—(1925) ' Die Huettenrauchkrankht. im Freiberger Bez. Freiberg i/S.
WRIGHT, C. S., AND FRIEDMAN, R. J.—(1933) *Arch. Derm. Syph., Chicago*, **27**, 70.
WUEHRER, J.—(1937) *Biochem. Z.*, **294**, 401.
YOUNG, E. G., AND RICE, F. A. H.—(1944) *J. Lab. clin. Med.*, **29**, 439.
YOUNG, M., AND RUSSELL, W. T.—(1926) *Spec. Rep. Ser. med. Res. Coun.*, No. 99.
ZIEL, R.—(1930) *Med. Klinik*, **25**, 623.—(1935) *J. industr. Hyg., Lond.*, **17**, 28.
ZIELER.—(1938) *Zbl. Haut- u. GeschlKr.*, **62**, 5, *cit.* Voss, 1939.
ZINNY AND VIVALDO, J.—(1942) ' Arsenicismo crónico regional endémico producido per las aguas del cono,' Buenos Ayres, p. 142.


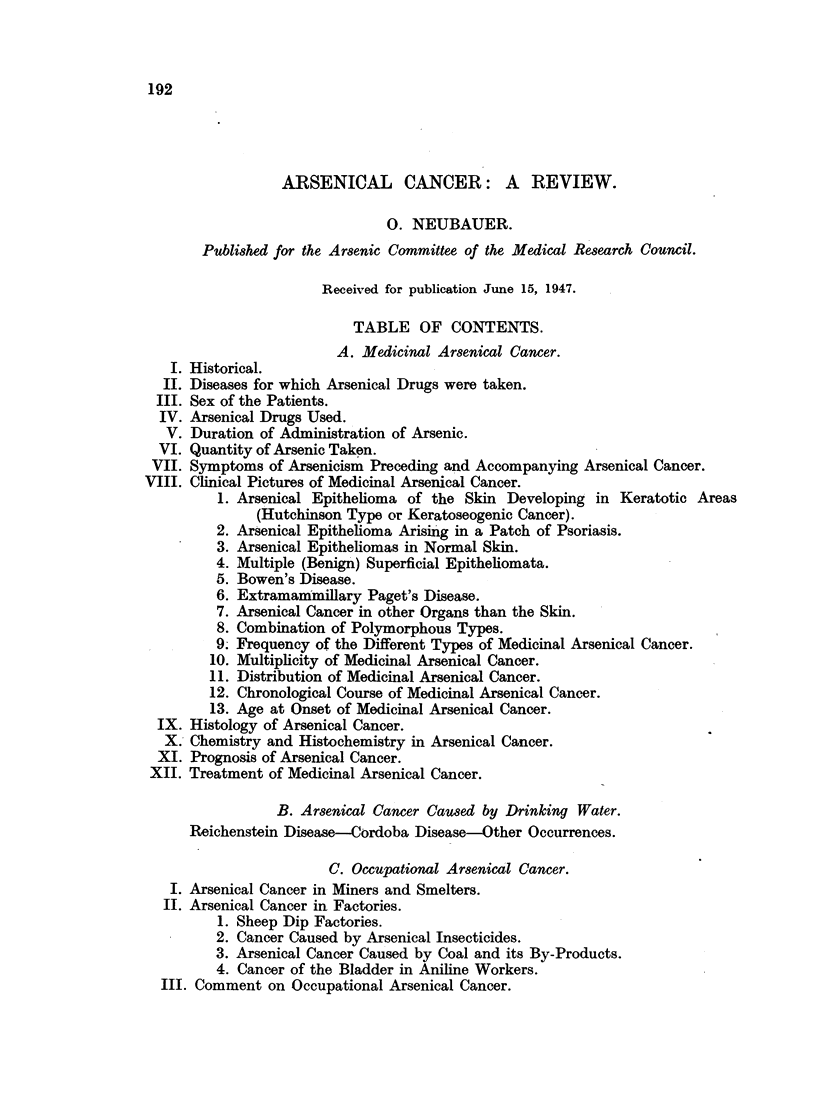

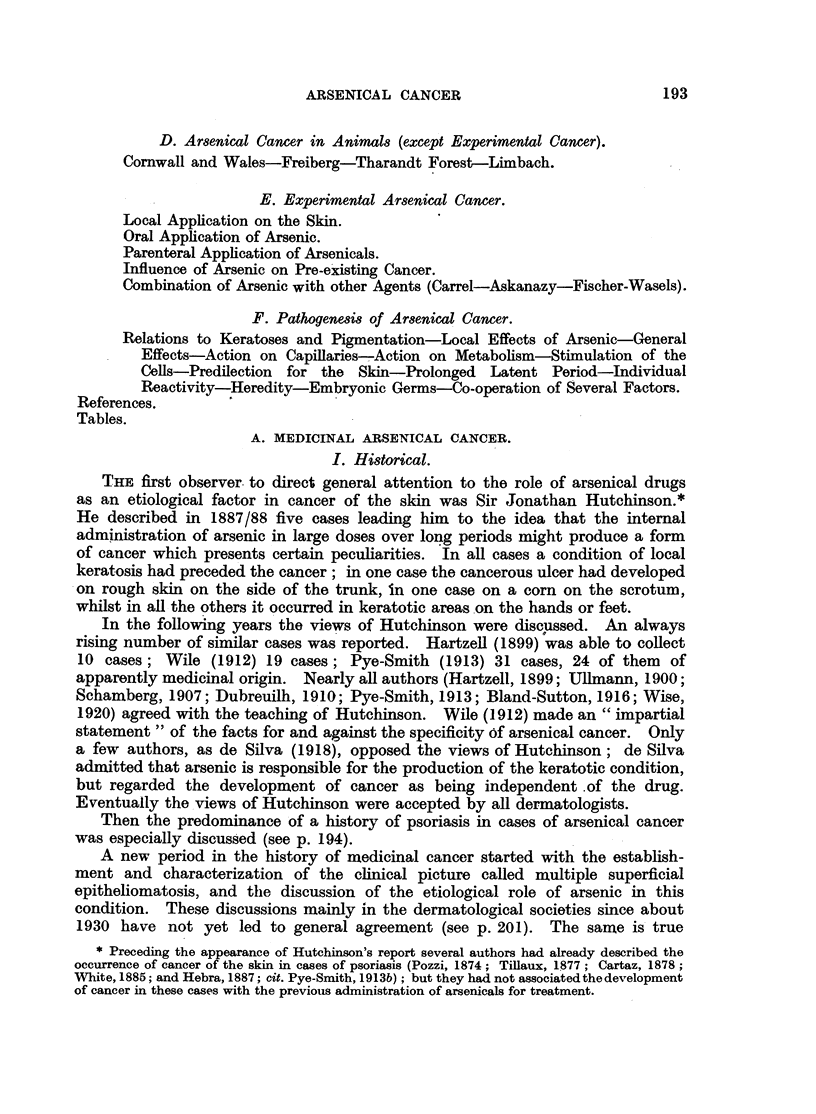

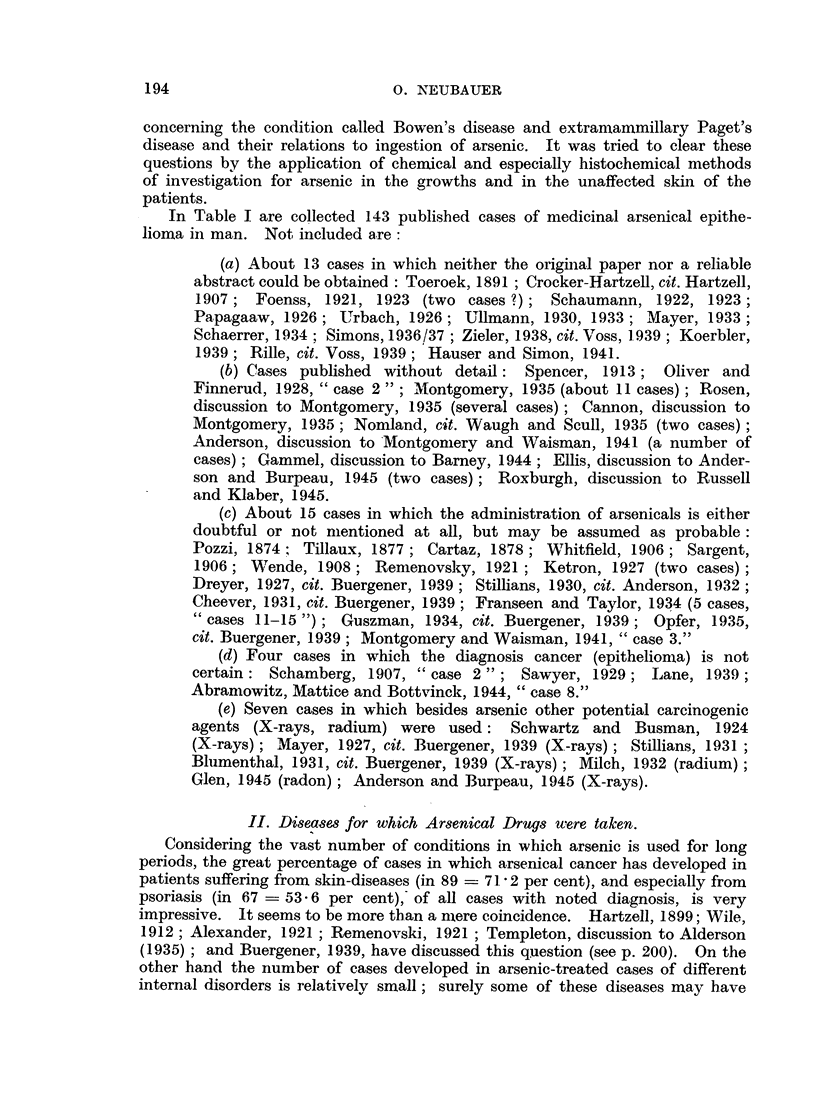

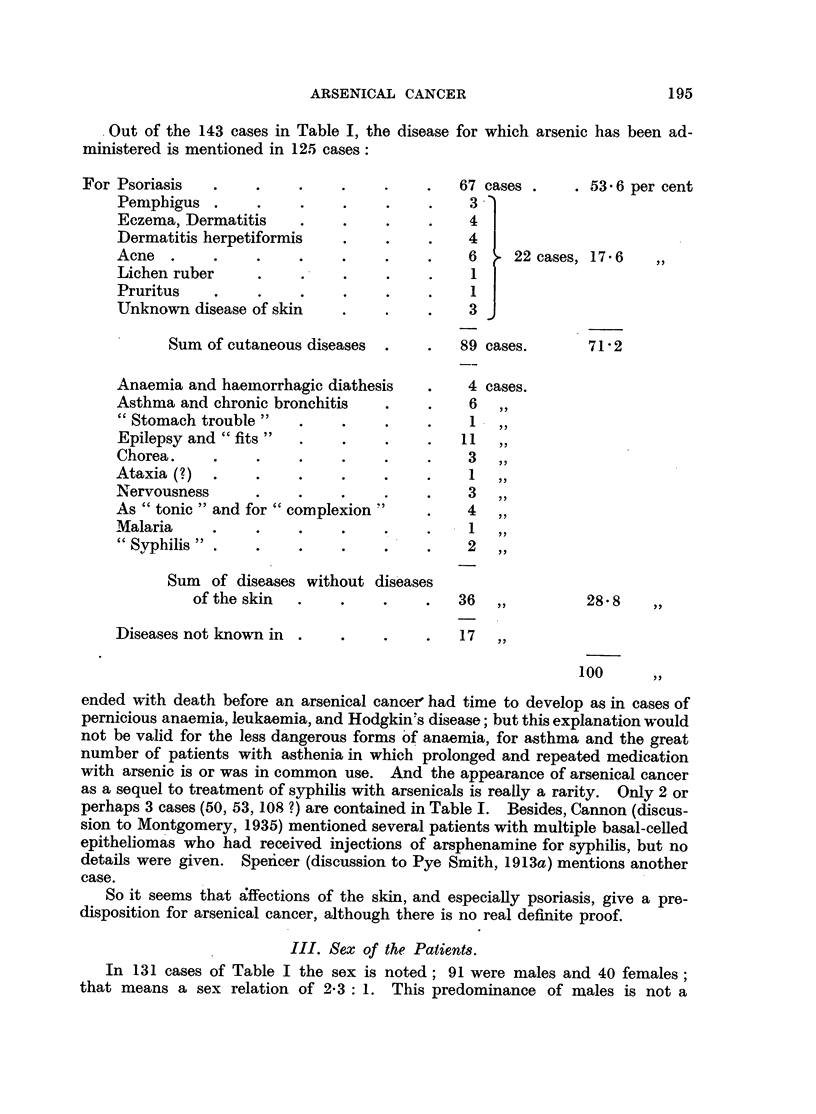

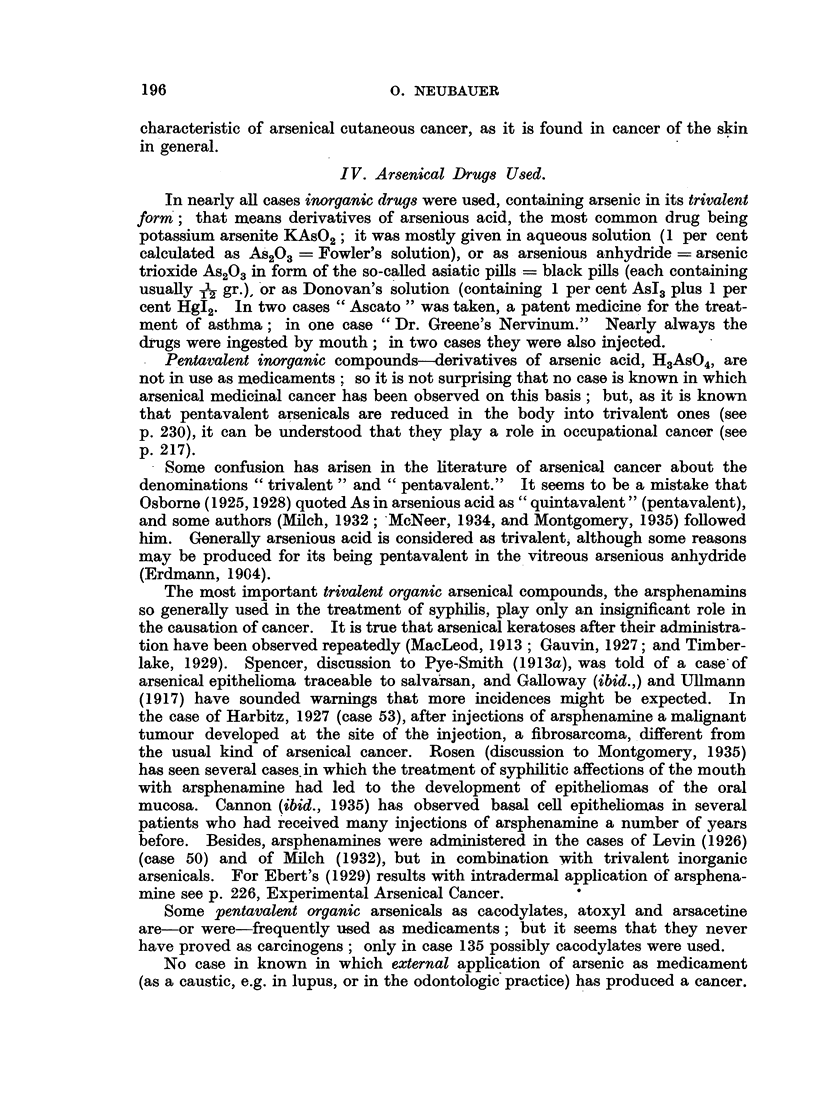

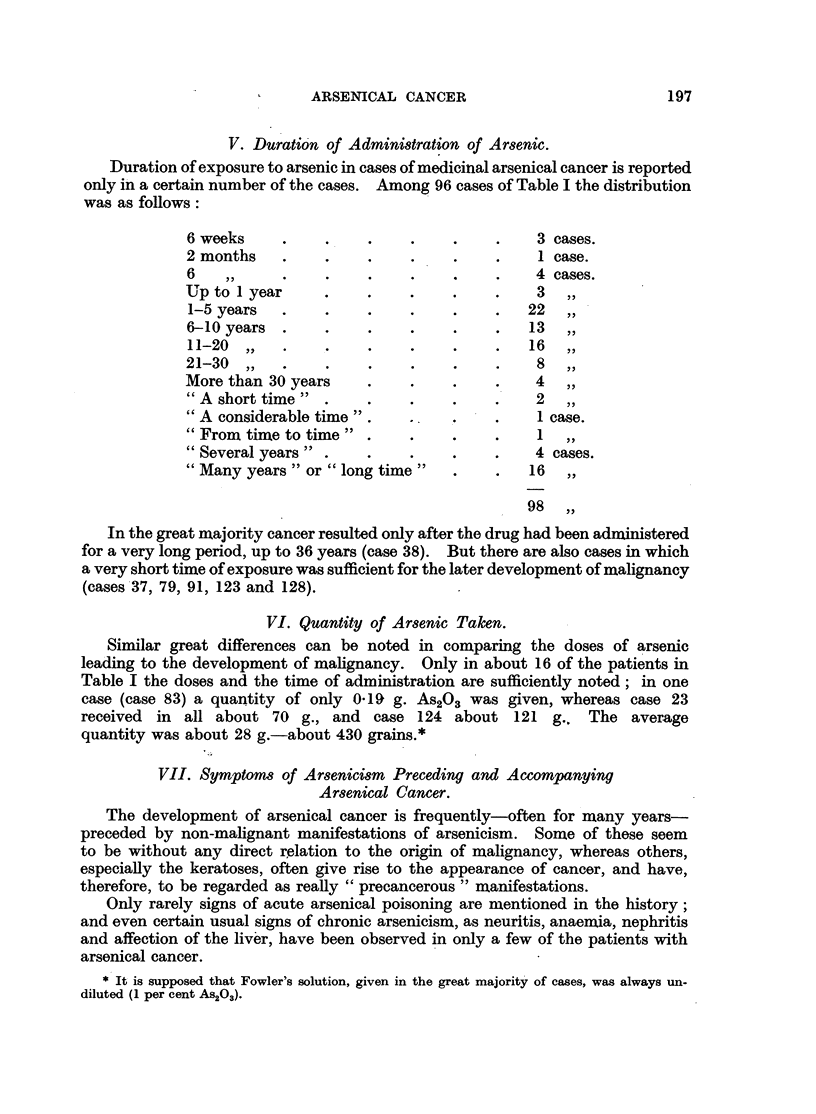

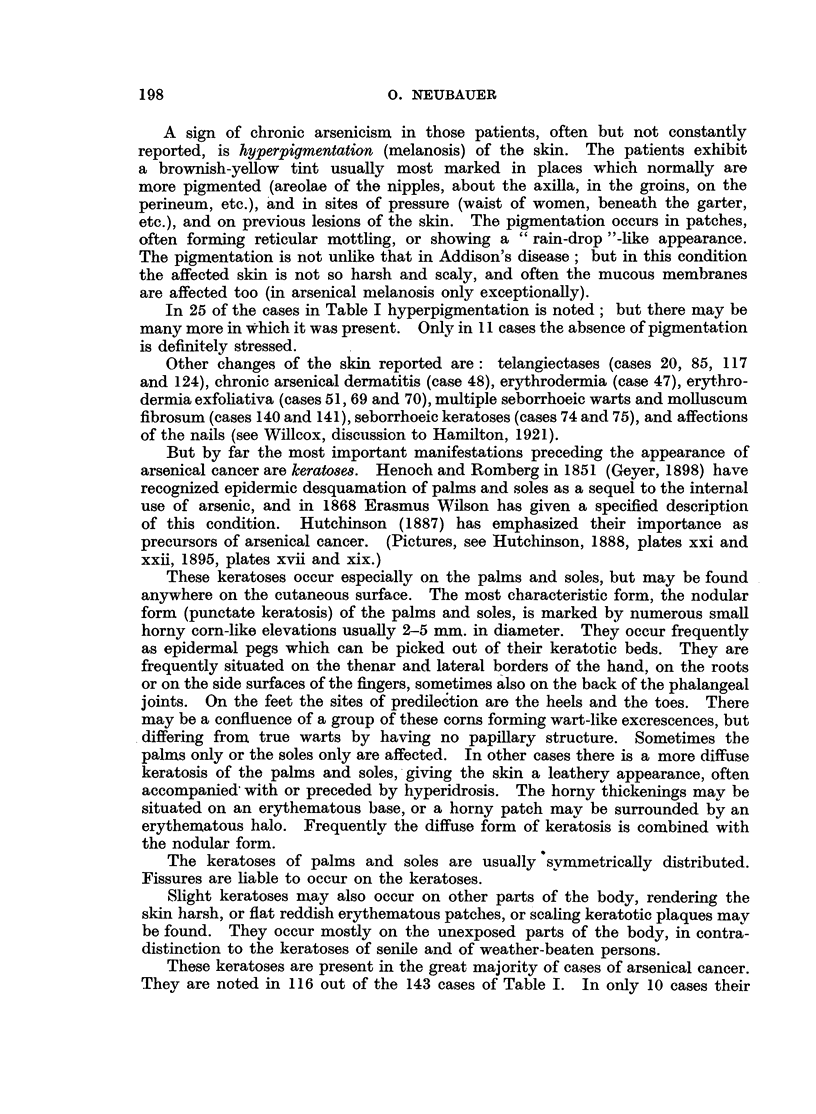

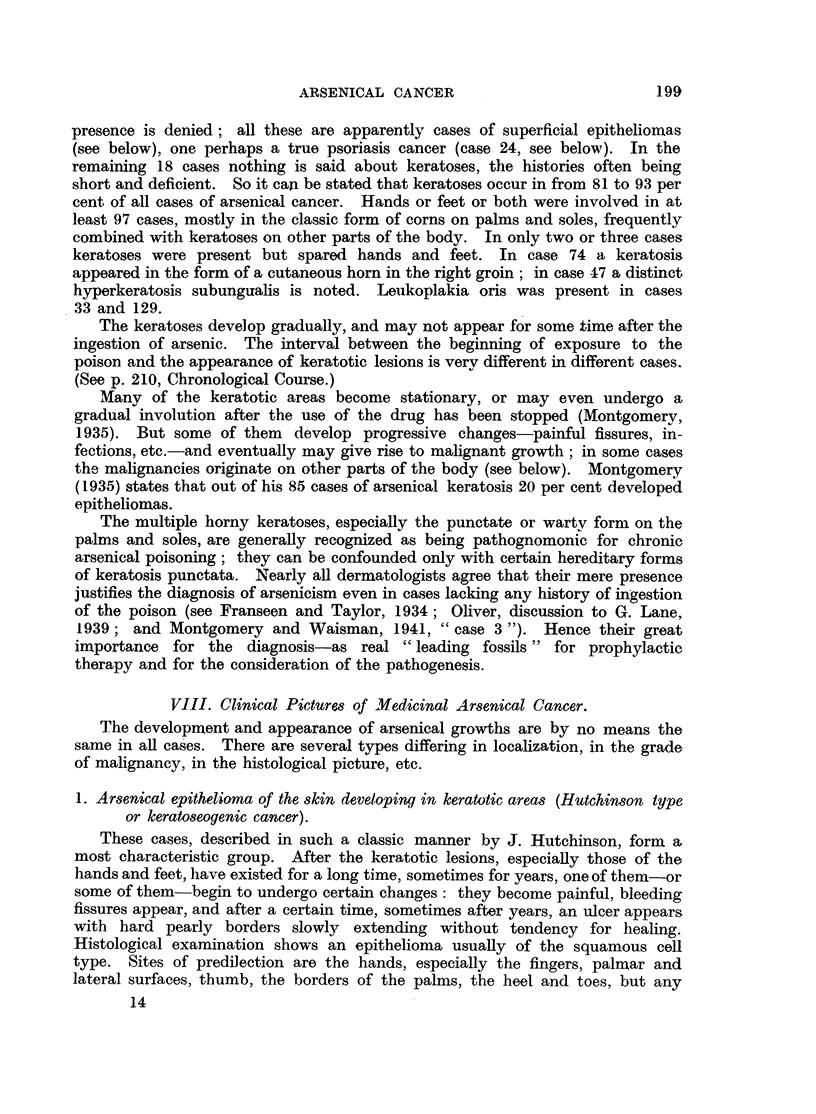

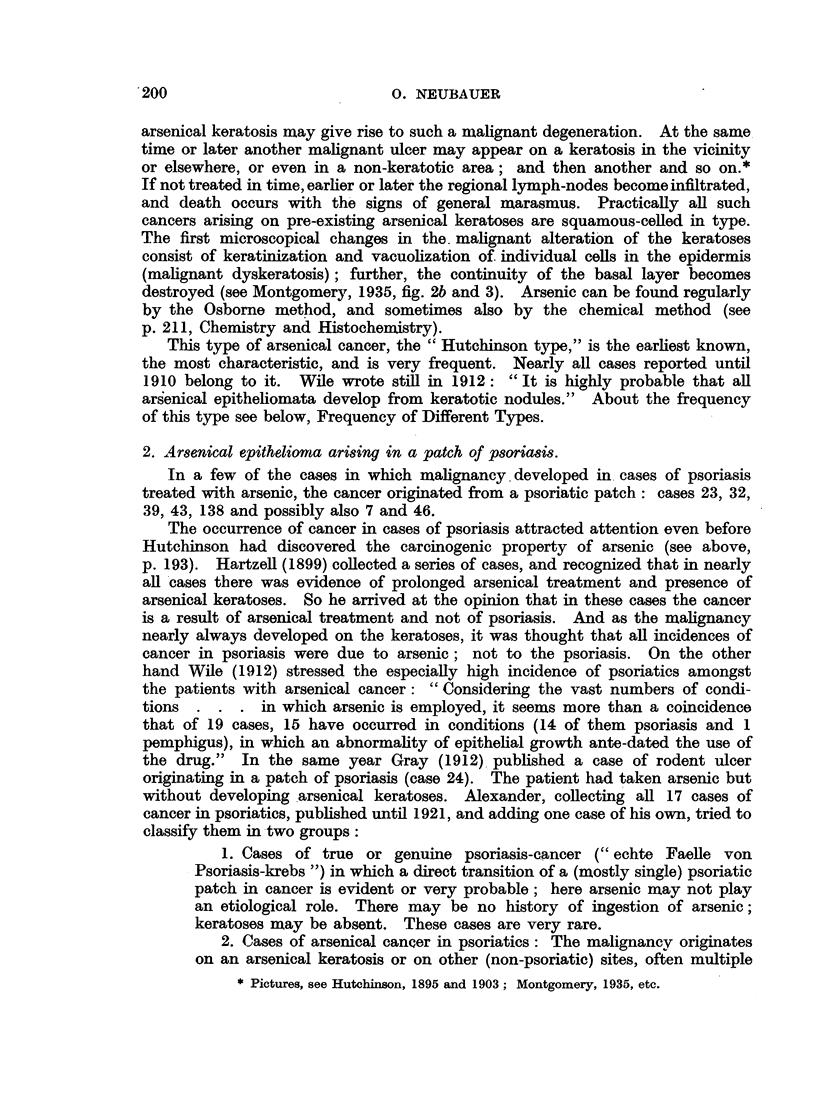

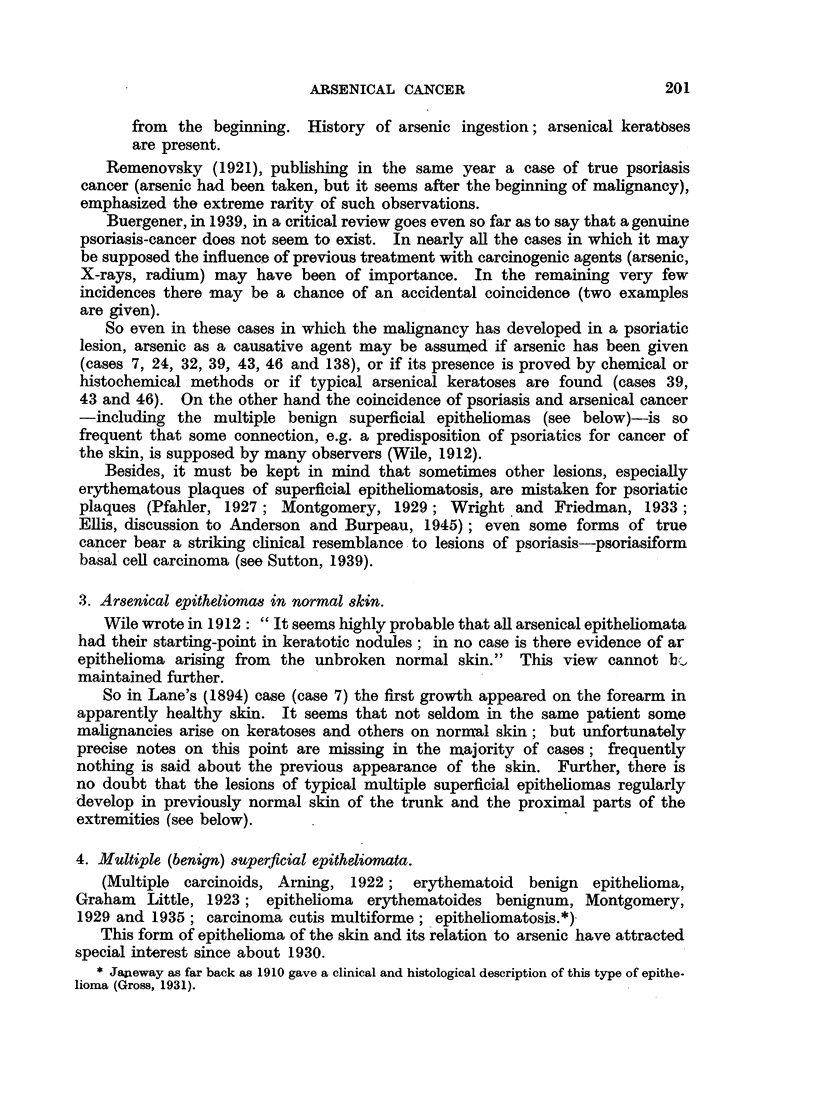

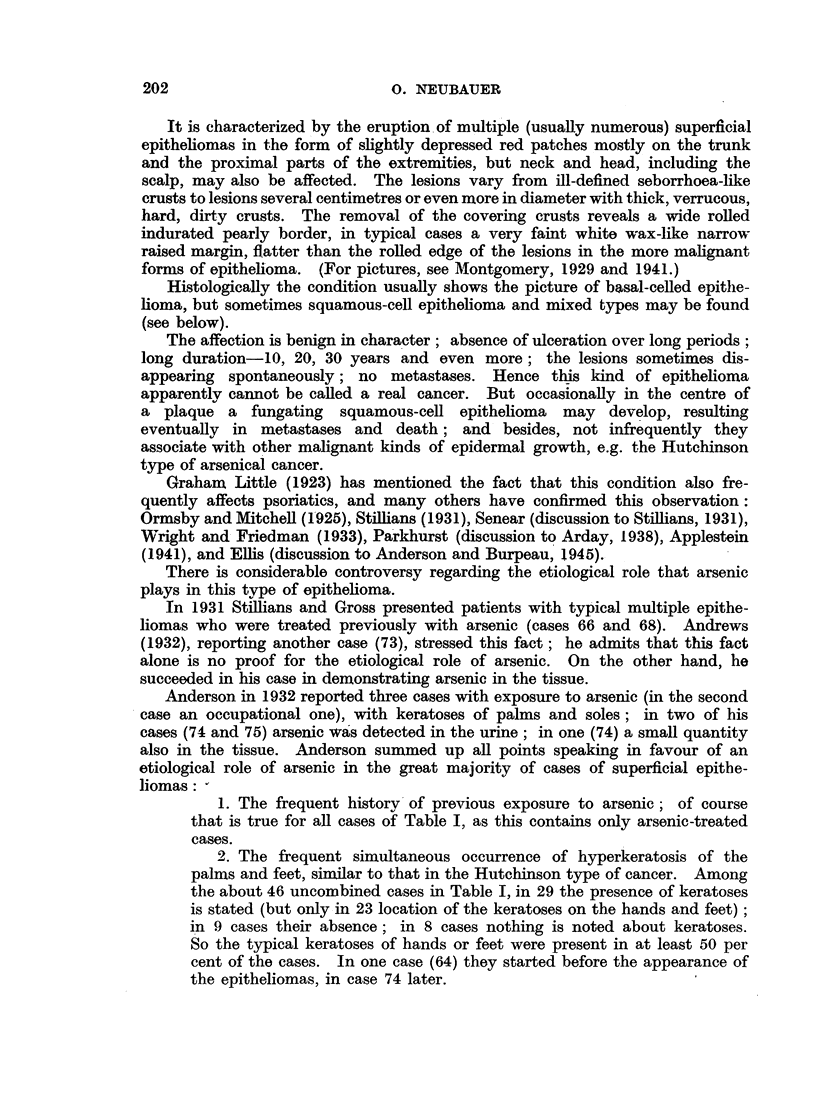

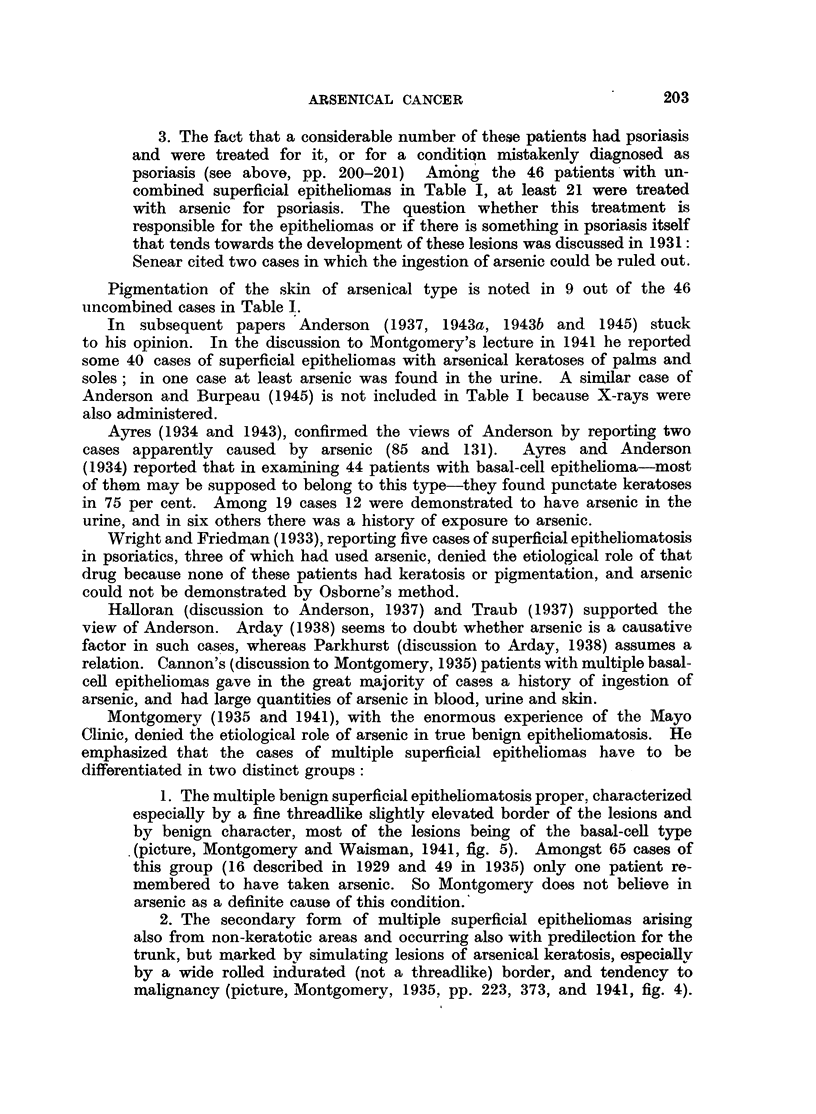

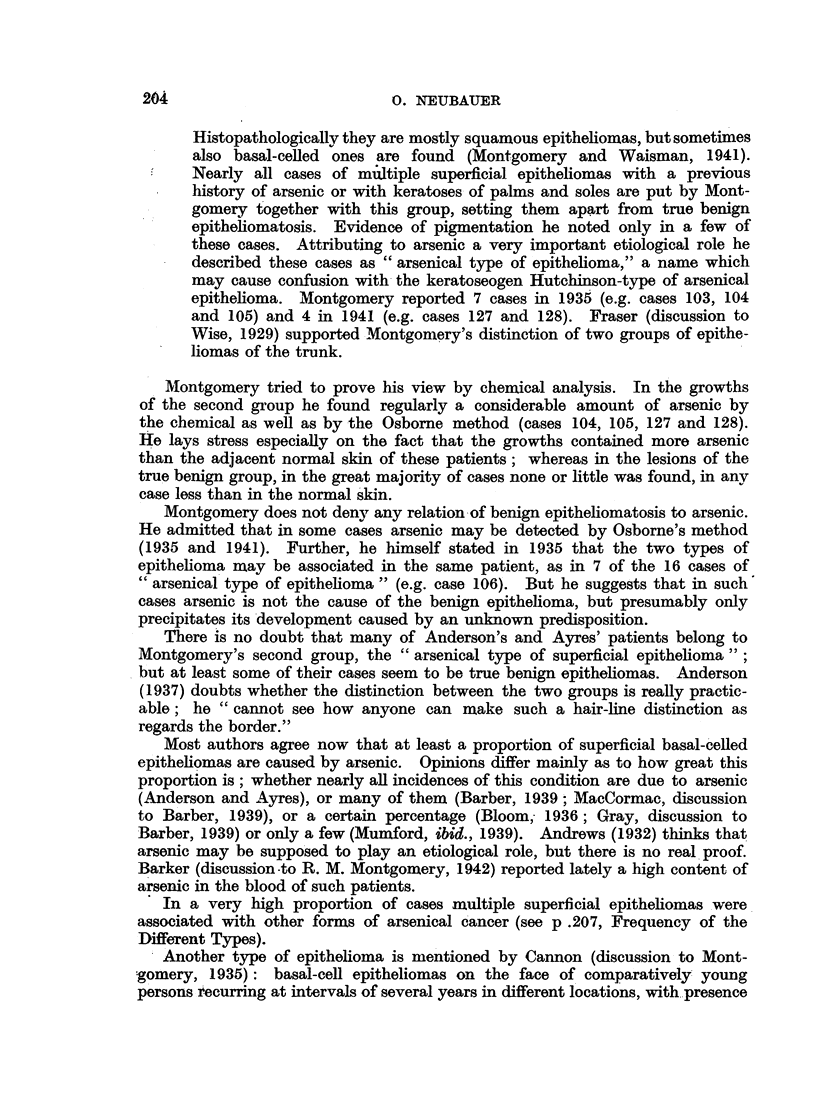

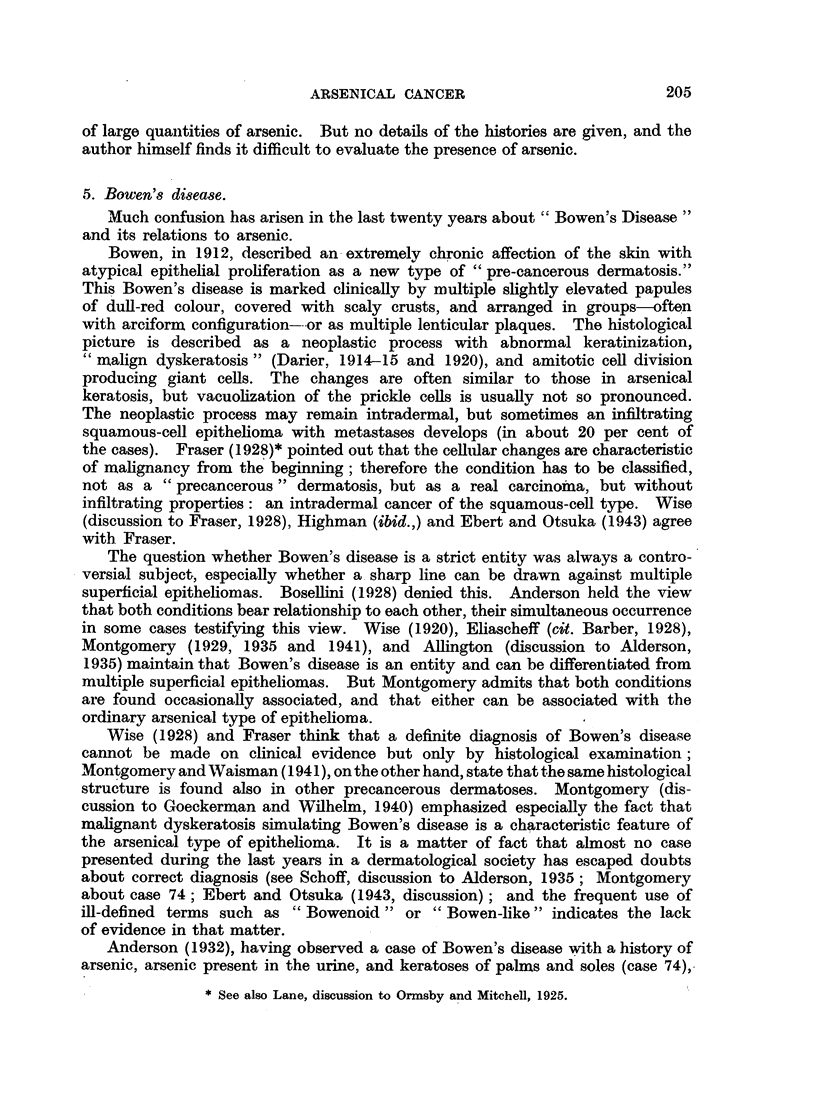

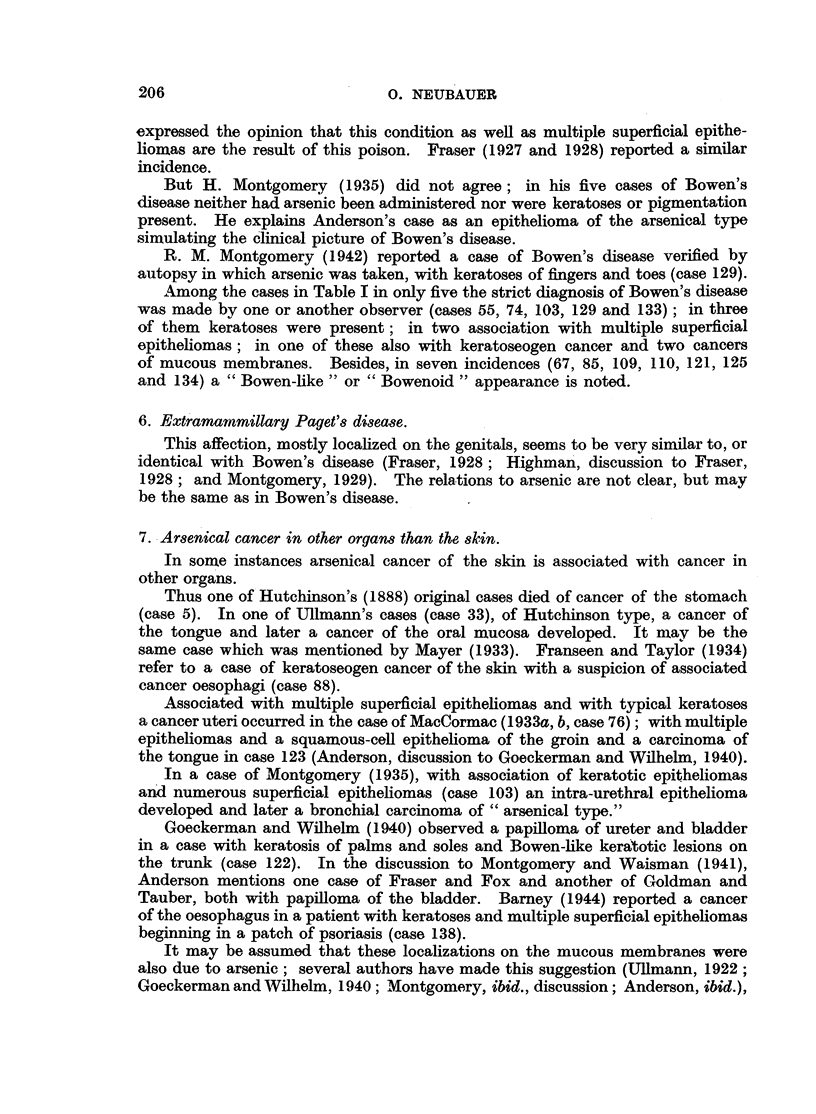

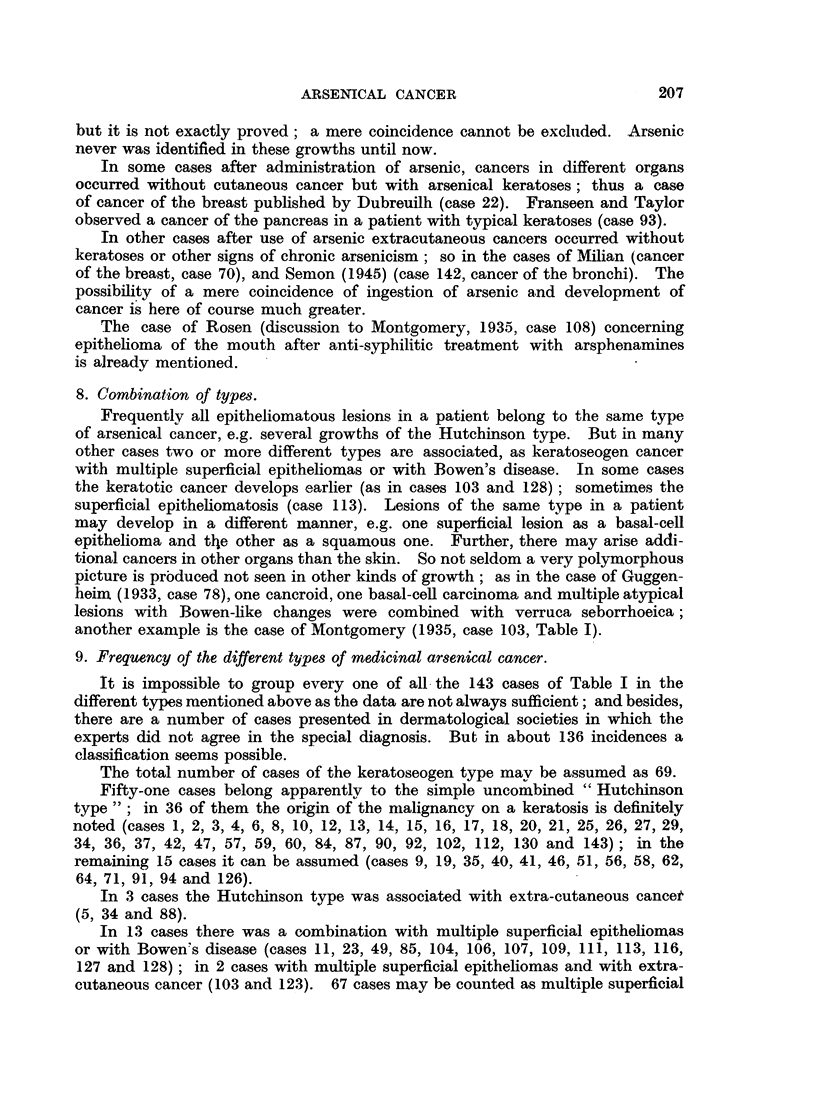

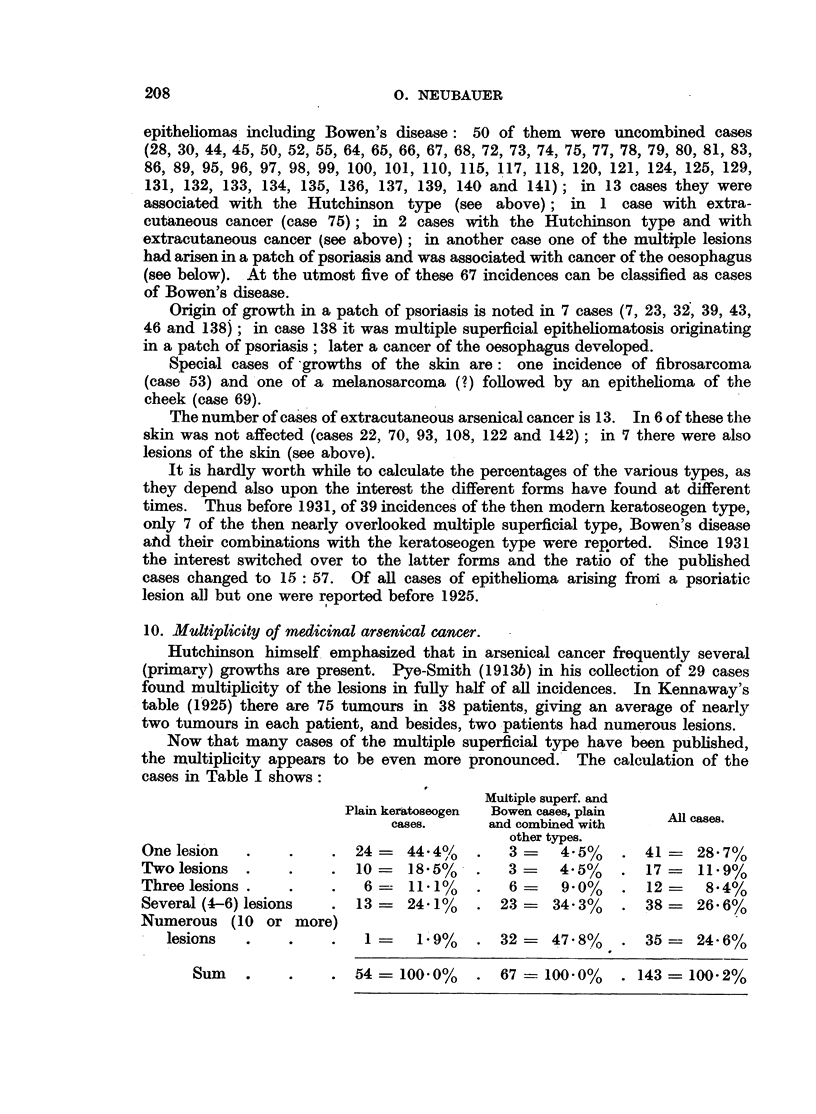

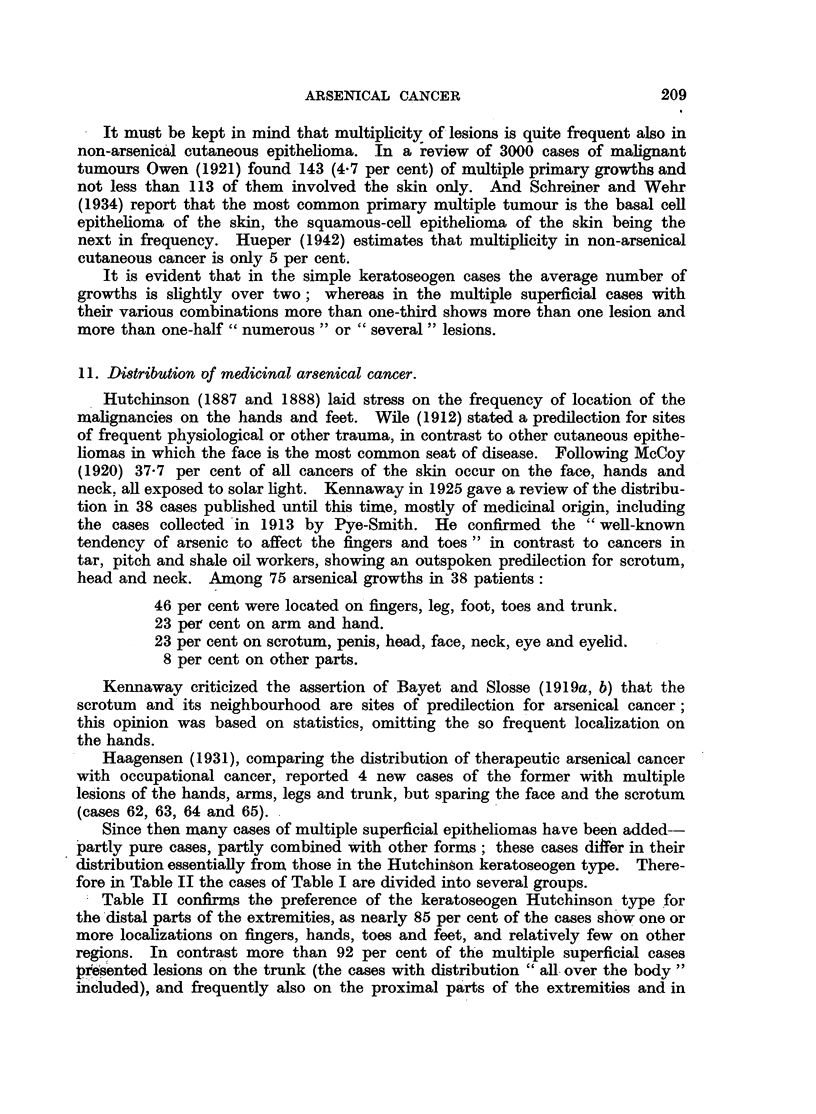

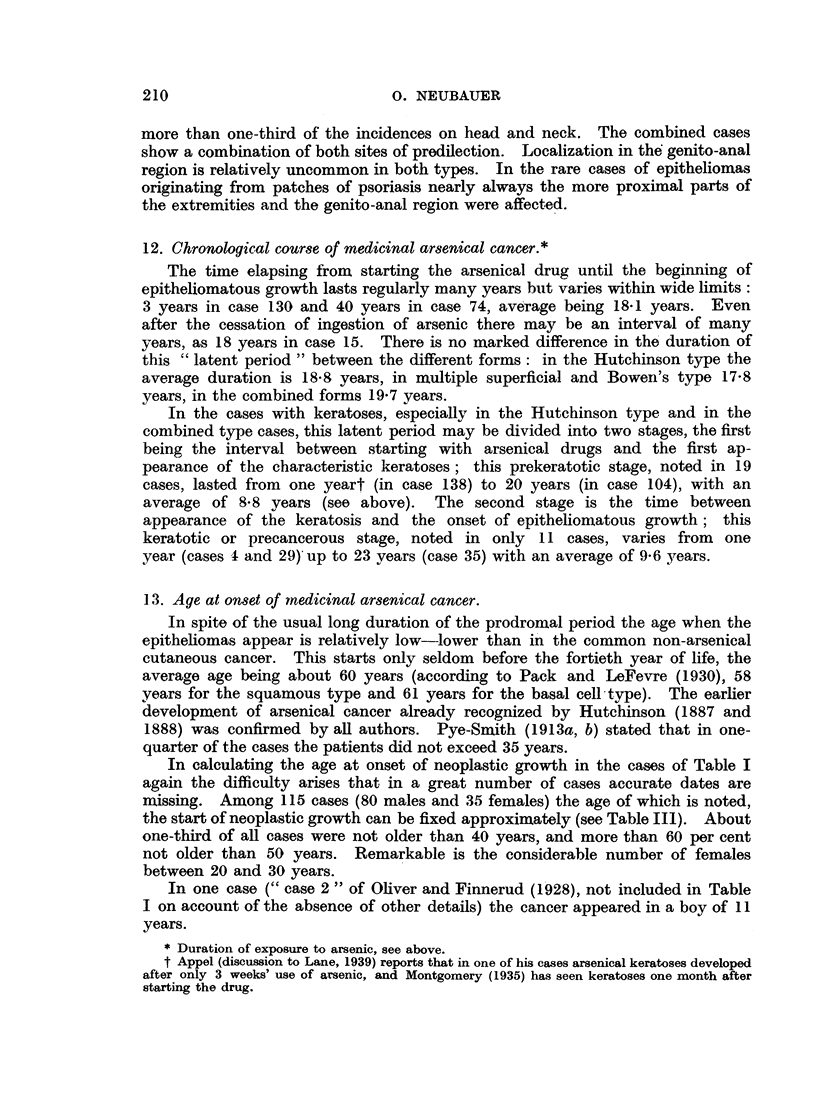

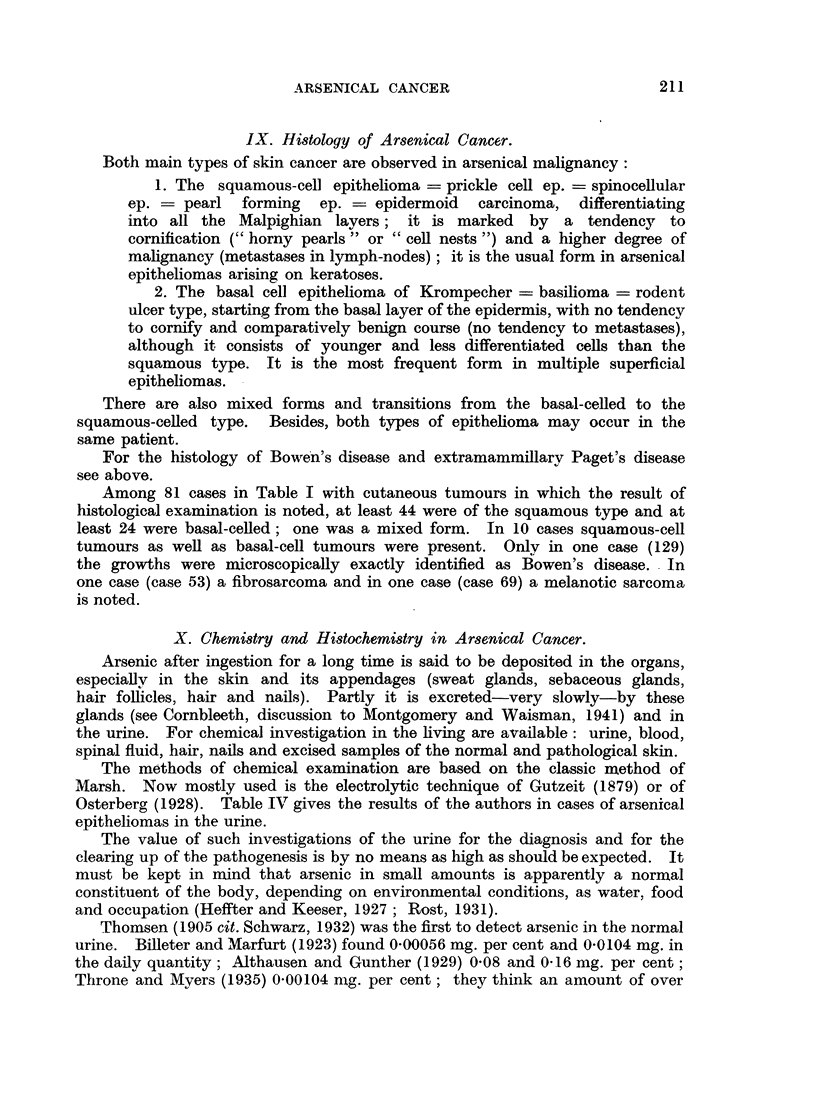

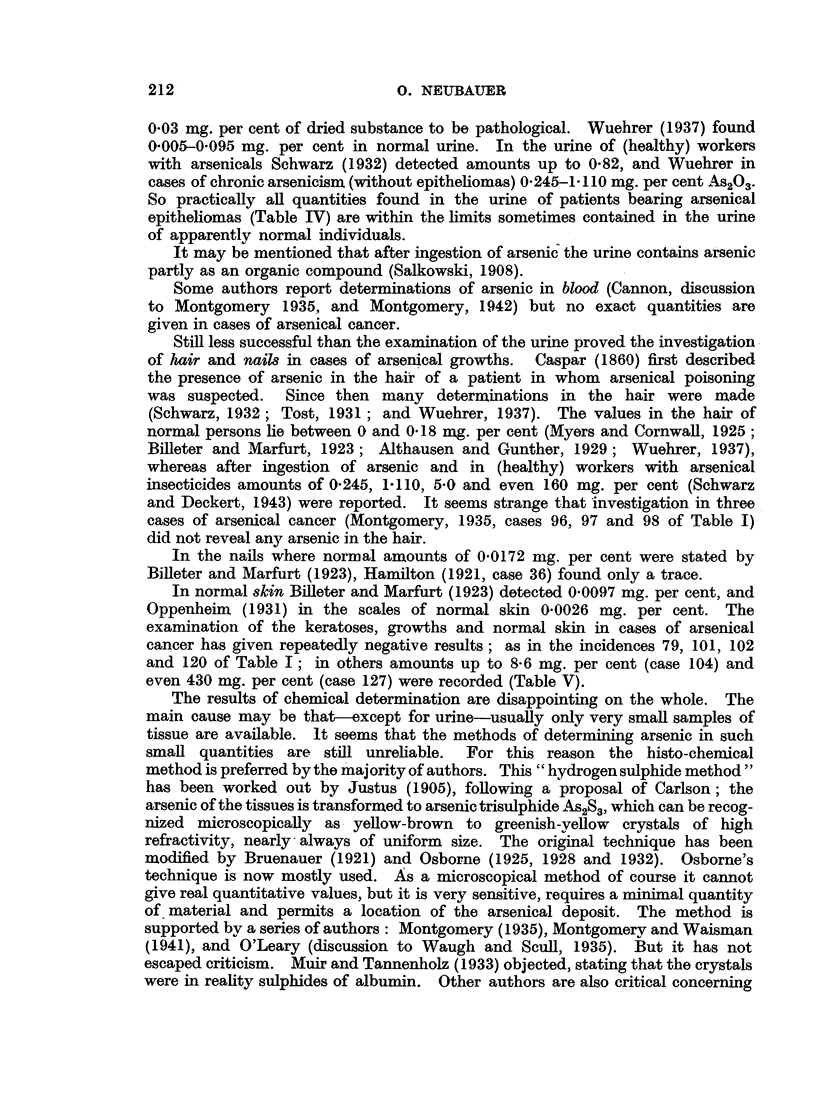

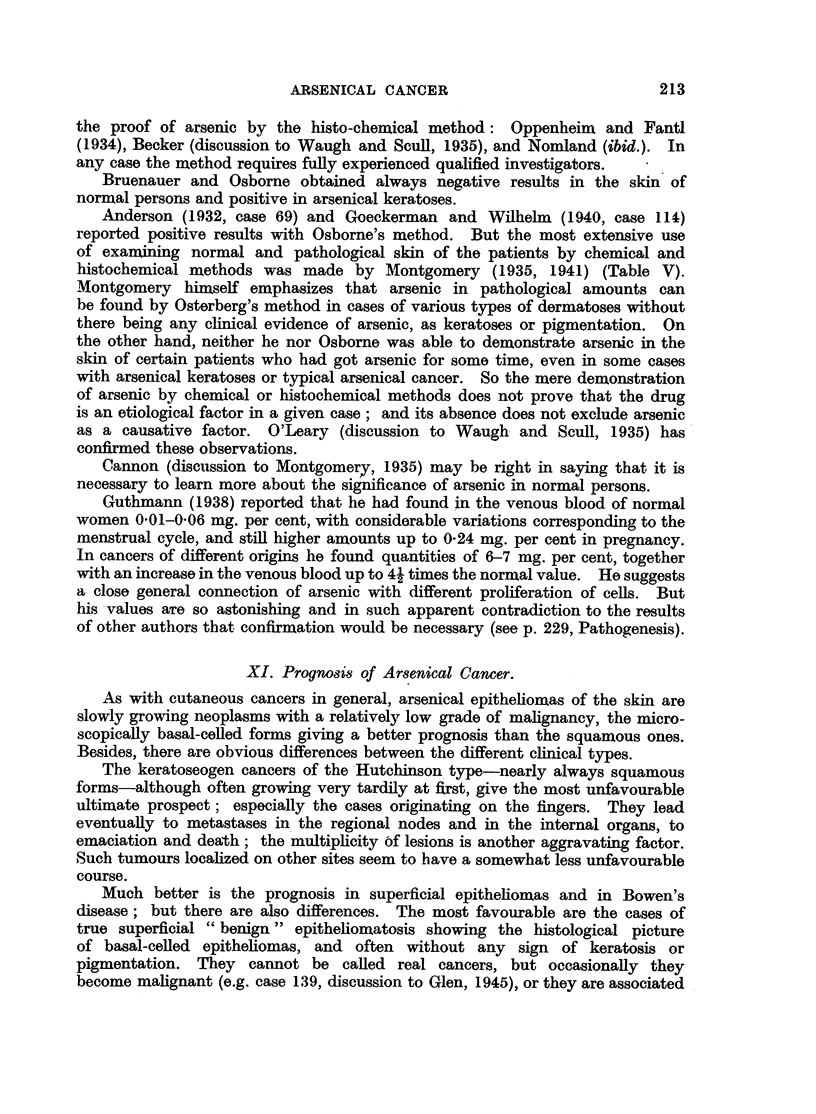

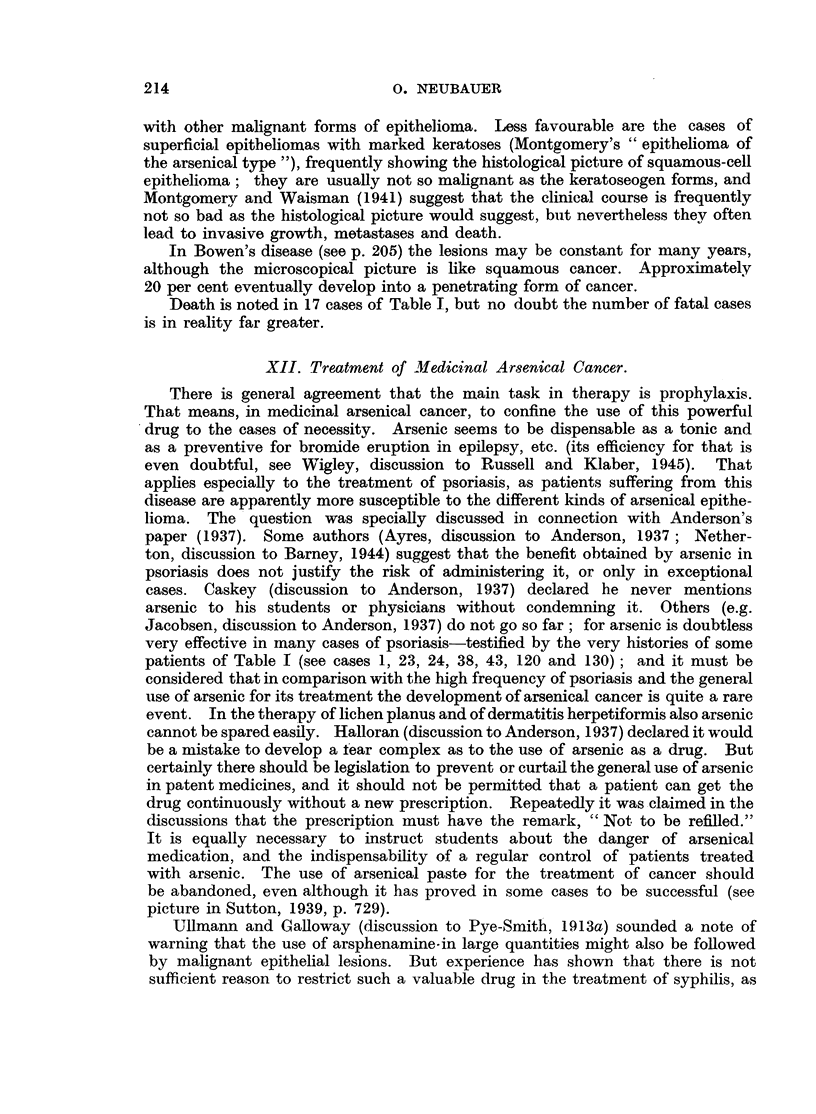

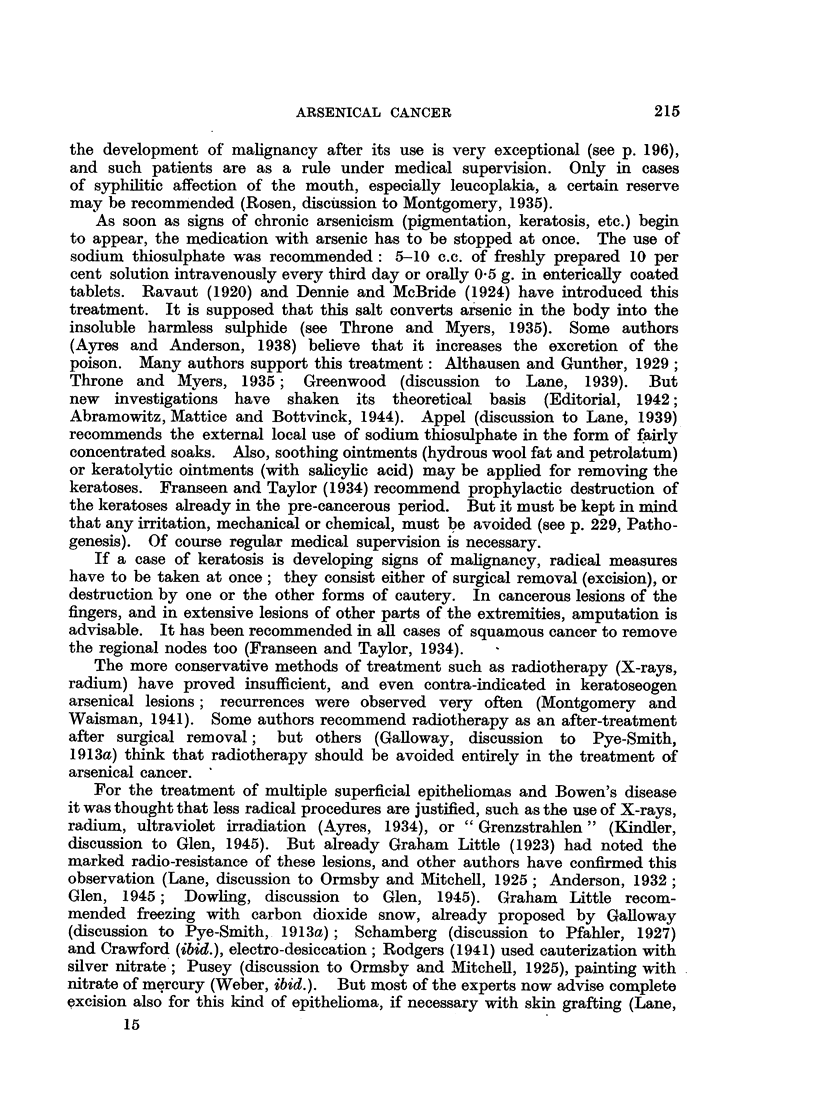

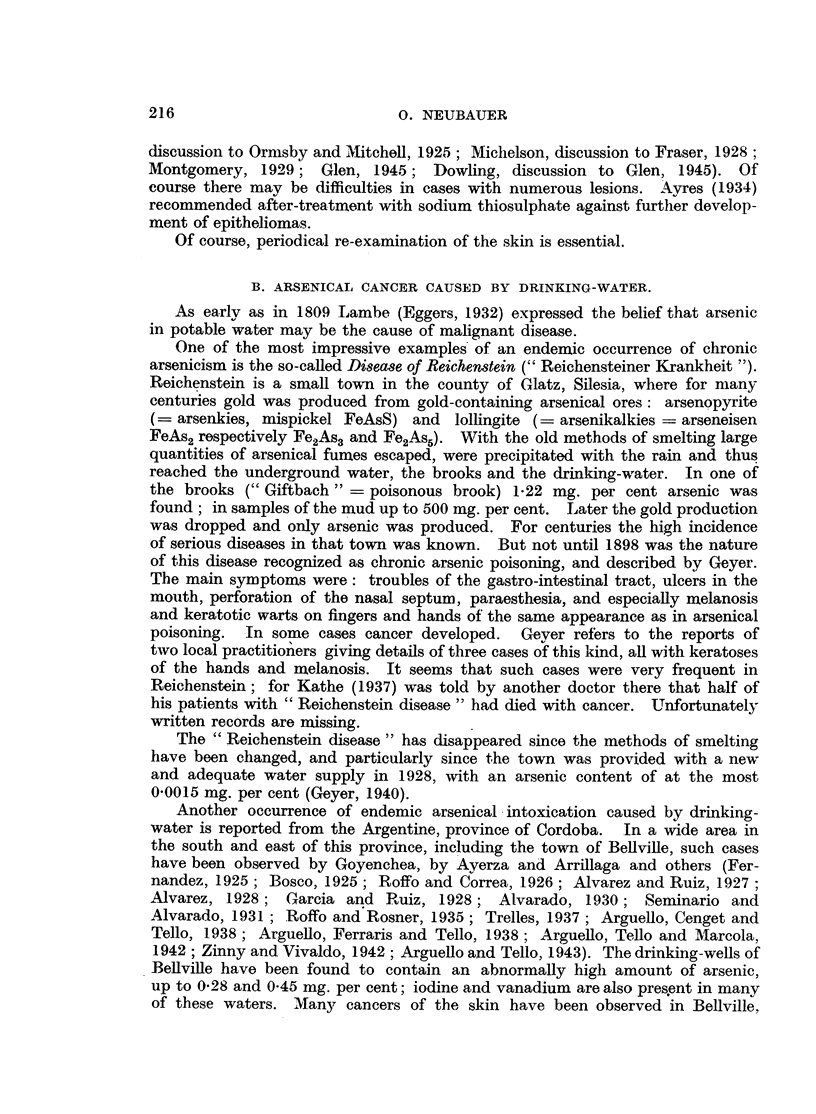

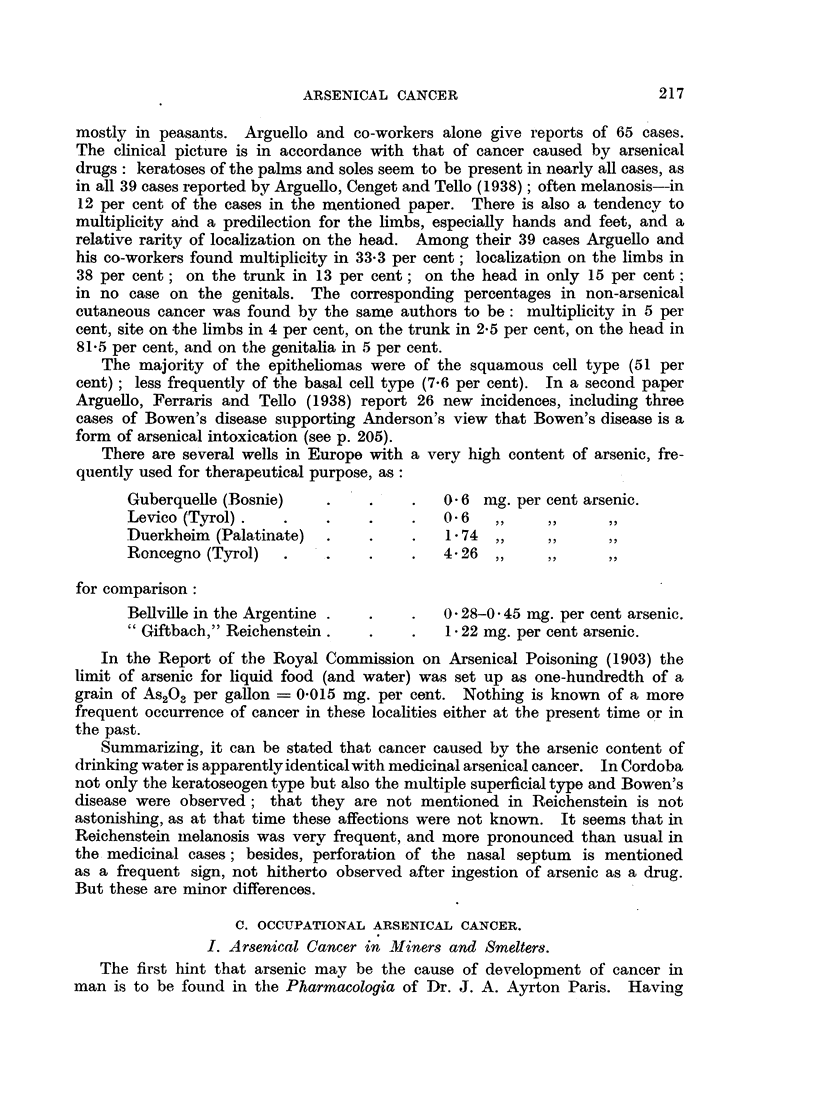

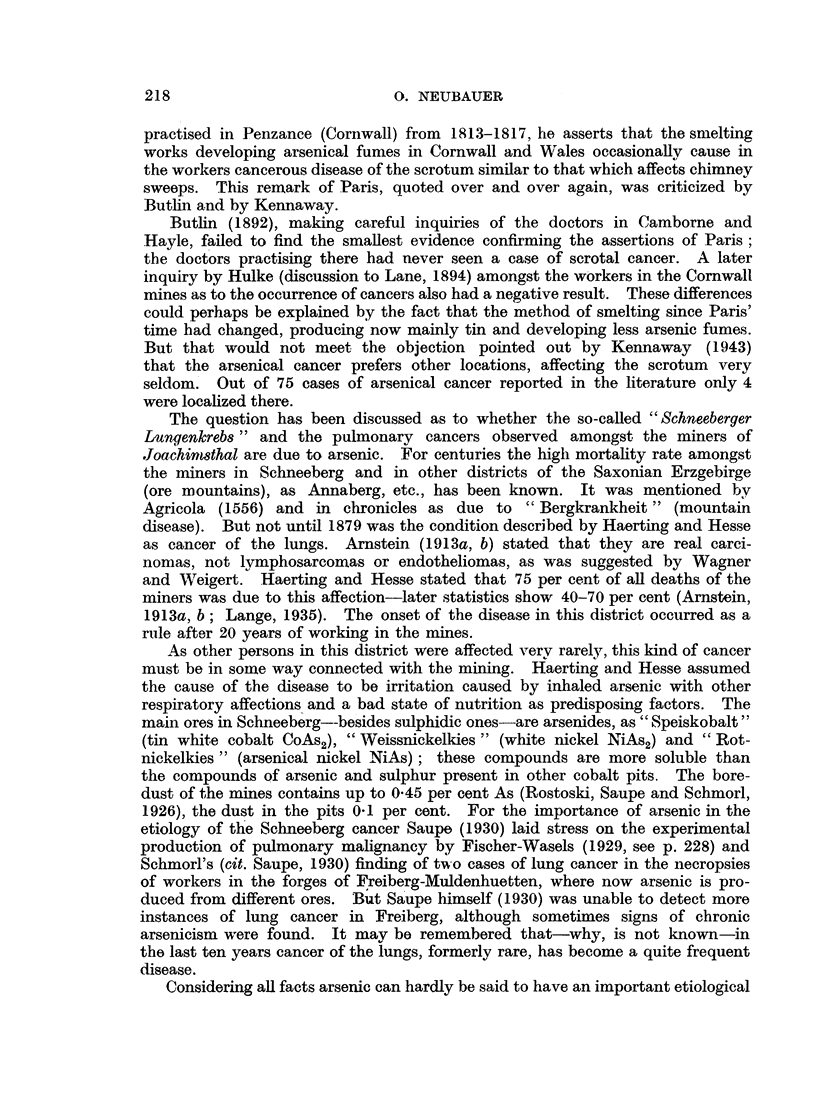

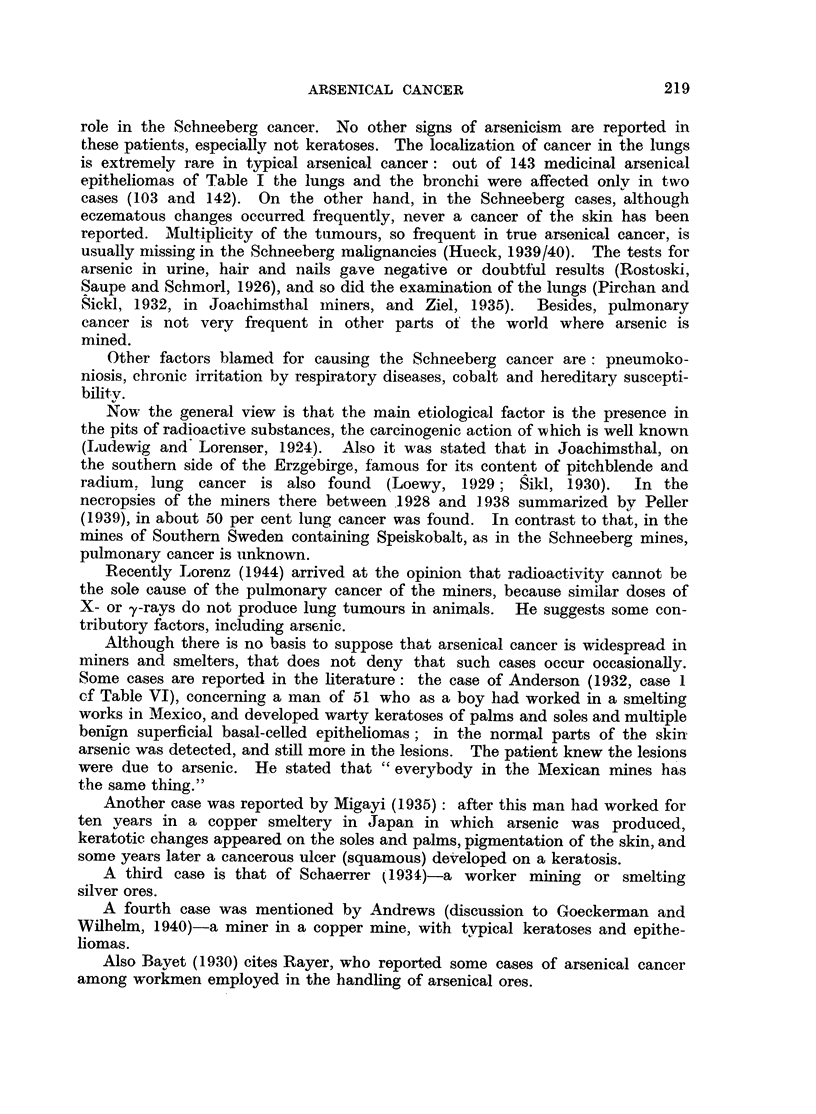

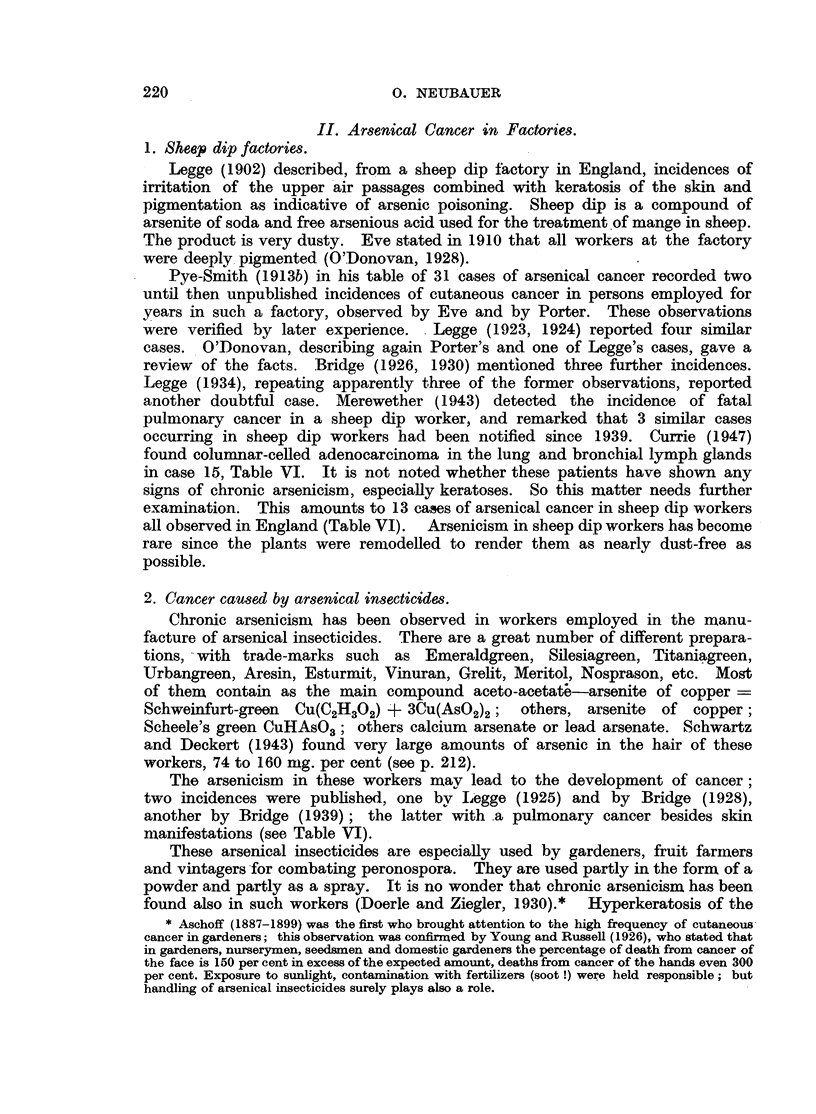

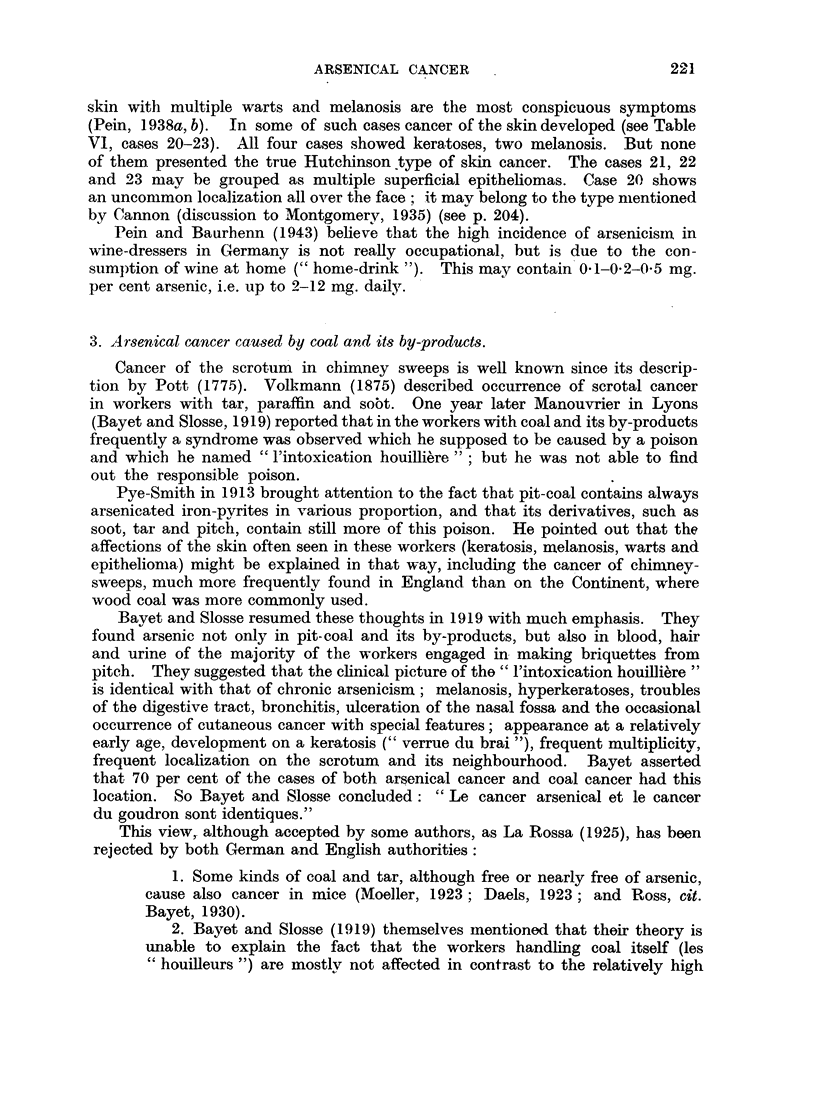

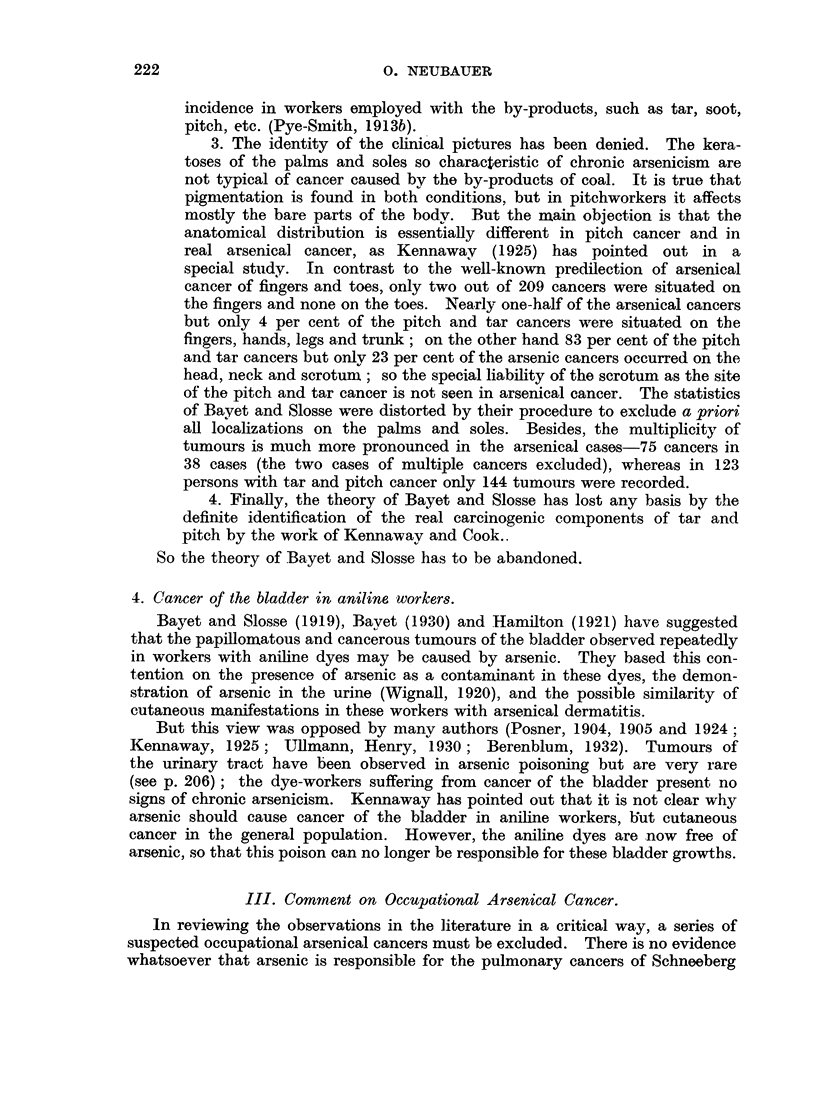

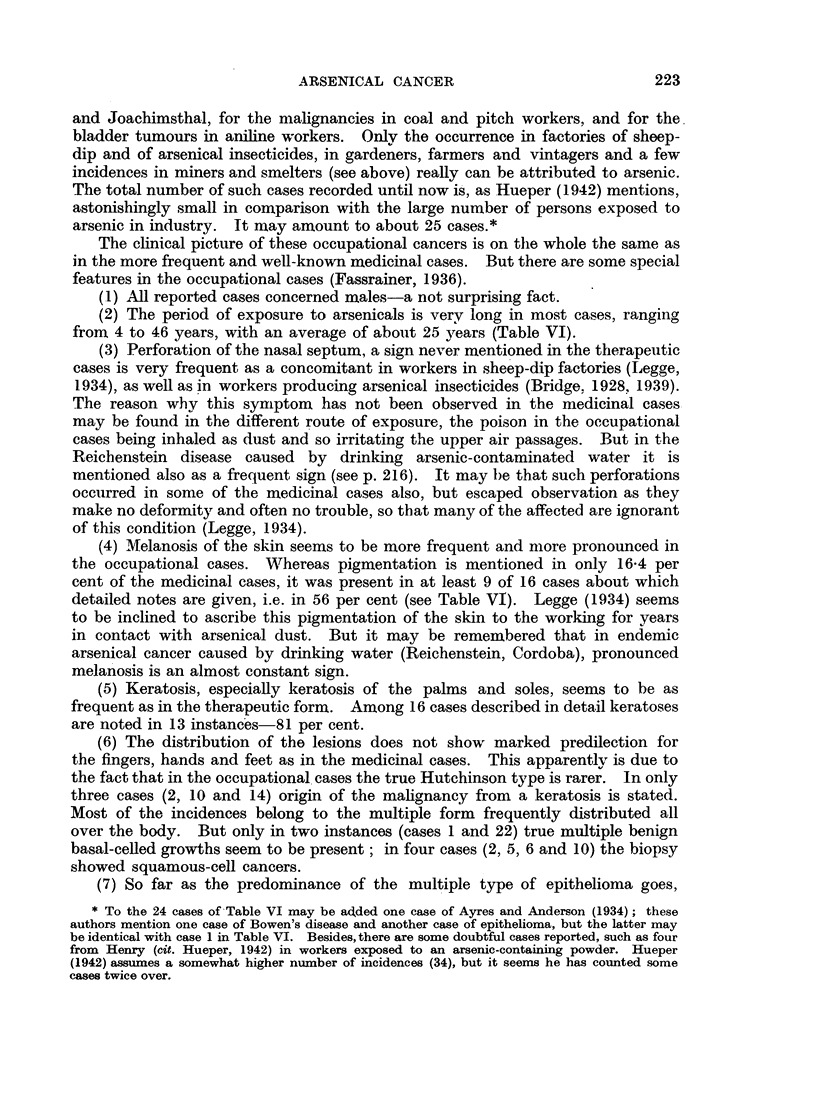

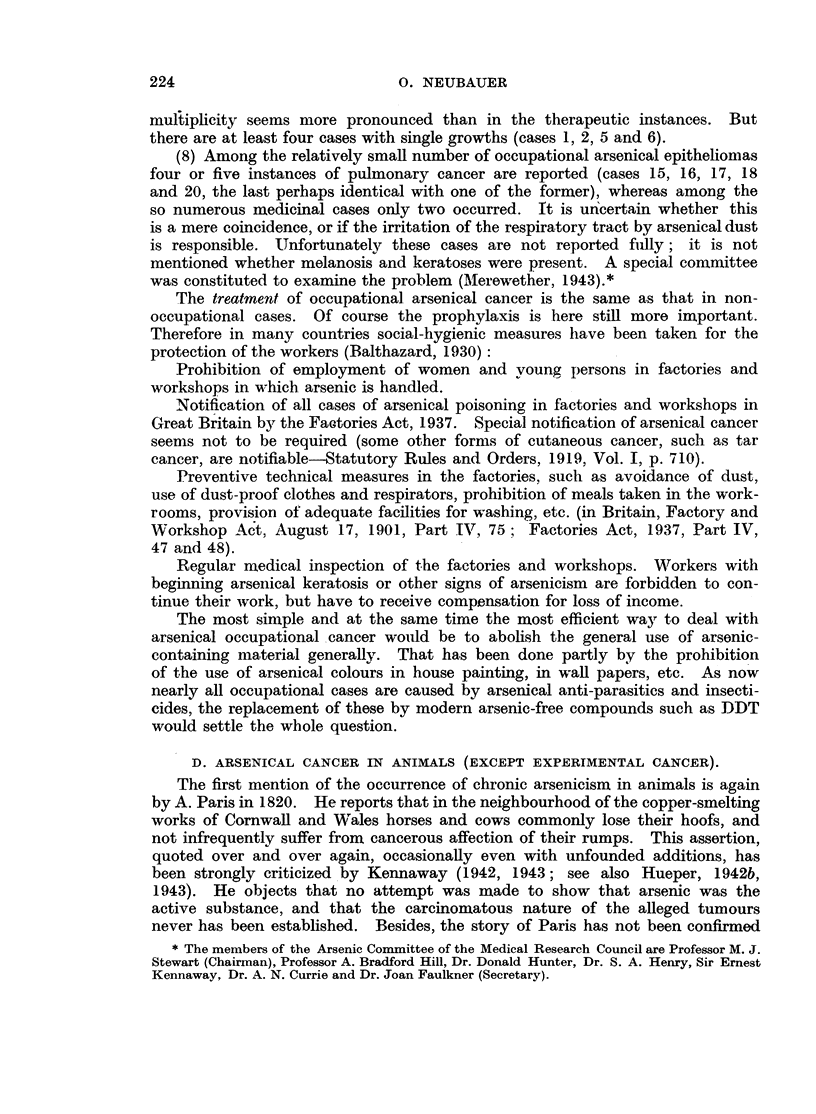

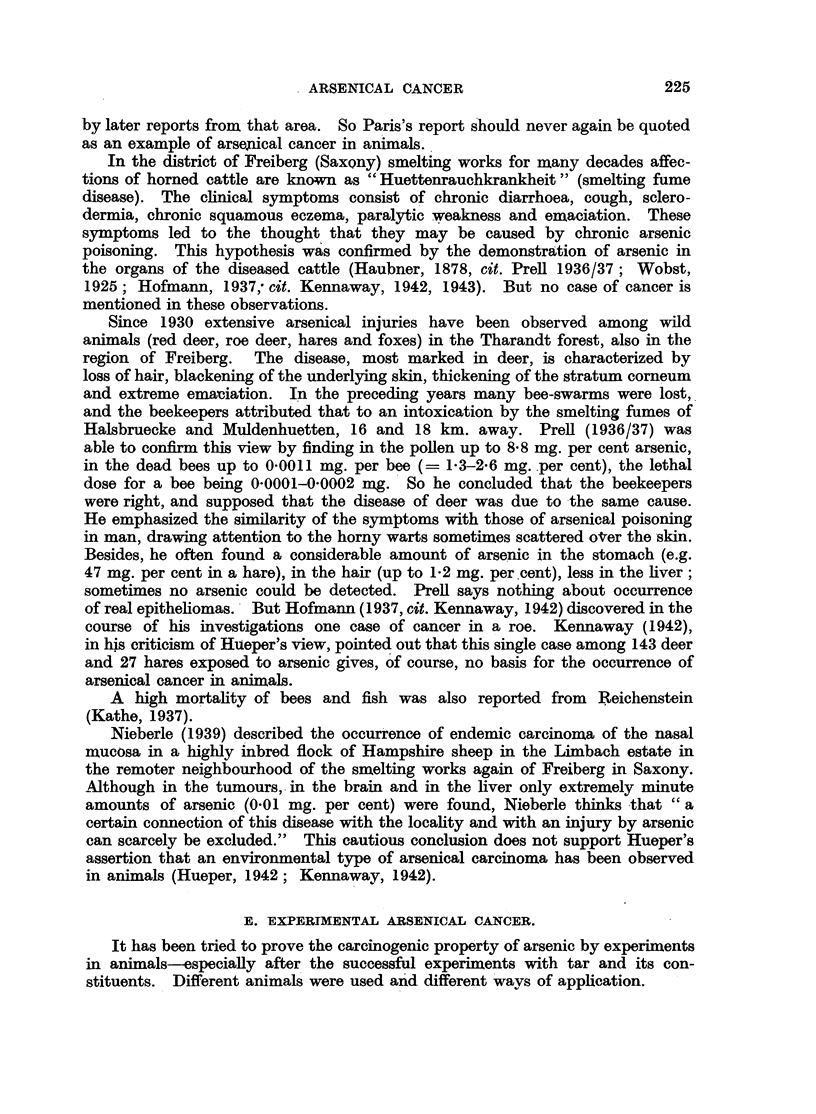

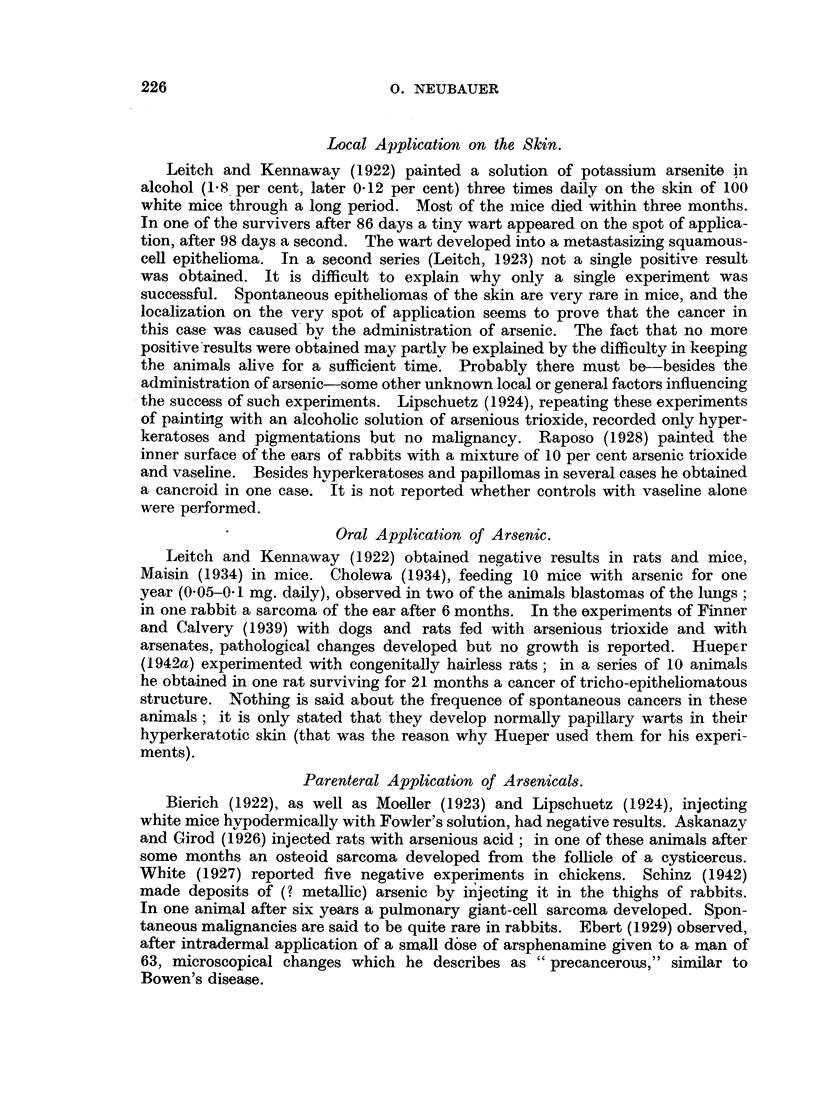

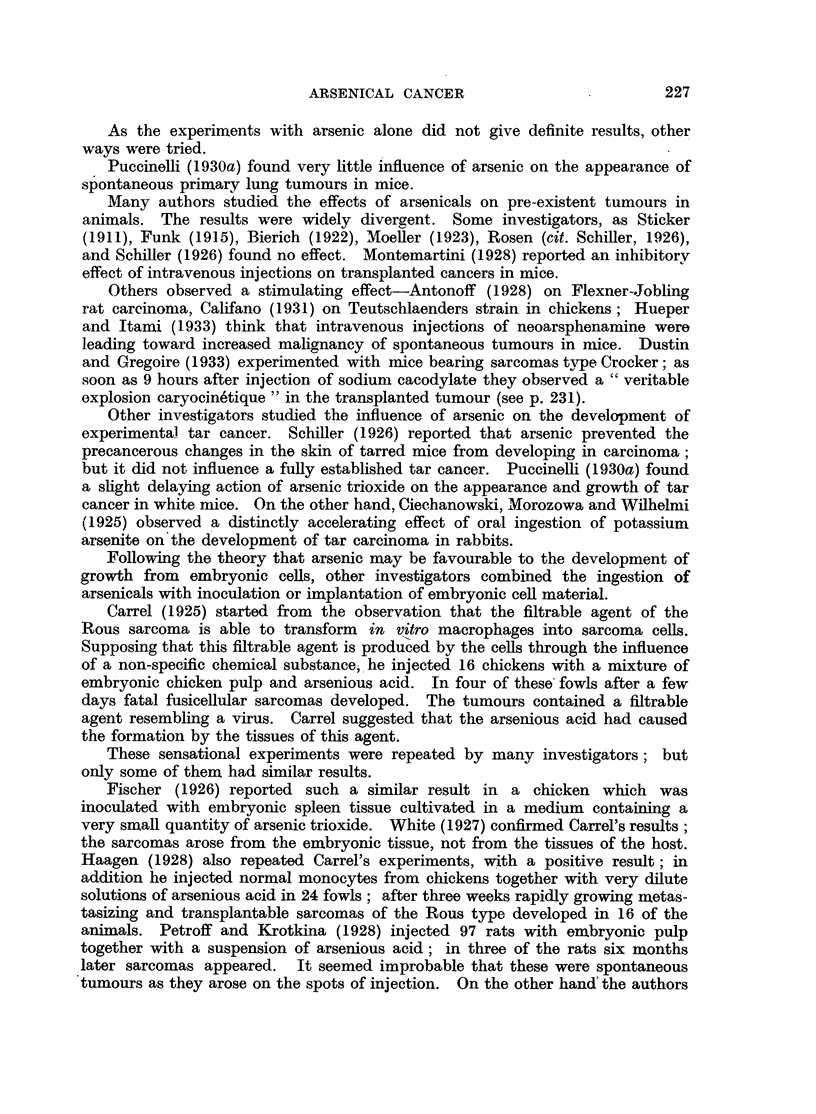

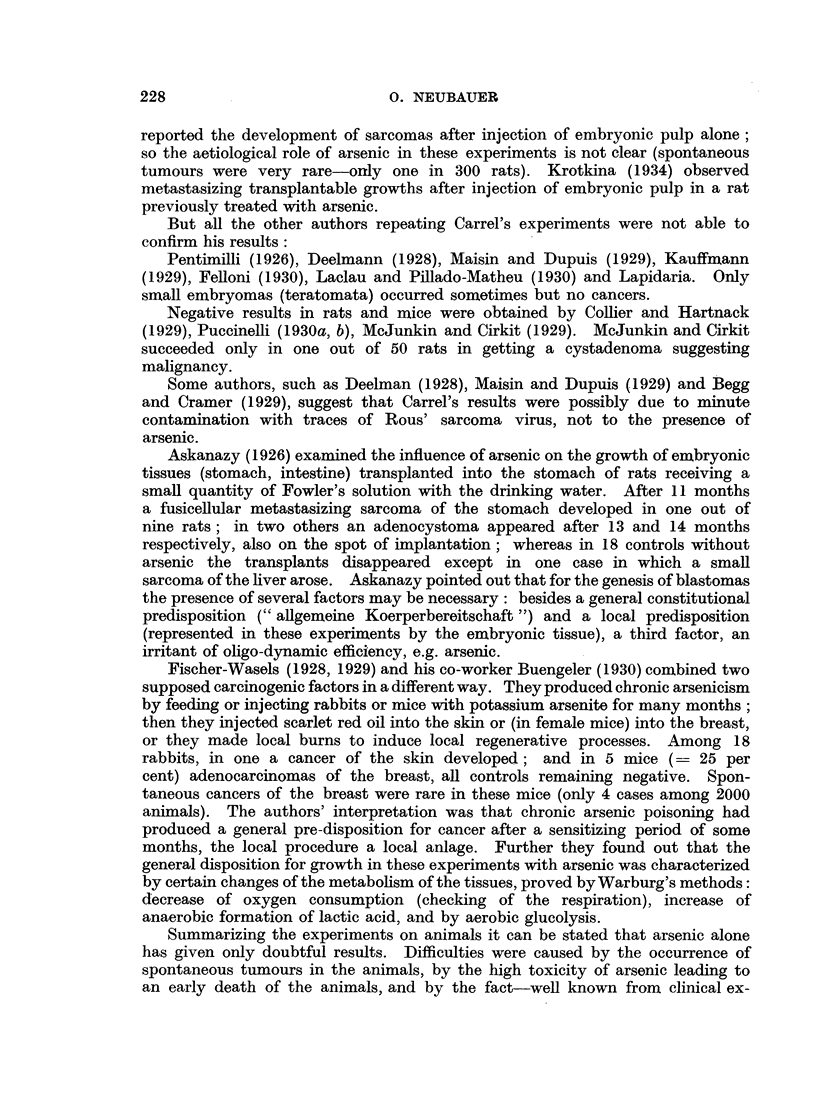

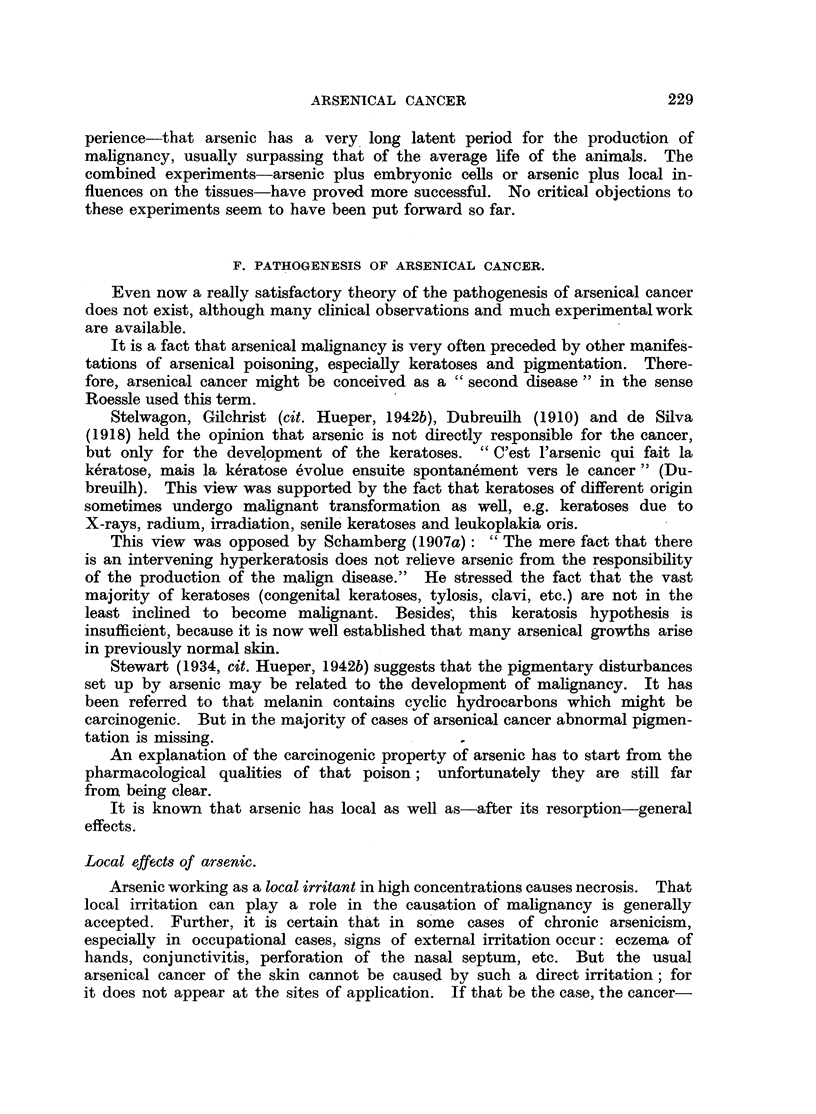

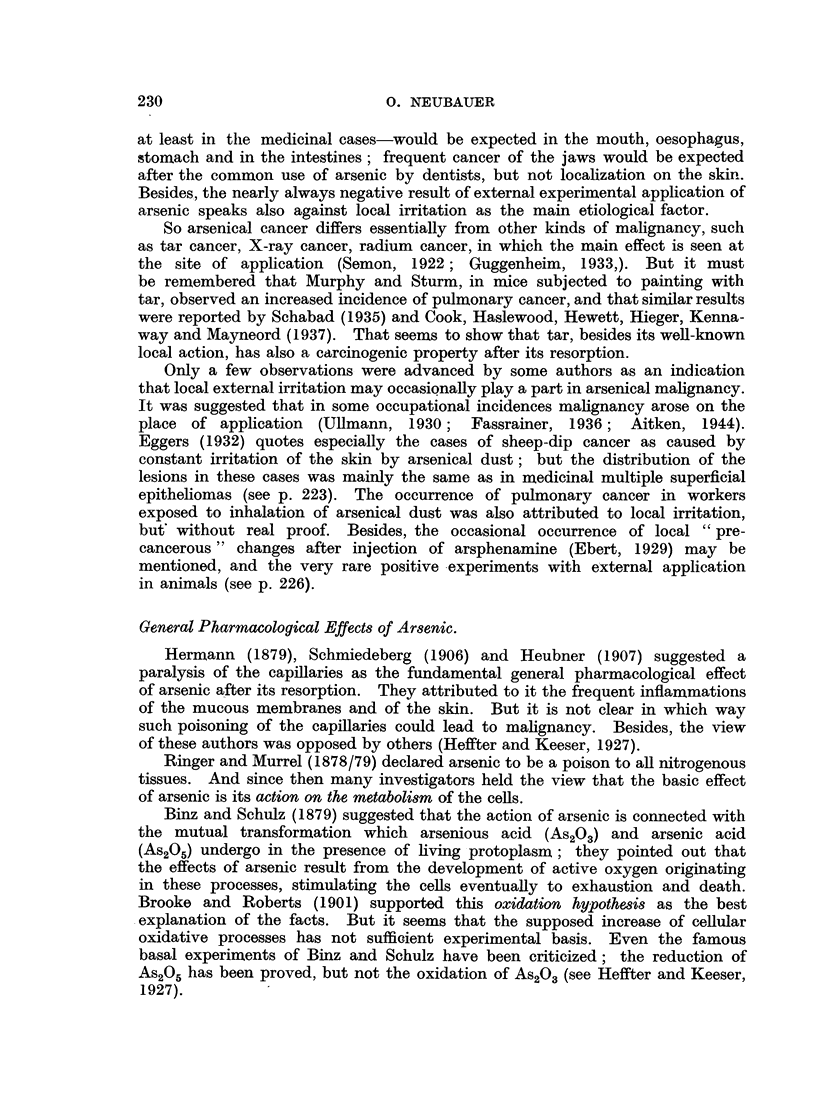

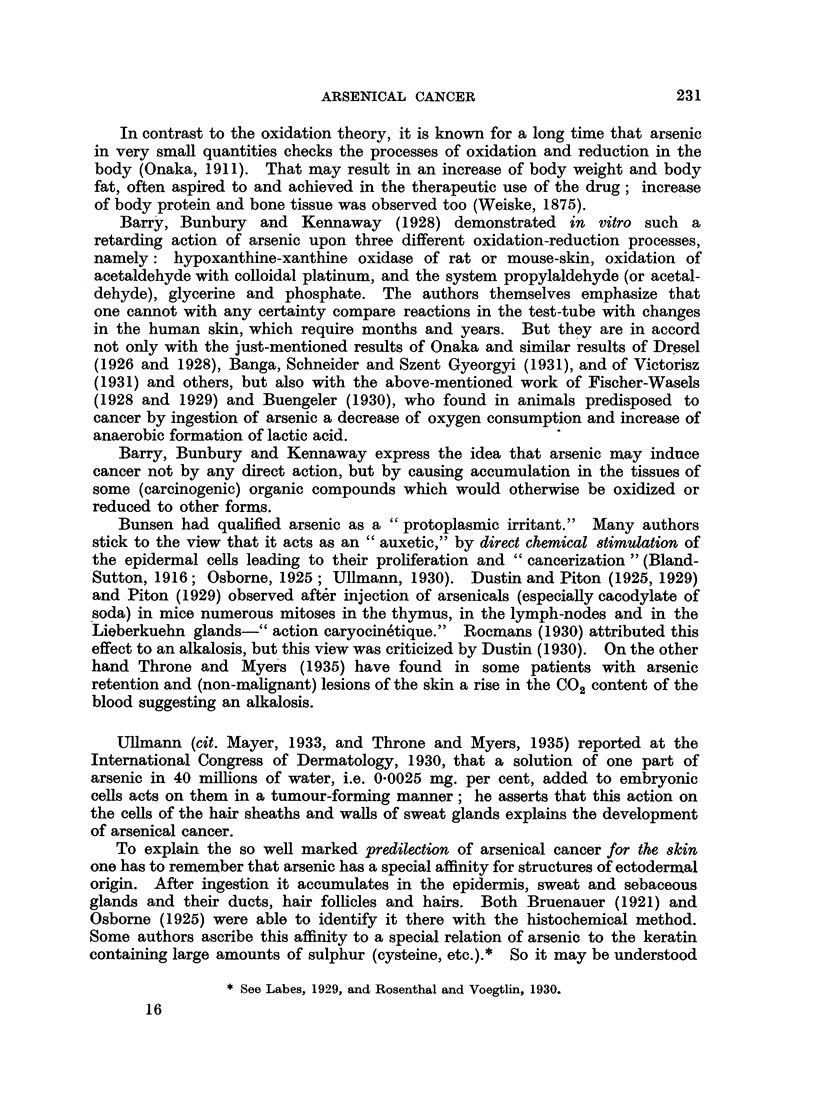

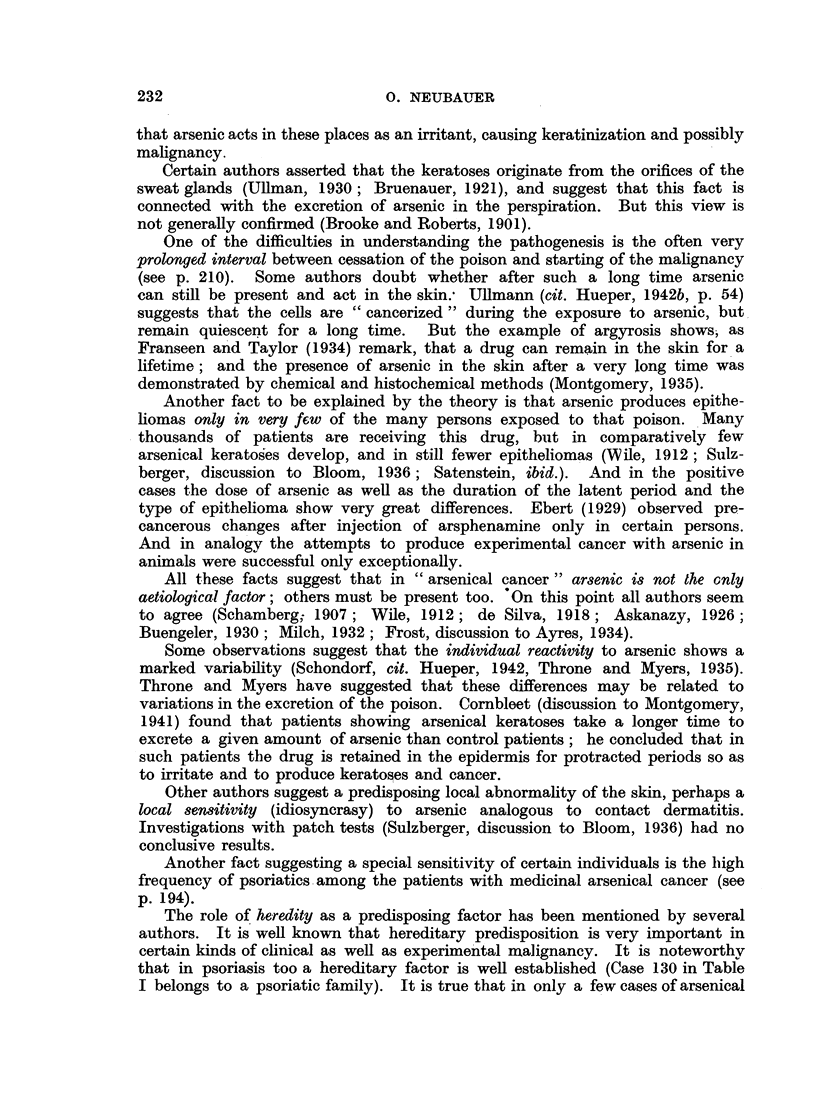

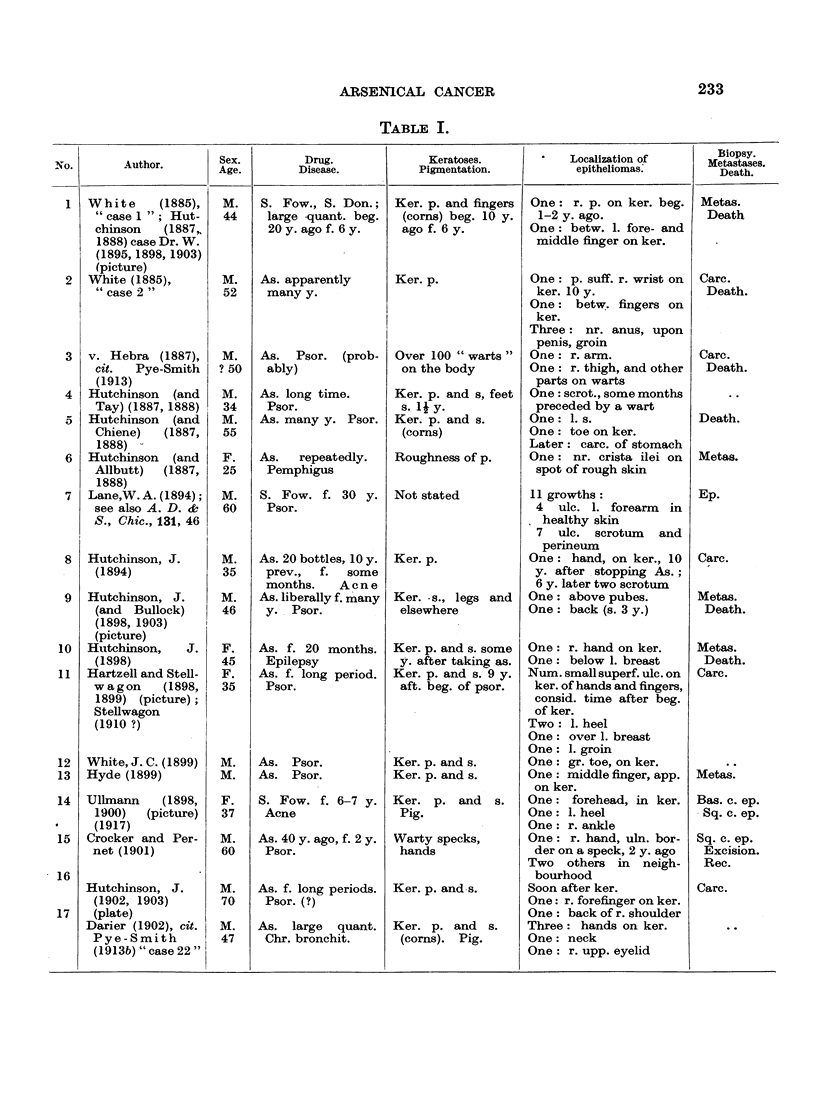

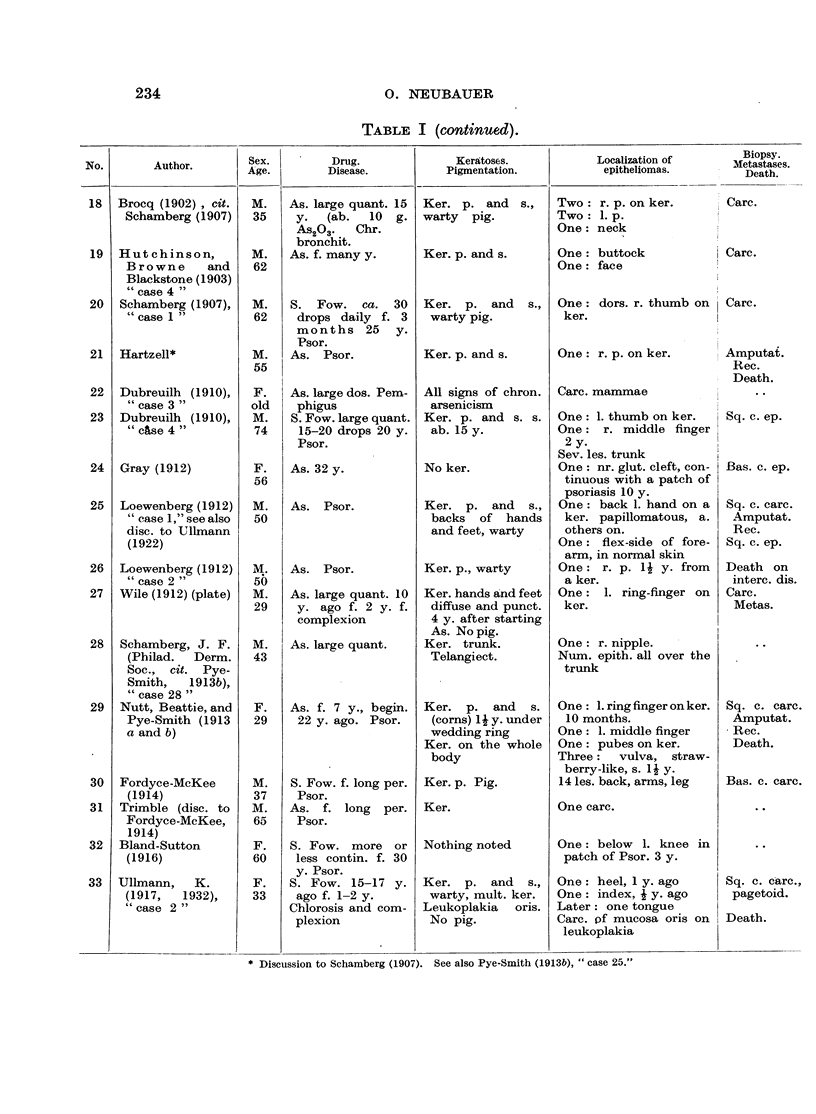

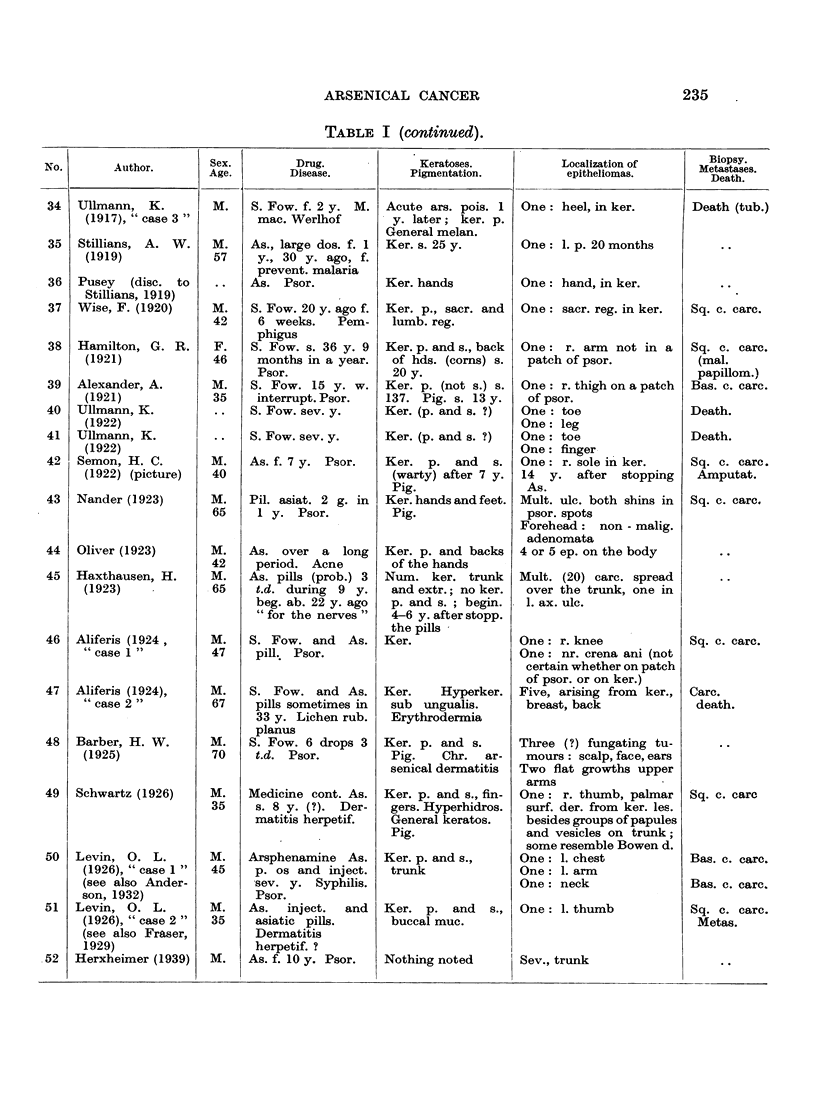

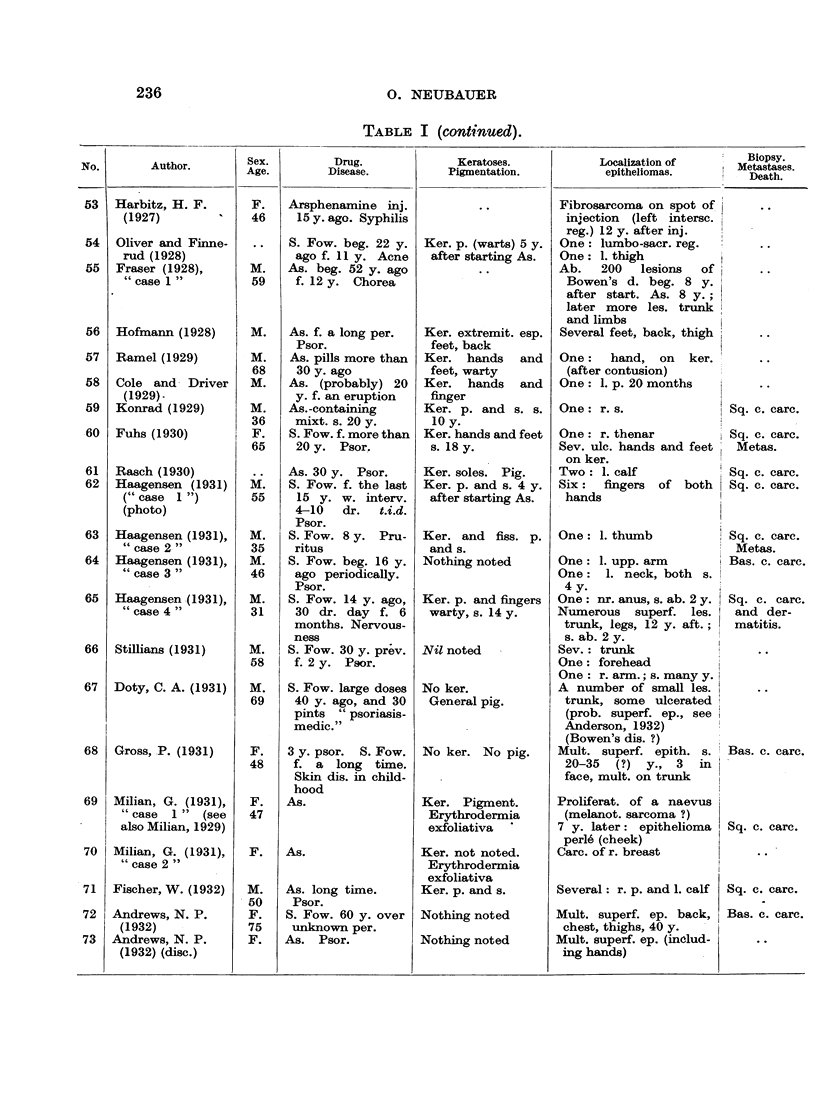

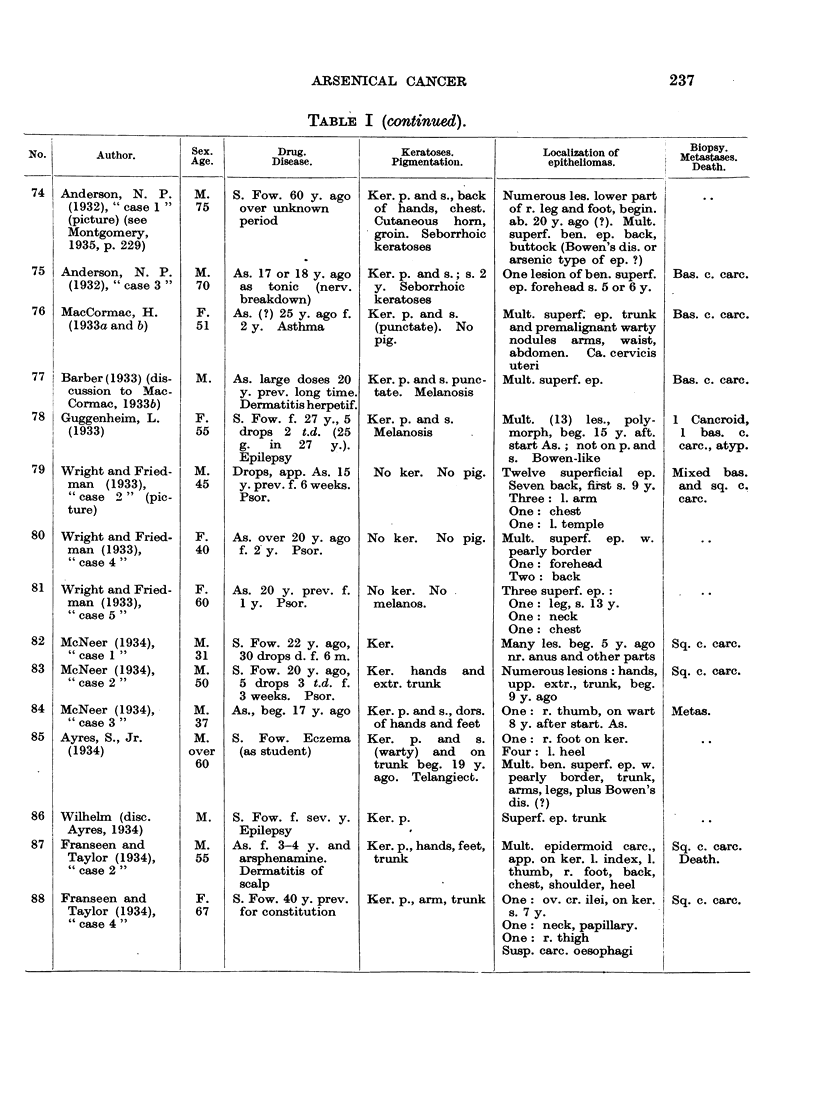

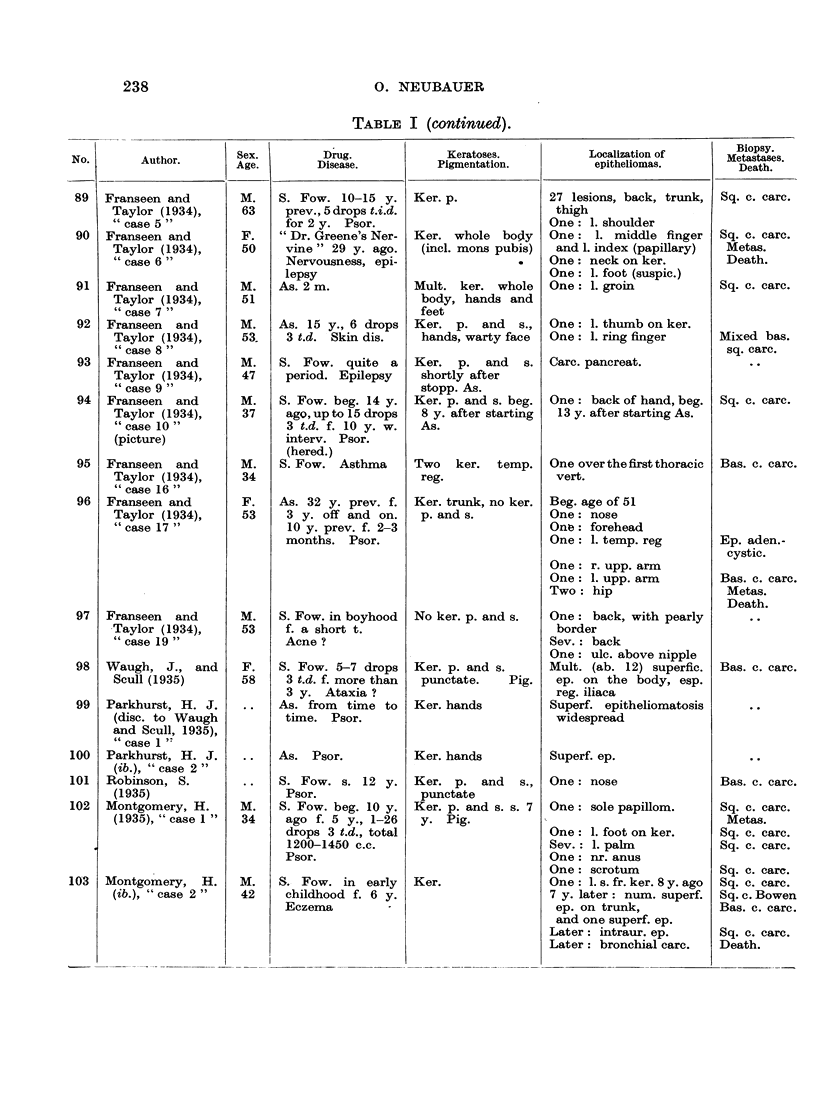

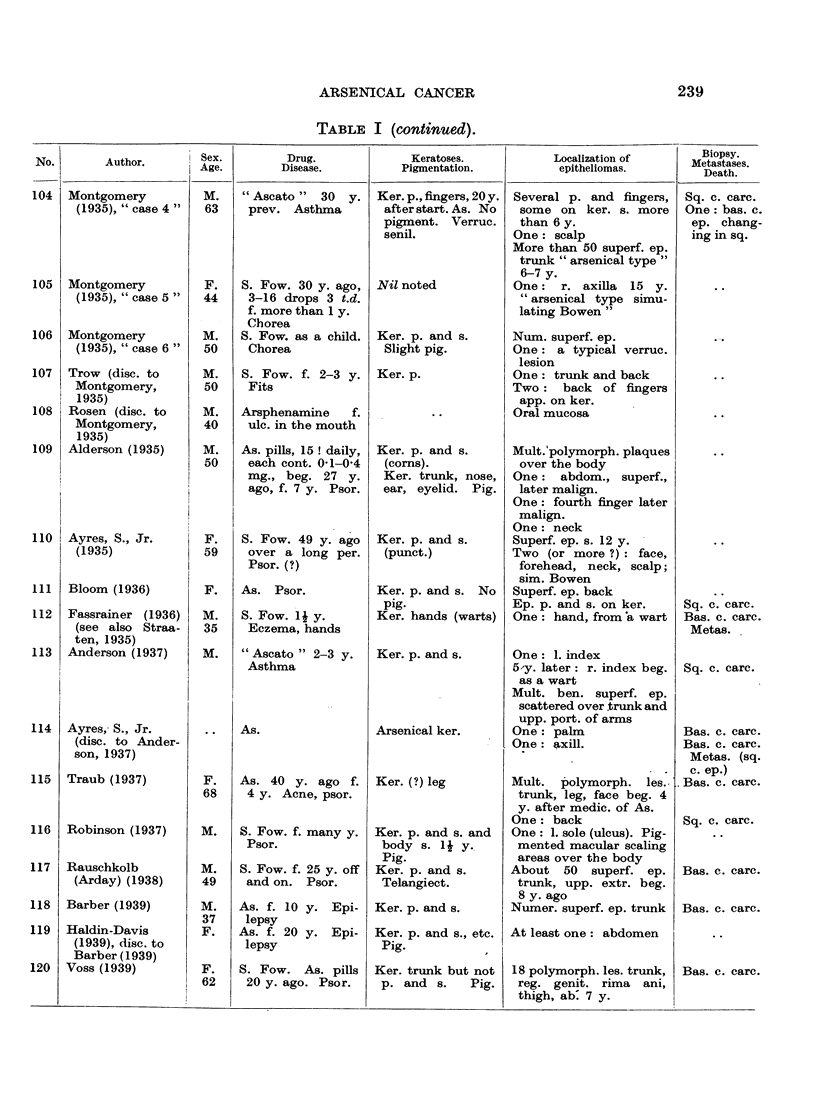

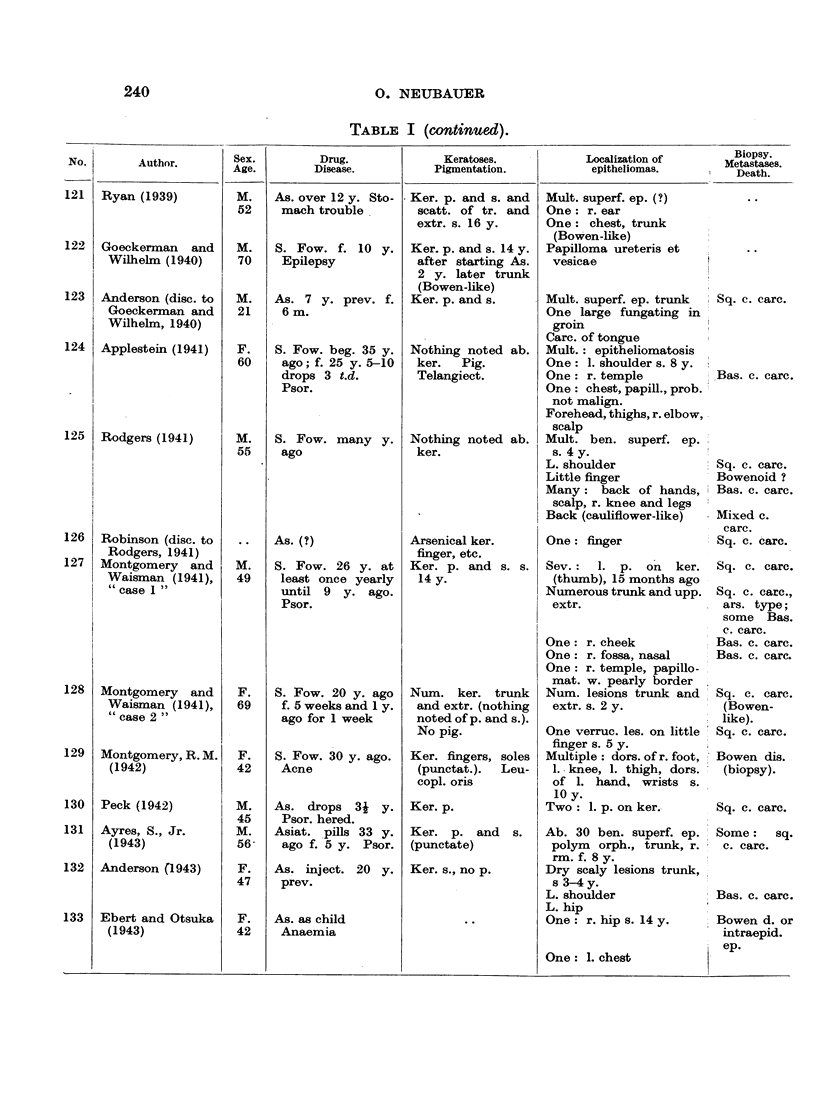

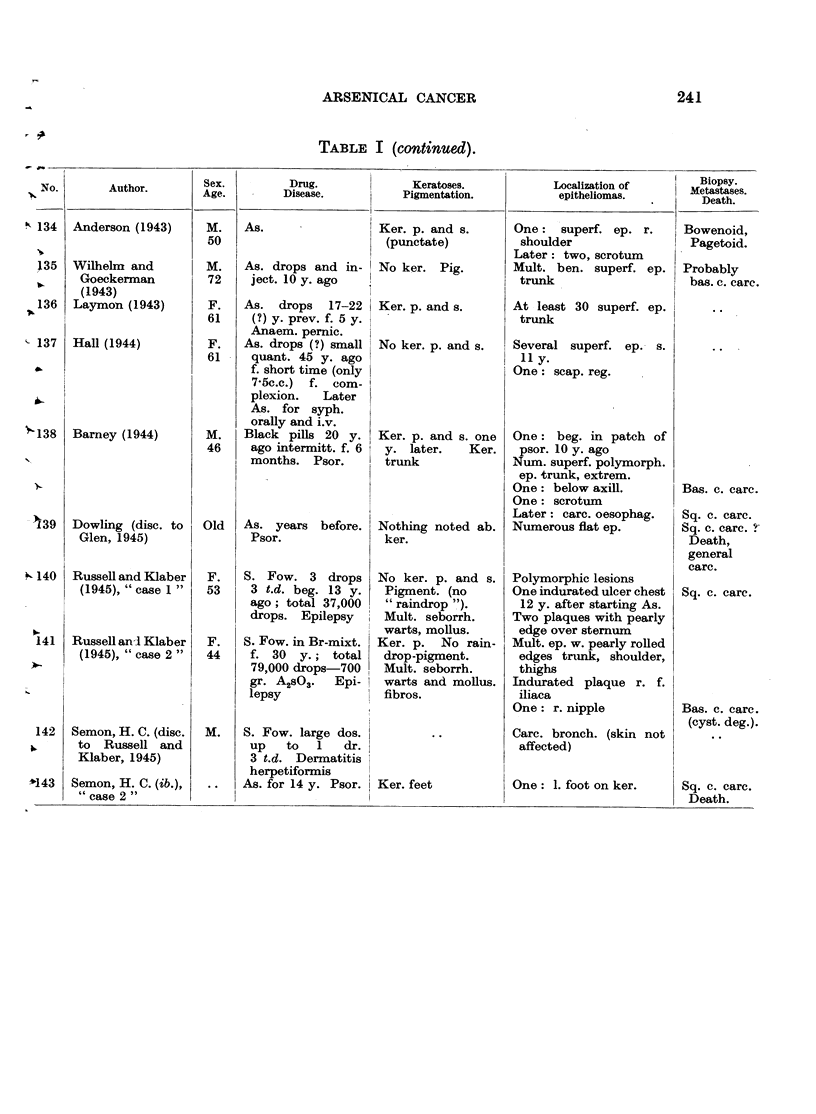

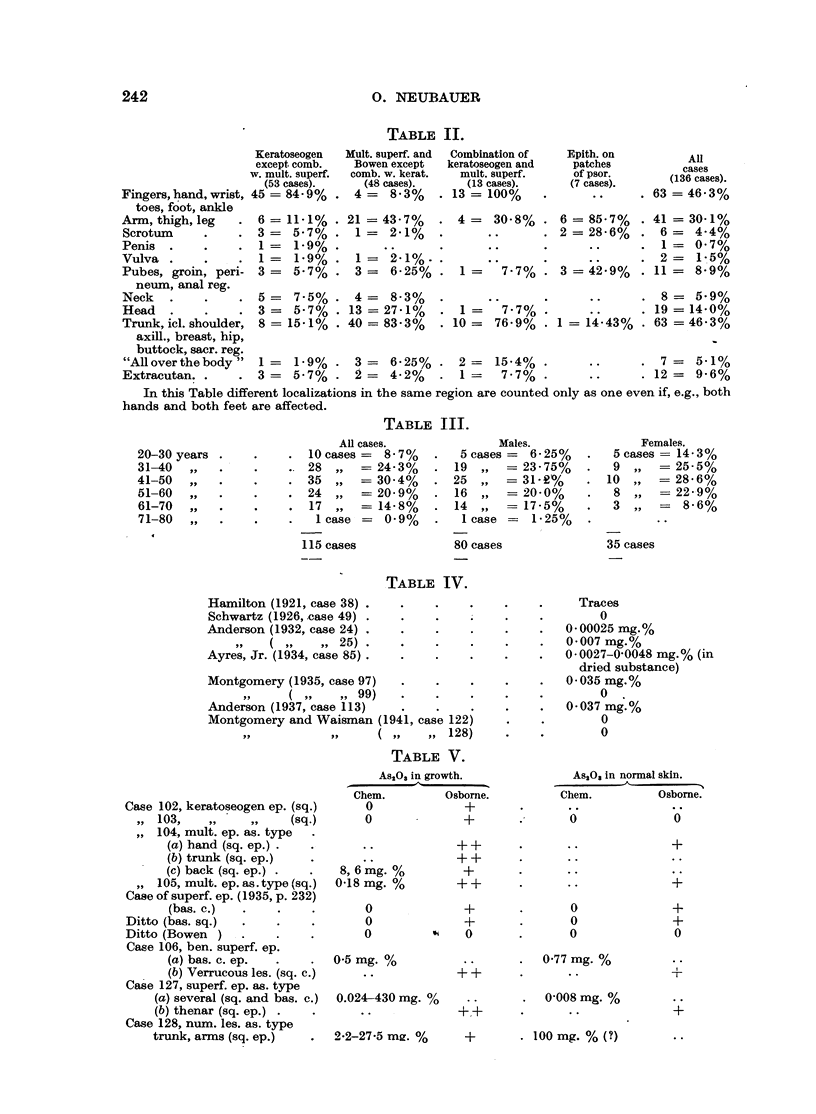

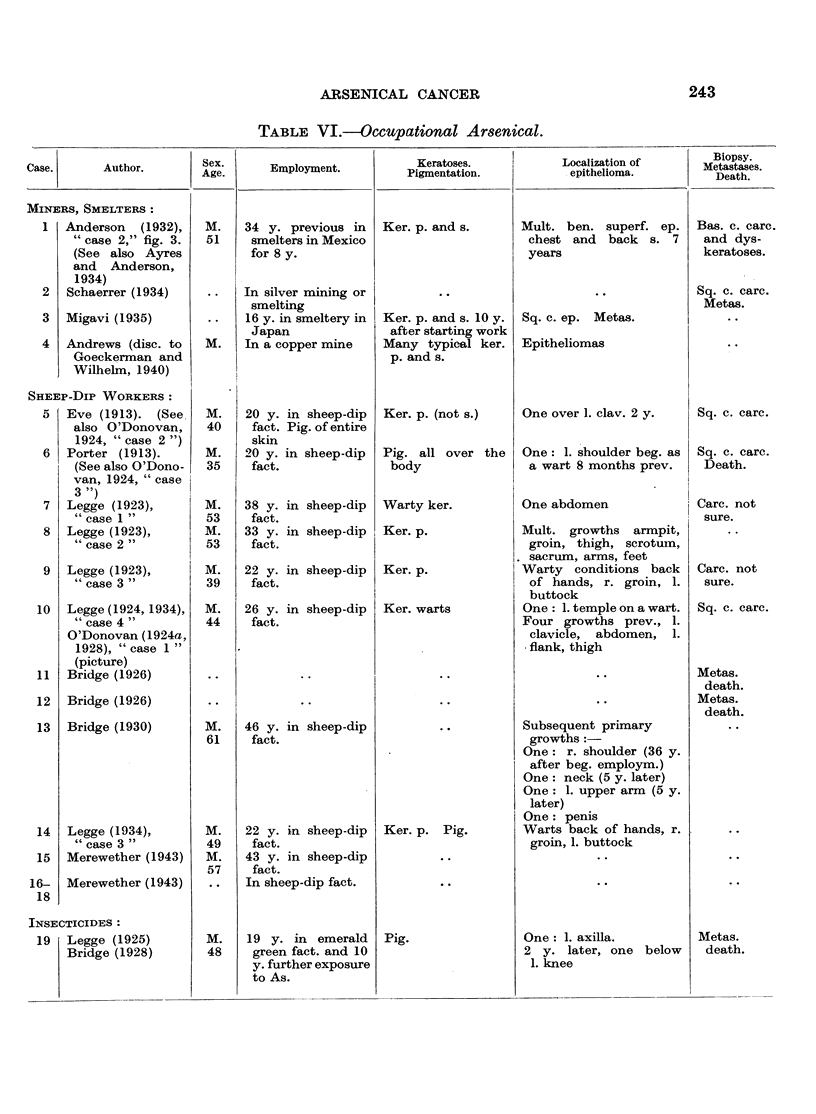

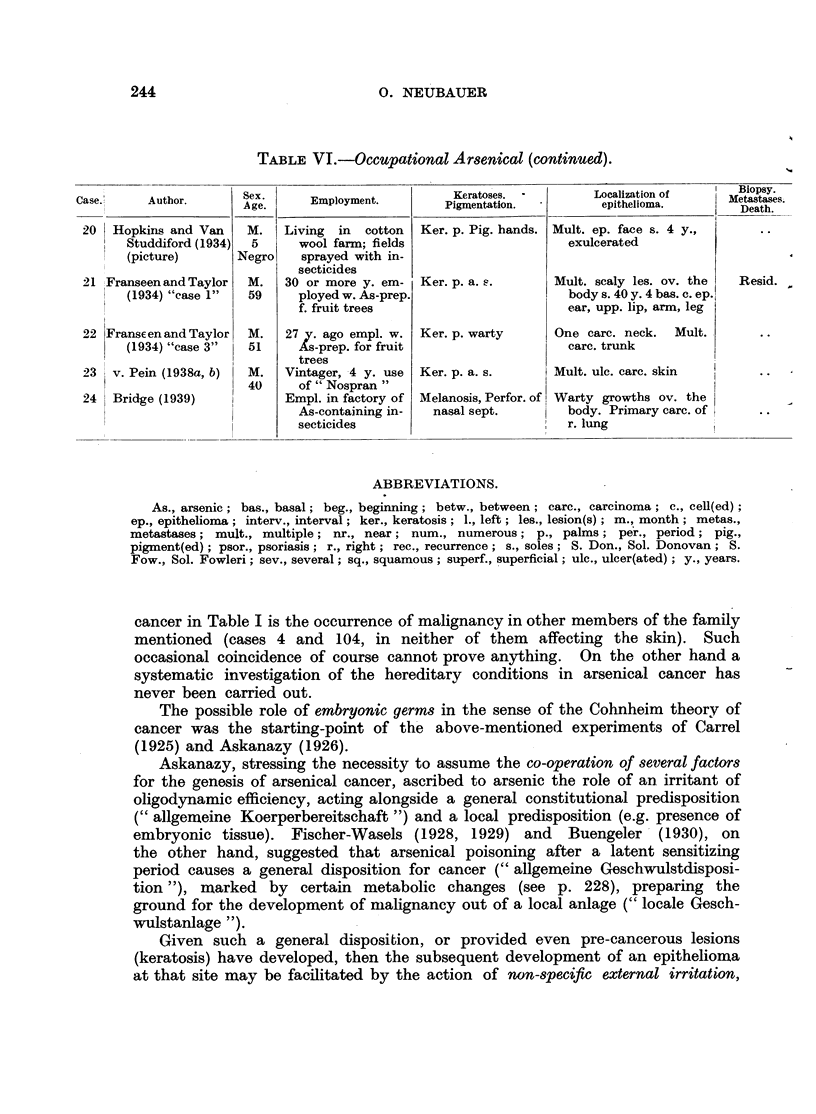

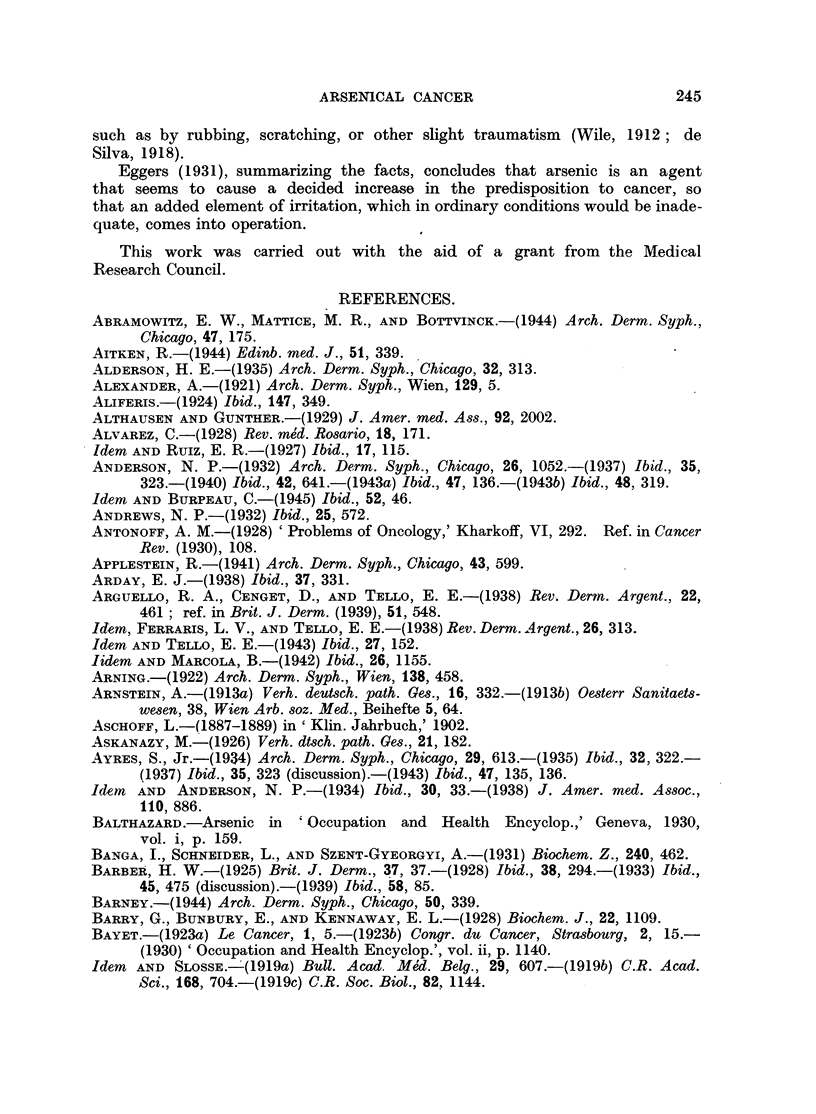

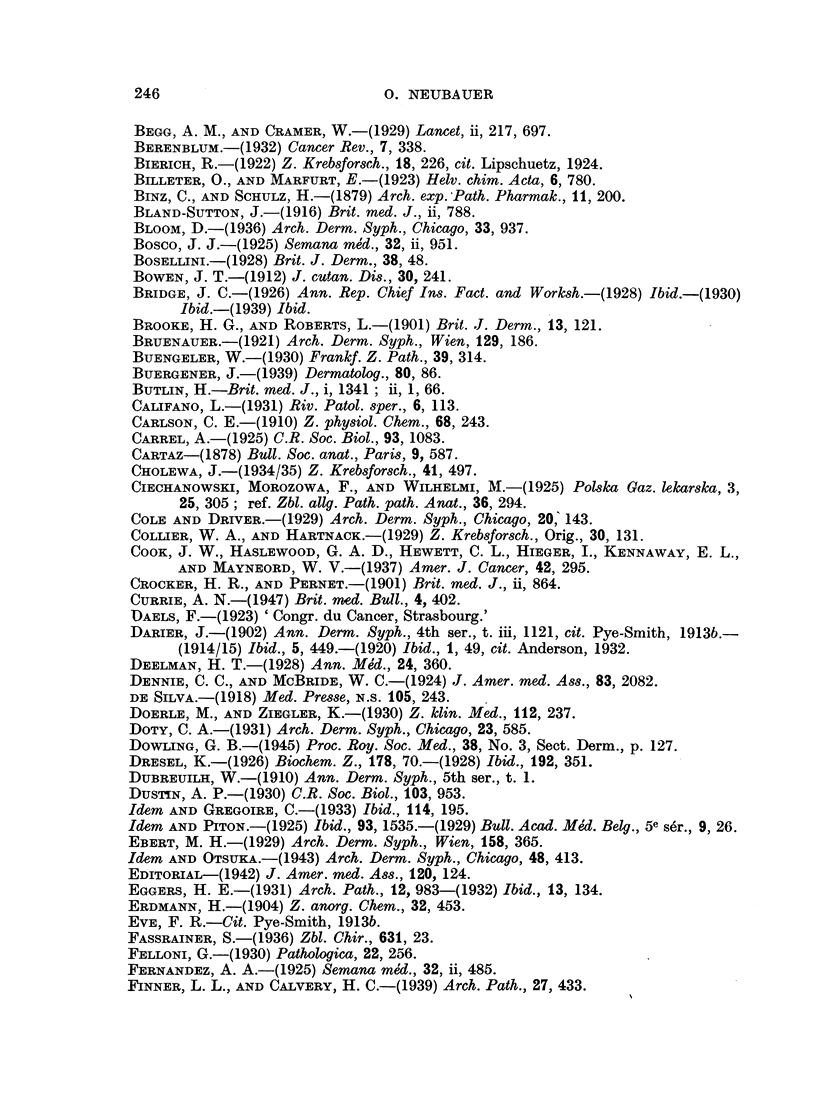

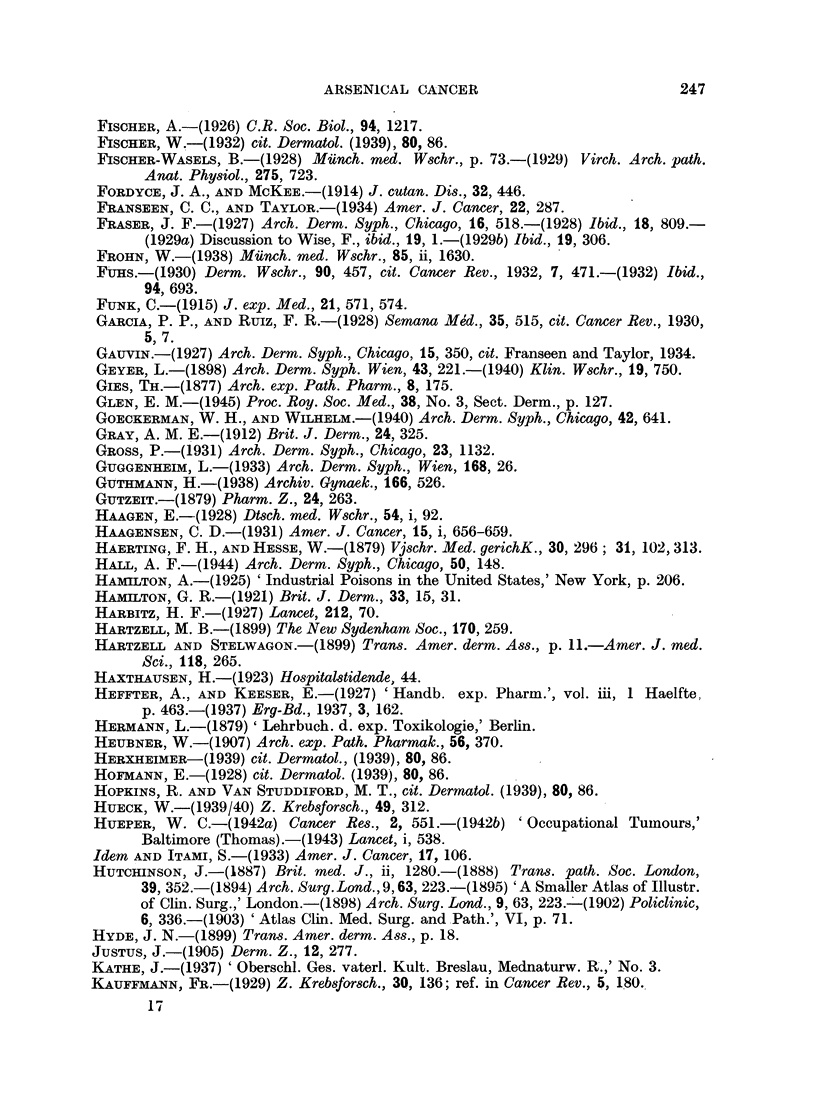

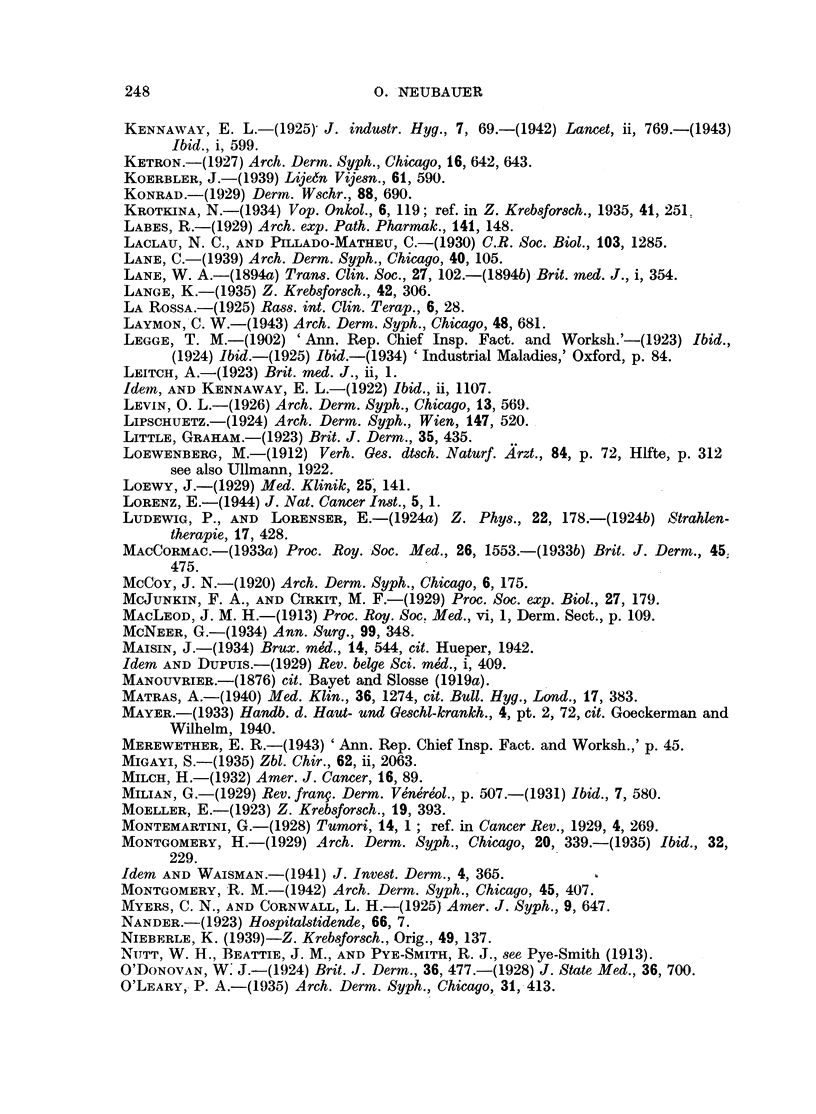

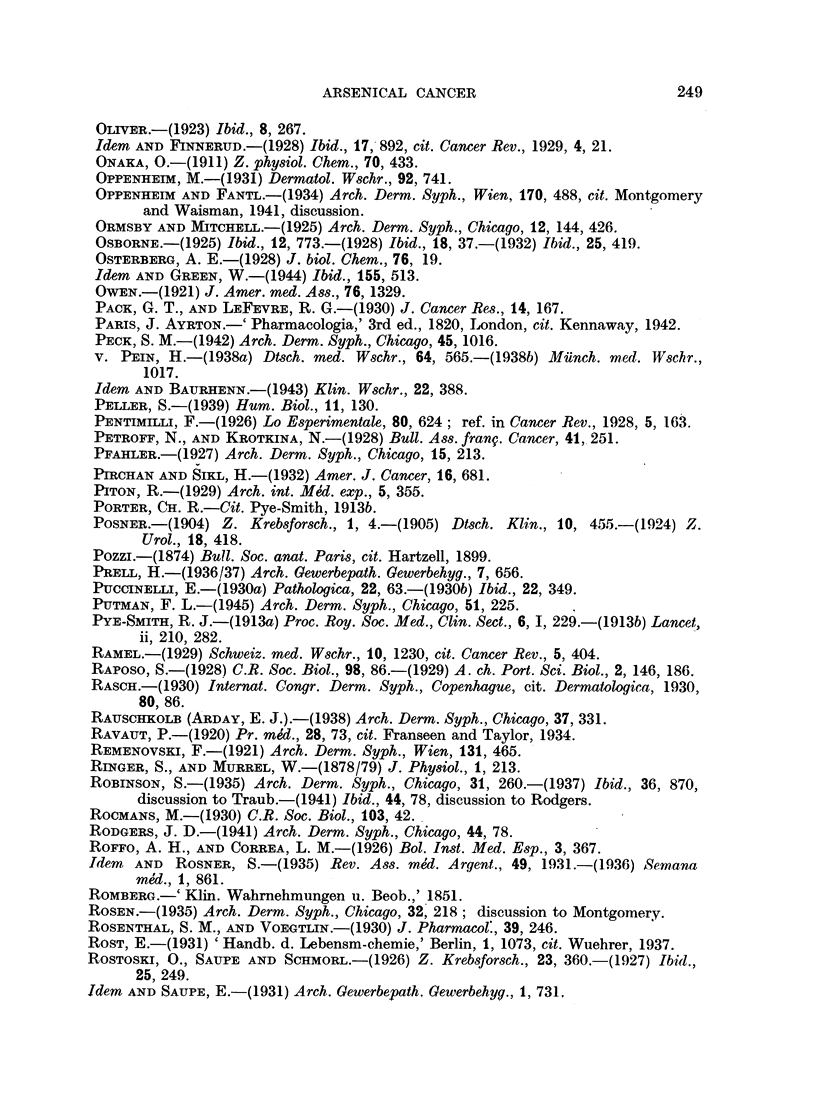

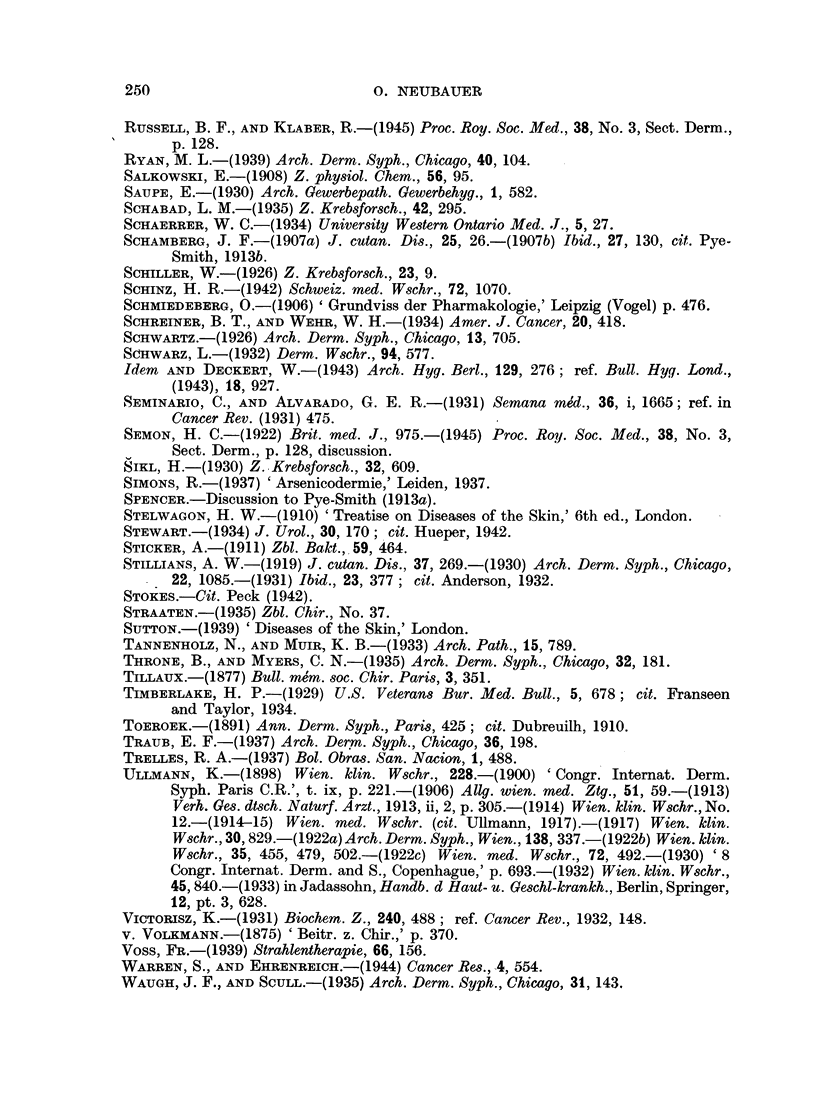

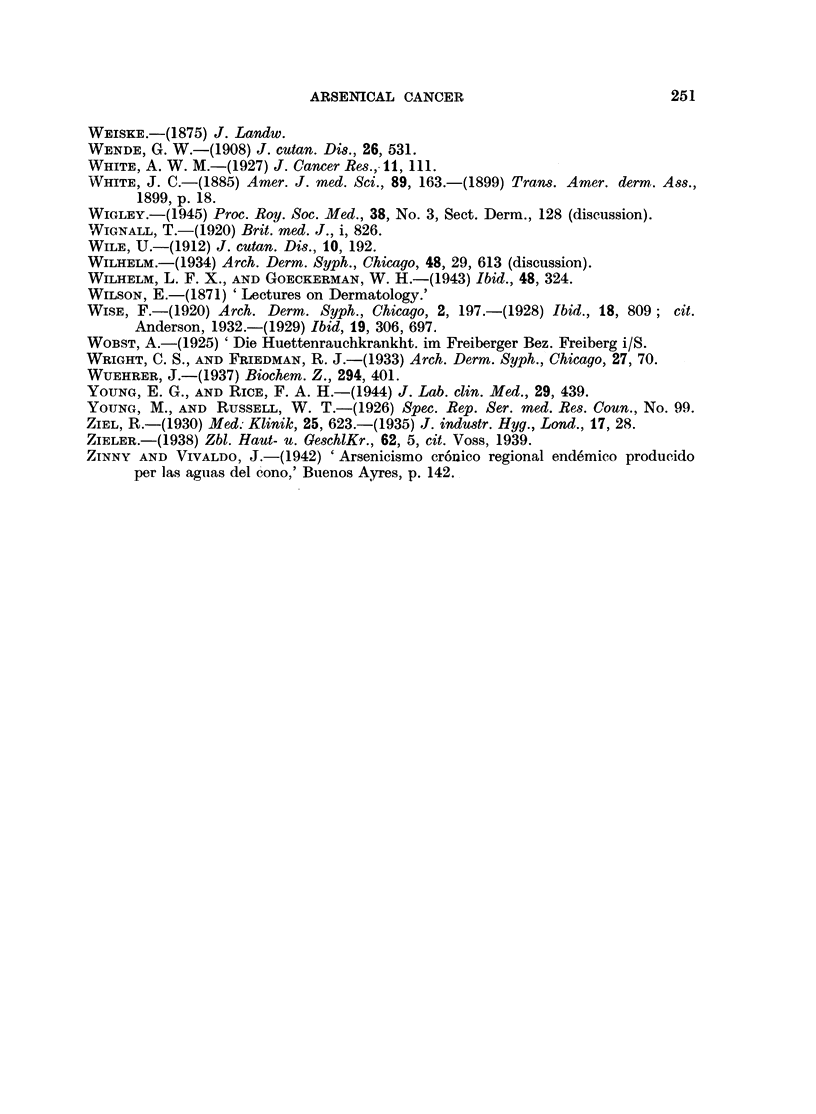


## References

[OCR_06699] Macleod J. M. (1913). Dermatitis following Large Dose of Arsenic.. Proc R Soc Med.

[OCR_06702] McNeer G. (1934). Arsenical Keratoses and Epitheliomas.. Ann Surg.

[OCR_06791] Pye-Smith R. J. (1913). Arsenic Cancer, with Description of a Case.. Proc R Soc Med.

[OCR_06808] Ringer S., Murrell W. (1878). The Action of Arseniate of Soda and Arsenious Acid on Frogs.. J Physiol.

